# Supplementary material for: Structural insights into human zinc transporter ZnT1 mediated Zn2+ efflux
Source: EMBO Rep. 2024 Oct 10;25(11):5006–25. doi: 10.1038/s44319-024-00287-3 (PMC11549101; doi:10.1038/s44319-024-00287-3)
Supplement: Supplementary file 1 — Table EV1 [file 44319_2024_287_MOESM1_ESM.docx]

**Table EV1. Cryo-EM data collection and refinement statistics.**

|  | **ZnT1 Apo** | **ZnT1 +Zn^2+^** | | | **ZnT3 +Zn^2+^** |
| --- | --- | --- | --- | --- | --- |
| **Data collection and processing** |  |  |  |  |  |
| Magnification | 105,000 | 105,000 | 105,000 | 130,000 | 105,000 |
| Voltage (kV) | 300 | 300 | 300 | 300 | 300 |
| Electron exposure (e^-^/Å^2^) | 52 | 62.4 | 62.4 | 50 | 52 |
| Defocus range (μm) | -1.2 to -2.5 | -1.2 to -2.5 | -1.2 to -2.5 | -2.0 | -1.2 to -2.5 |
| Pixel size (Å) | 0.832 | 0.832 | 0.832 | 0.932 | 0.832 |
| Symmetry imposed | C1 | C2 | C1 | C1 | C1 |
| Final particle images (no.) | 352,205 | 104,963 | 97,466 | 345,397 | 341,267 |
| Map resolution (Å)* | 3.48 | 3.64 | 4.18 | 2.65 | 3.14 |
| **Refinement** |  | **Homo-IF** | **IF/OF** | **Homo-OF (up/up)** | **Homo-IF** |
| Initial model used (PDB) | AlphaFold model | ZnT1 Apo | ZnT1 Apo | ZnT1 Apo | AlphaFold2 model |
| Map sharpening *B* factor (Å^2^) | 192.4 | 183.4 | 195.8 | 95.9 | 155.6 |
| Model composition |  |  |  |  |  |
| non-hydrogen atoms | 5,205 | 4,929 | 4,314 | 4,858 | 4,984 |
| Protein residues | 645 | 636 | 648 | 601 | 648 |
| Ligands | Zinc: 6;  Y01: 5;  AV0: 2 ** | Zinc: 8;  Y01: 3;  AV0: 2** | Zinc: 8;  Y01: 1;  AV0: 2** | Zinc: 8;  Y01: 4;  AV0: 2** | Zinc: 6;  Y01: 2;  LIG:2;  PGT:1** |
| *B* factor (Å^2^) |  |  |  |  |  |
| Protein | 57.84 | 126.14 | 272.72 | 83.42 | 101.71 |
| Ligand | 54.64 | 119.46 | 281.85 | 88.24 | 34.86 |
| R.m.s. deviations |  |  |  |  |  |
| Bond lengths (Å) | 0.003 | 0.004 | 0.003 | 0.003 | 0.003 |
| Bond angles (°) | 0.570 | 0.642 | 0.615 | 0.511 | 0.740 |
| Validation |  |  |  |  |  |
| MolProbity score | 1.66 | 1.85 | 1.81 | 1.39 | 1.61 |
| Clashscore | 6.83 | 9.02 | 8.42 | 7.13 | 6.67 |
| Poor rotamers (%) | 0 | 0 | 0 | 0 | 0 |
| Ramachandran plot |  |  |  |  |  |
| Favored (%) | 95.87 | 94.68 | 94.79 | 98.63 | 96.41 |
| Allowed (%) | 4.13 | 5.32 | 5.21 | 1.37 | 3.59 |
| Outliers (%) | 0.00 | 0.00 | 0.00 | 0.00 | 0.00 |
| Deposited model (PDB id) | 8XM6 | 8XMF | 8XMJ | 8XMA | 8XN1 |
| Deposited map (EMDB id) | 38465 | 38475 | 38479 | 38469 | 38494 |

* Gold standard FSC with threshold of 0.143

** Y01: cholesteryl hemisuccinate; AV0: lauryl maltose neopentyl glycol; LIG: lauryl maltose neopentyl glycol; PGT: phosphatidyl ethanolamine
