# Supplementary material for: Structural insights into human zinc transporter ZnT1 mediated Zn2+ efflux
Source: EMBO Rep. 2024 Oct 10;25(11):5006–25. doi: 10.1038/s44319-024-00287-3 (PMC11549101; doi:10.1038/s44319-024-00287-3)
Supplement: Supplementary file 2 — Appendix [file 44319_2024_287_MOESM2_ESM.pdf]

Appendix

Table of Content:

Pages 2-3: **Appendix Figure S1**: Cryo-EM density for hZnT1 and hZnT3 structures

Pages 4-5: **Appendix Figure S2**: Sequence alignment of ZnT1 homologs

Page 6: **Appendix Figure S3**: Sequence alignment of selected ZnT/YiiP family members

Page 7: **Appendix Figure S4**: Characterization of Zn^{2+}-bound hZnT3 in inward-facing state

Appendix Figure S1

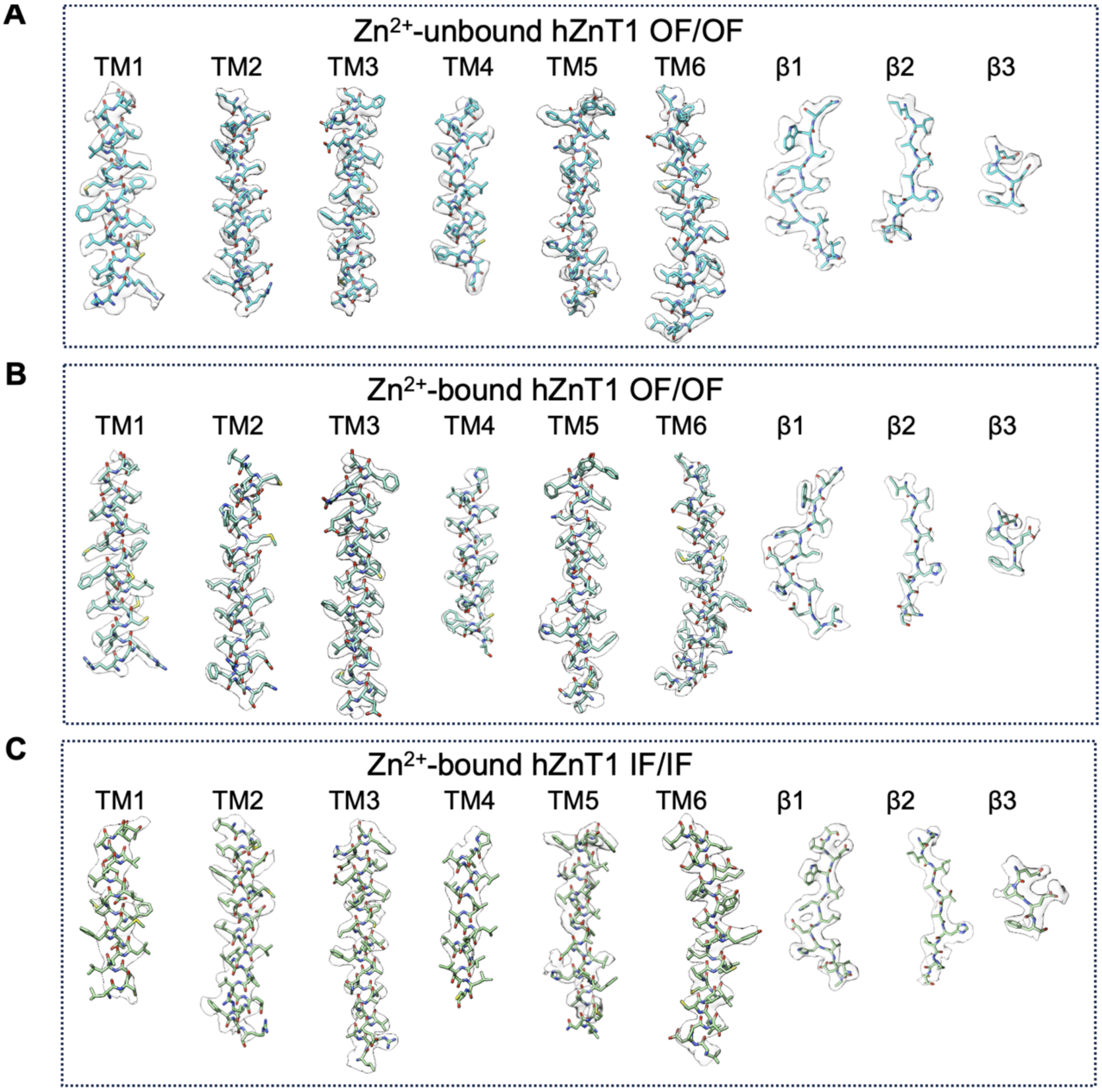

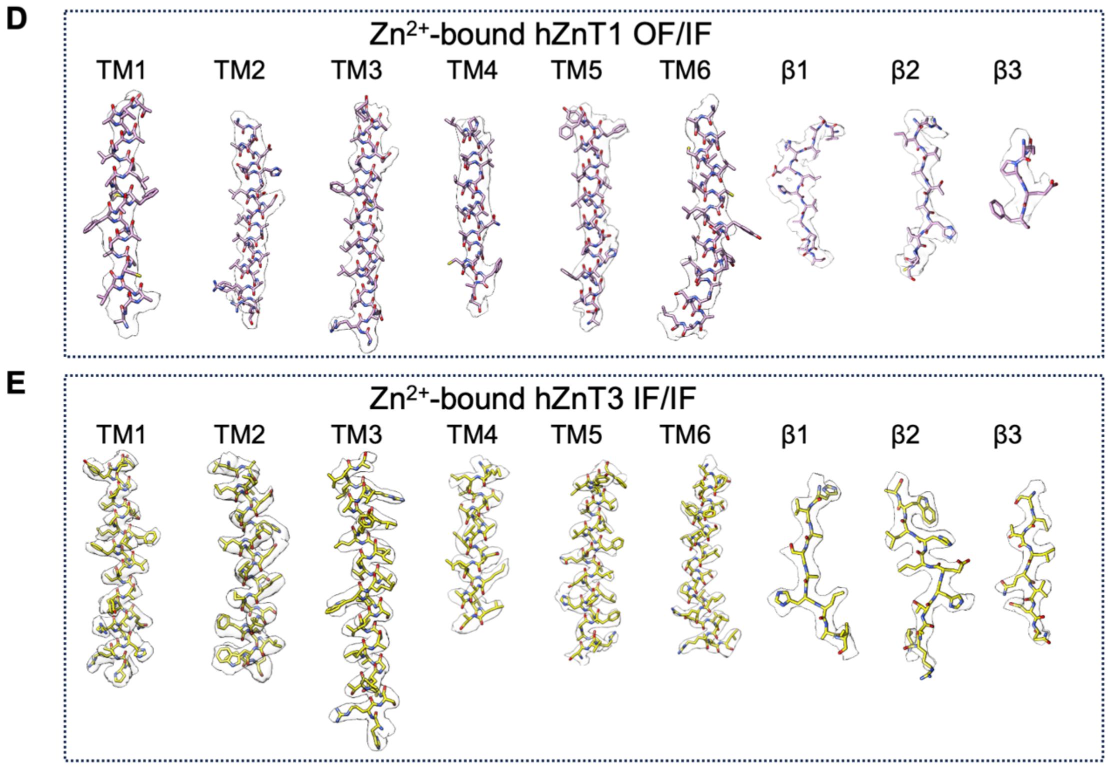

Appendix Figure S1. Cryo-EM density for hZnT1 and hZnT3 structures

A Representative model segments fitting in the density are shown for Zn^{2+}-unbound outward-facing hZnT1.

B Representative model segments fitting in the density are shown for Zn^{2+}-bound outward-facing hZnT1.

C Representative model segments fitting in the density are shown for Zn^{2+}-bound inward-facing hZnT1.

D Representative model segments fitting in the density are shown for Zn^{2+}-bound heterodimeric hZnT1.

E Representative model segments fitting in the density are shown for Zn^{2+}-bound inward-facing hZnT3.

Appendix Figure S2

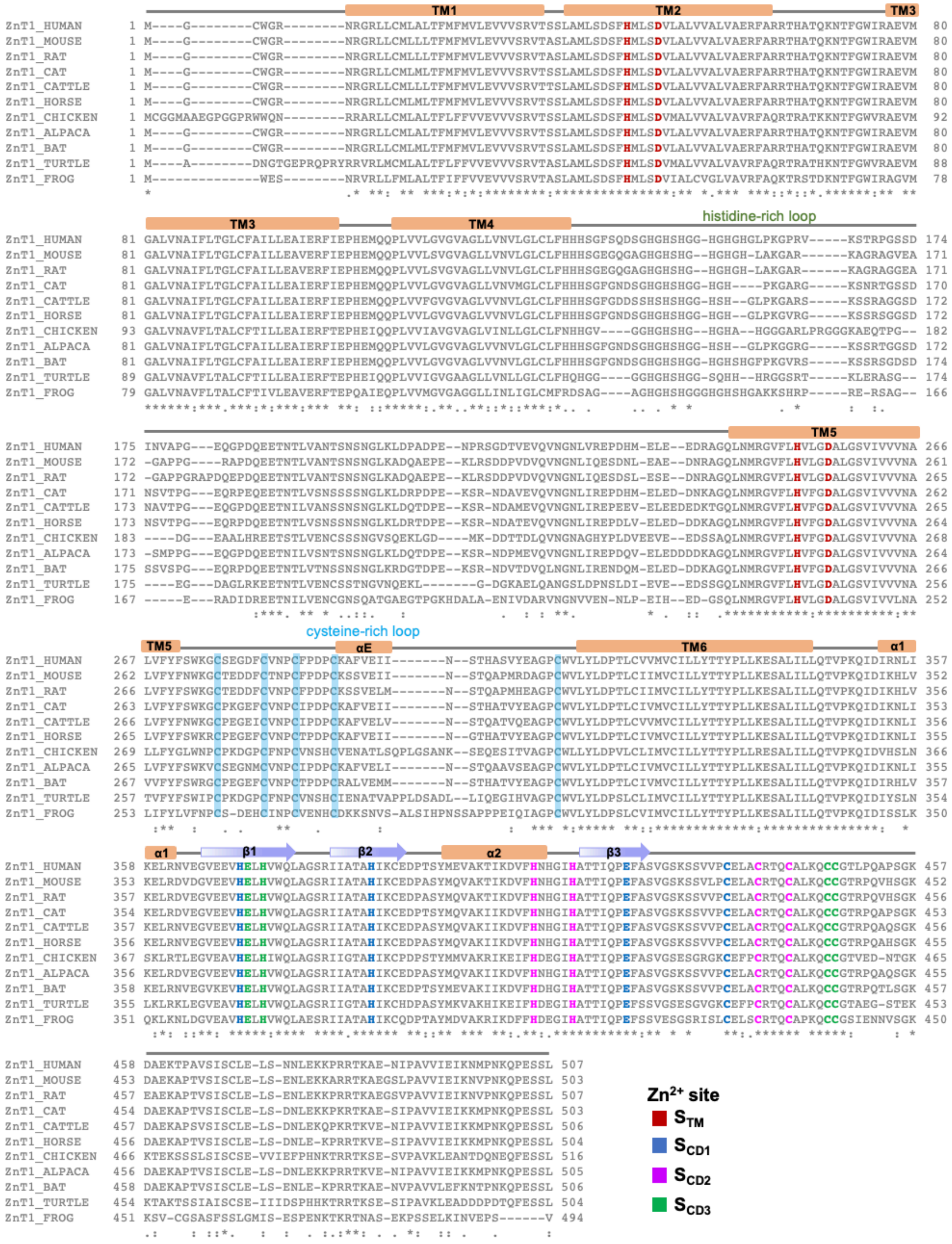

Appendix Figure S2. Sequence alignment of ZnT1 homologs

Sequences of ZnT1 homologues are aligned from human (Q9Y6M5), mouse (Q60738), rat (Q62720), cat (A0A337SGH3), cattle (E1BN60), horse (F7DUJ0), chicken (F1NHZ7), alpaca (A0A6I9HXF2), bat (A0A8B7QS86), turtle (A0A8T1SIR4), frog(Q7ZTK1) to show the conservation across evolution. The secondary structure of ZnT1 is listed above the sequences. Residues

responsible for Zn^{2+} coordination are colored accordingly. Cysteine residues rich in the extracellular loop between TM5 and TM6 are labeled respectively.

Appendix Figure S3

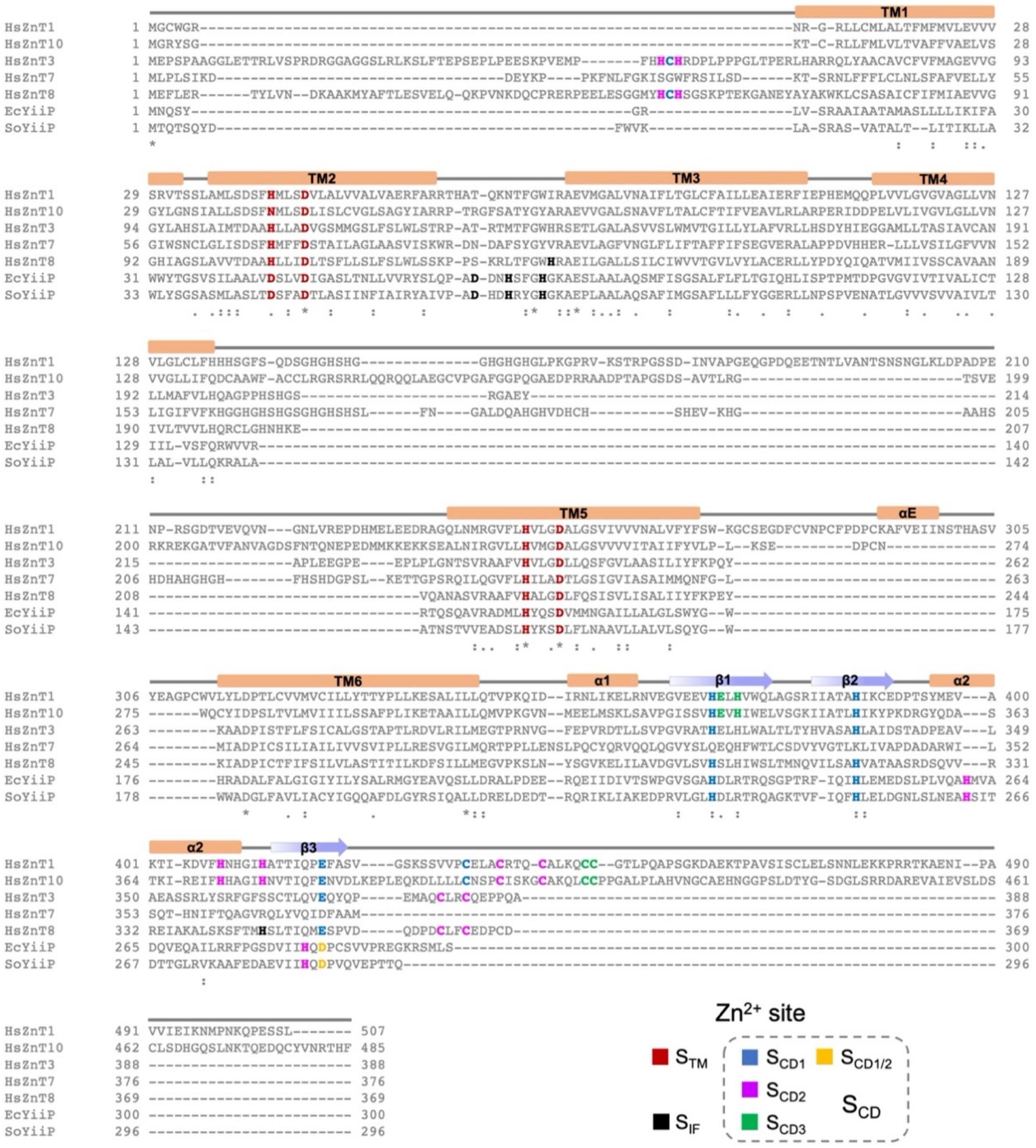

Appendix Figure S3. Sequence alignment of selected ZnT/YiiP family members

Sequences of ZnT/YiiP family members with structures available, except ZnT10, are aligned to reflect the conserved and distinct features. The family members selected are human ZnT1 (Q9Y6M5), ZnT10 (Q6XR72), ZnT3 (Q99726), ZnT7 (Q8NEW0), ZnT8 (Q8IWU4), and two bacterial YiiP, Escherichia coli (EcYiiP: P69380) and Shewanella oneidensis (SoYiiP: Q8E919). The secondary structure of HsZnT1 is listed above the sequences. Residues responsible for Zn^{2+} coordination are colored separately for different binding sites.

Appendix Figure S4

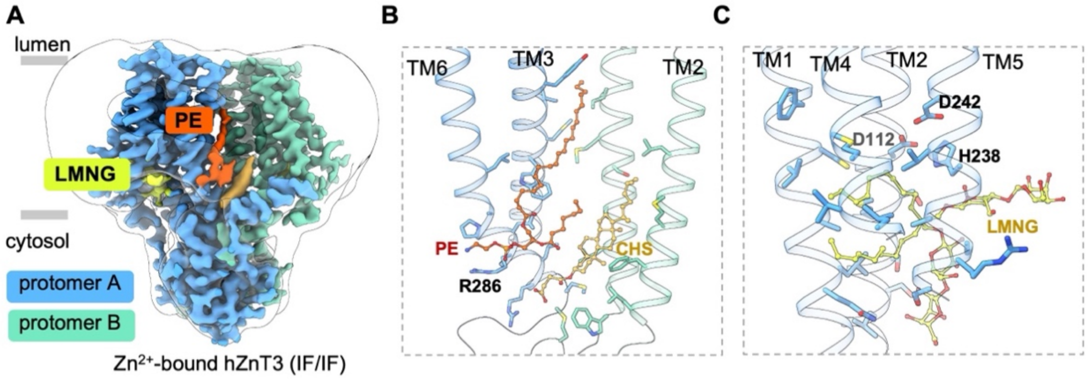

Appendix Figure S4. Characterization of Zn^{2+}-bound hZnT3 in inward-facing state

A The 3.14-Å cryo-EM map viewed from membrane plane, with lipid-like densities colored differently.

B Possible phosphoethanolamine molecule is wedged between two TMDs shown in sticks.

C LMNG molecule is engaged in the translocation funnel, close to the Zn^{2+}-binding site.
