# Supplementary figures and images for: Structural insights into human zinc transporter ZnT1 mediated Zn2+ efflux
Source: EMBO Rep. 2024 Oct 10;25(11):5006–25. doi: 10.1038/s44319-024-00287-3 (PMC11549101; doi:10.1038/s44319-024-00287-3)

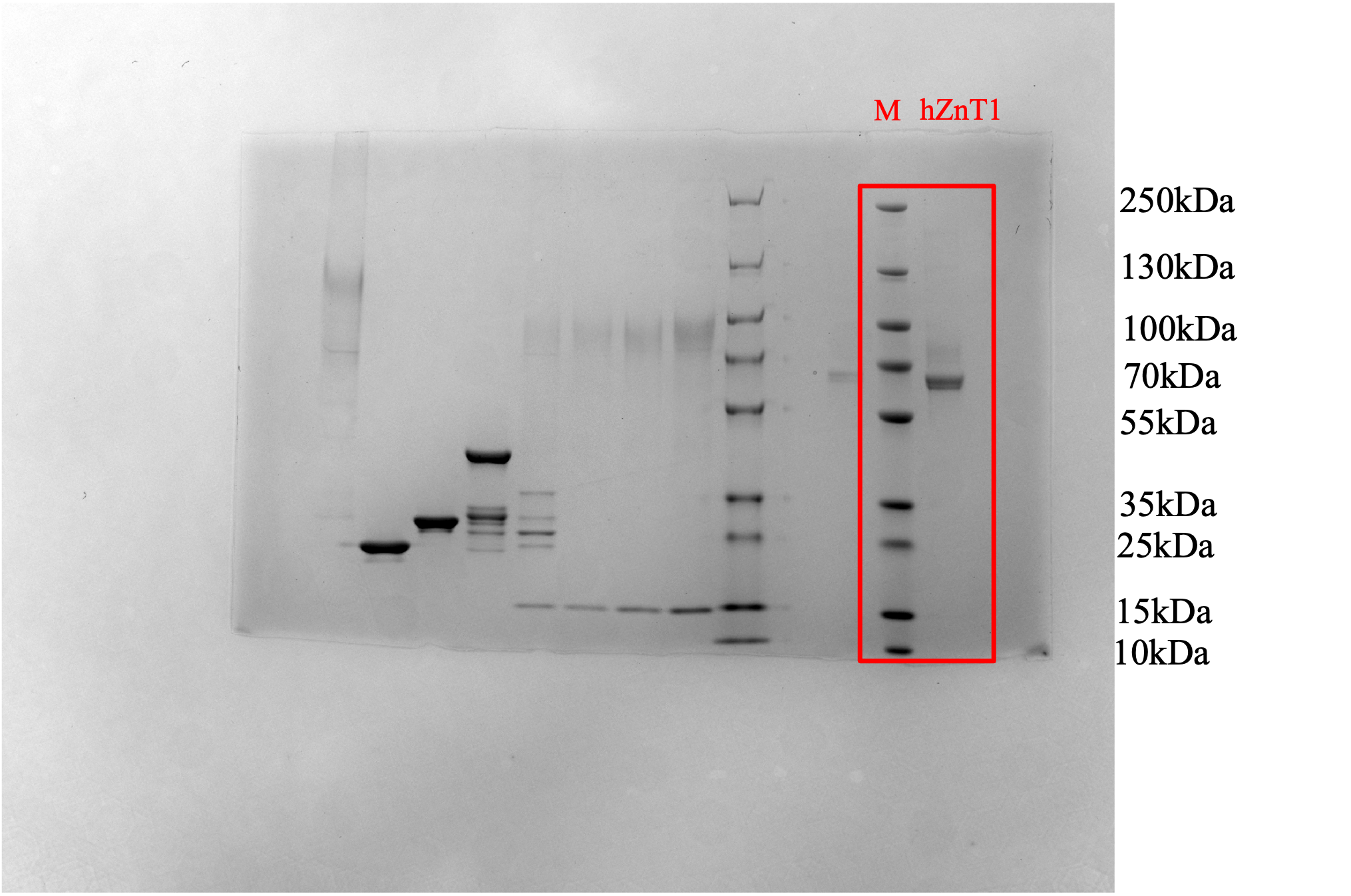

Supplement: Supplementary file 11 — Source data Fig. 1B [file 44319_2024_287_MOESM11_ESM.zip › Figure1B/ZnT1 WT-SDS PAGE cropping area.png]

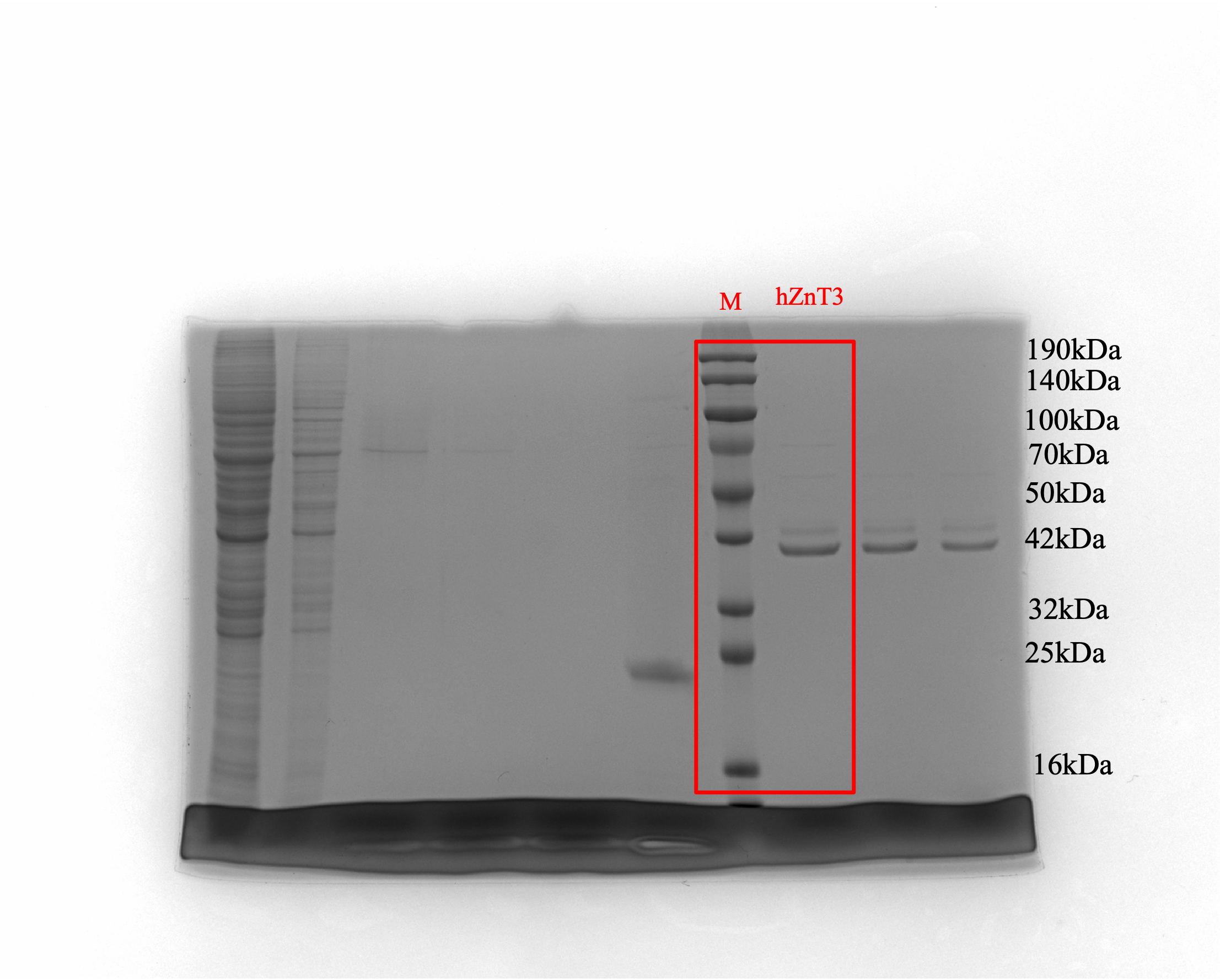

Supplement: Supplementary file 11 — Source data Fig. 1B [file 44319_2024_287_MOESM11_ESM.zip › Figure1B/ZnT3 WT-SDS PAGE cropping area.png]

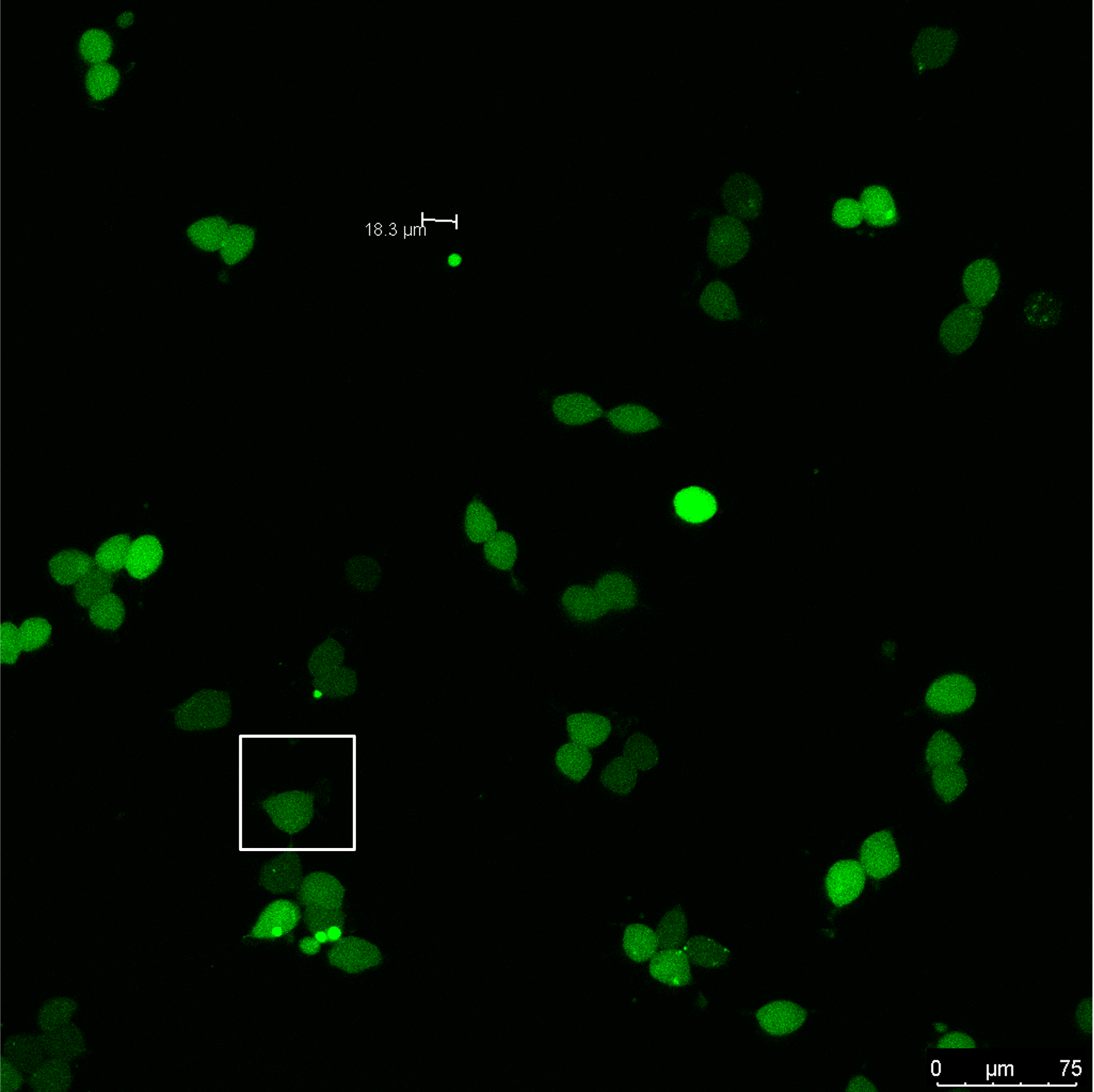

Supplement: Supplementary file 13 — Appendix and EV Figures Source Data [file 44319_2024_287_MOESM13_ESM.zip › FigureEV4A/Confocal image/D47N_D255N/D47N_D255N_ch00.png]

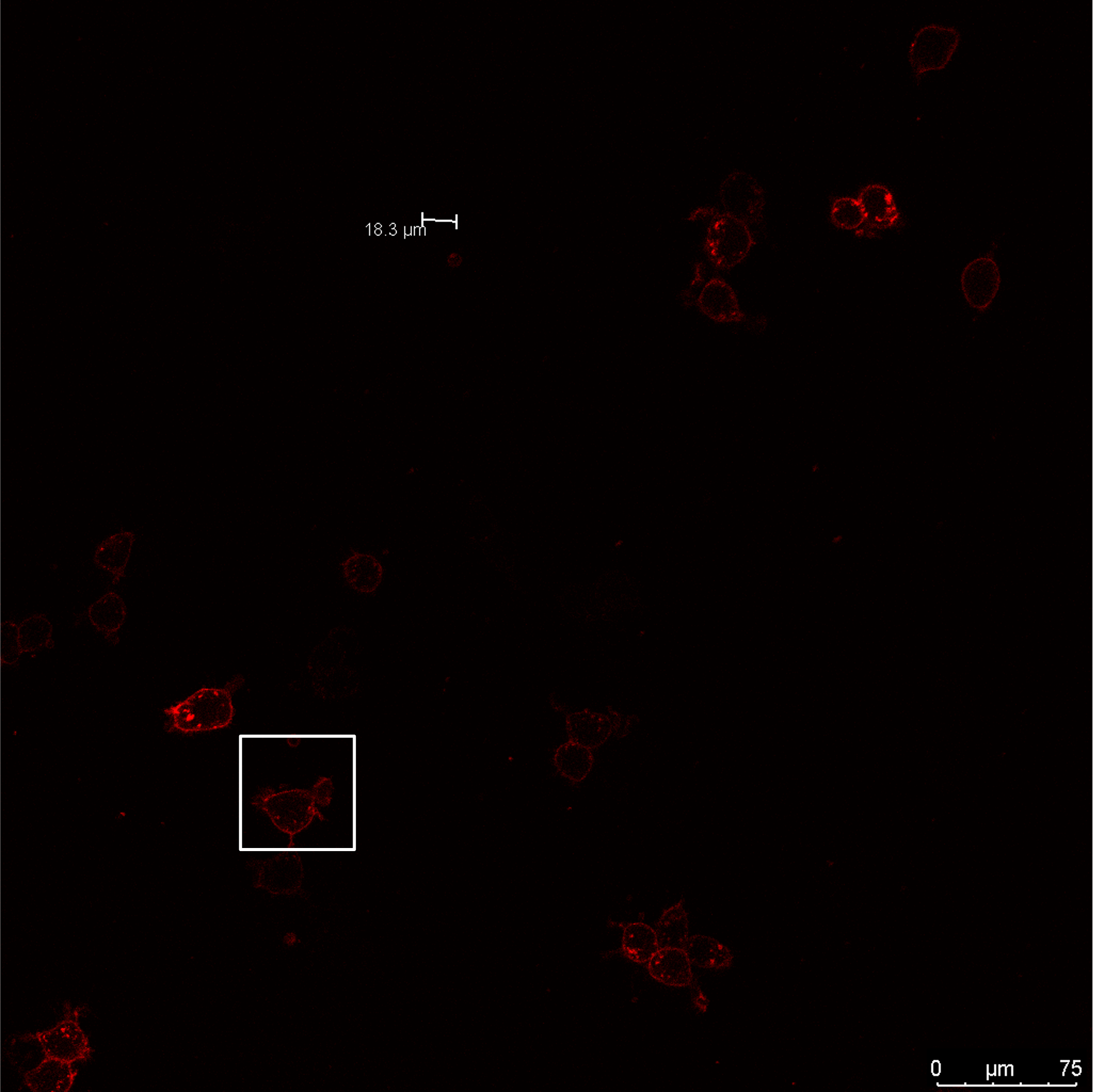

Supplement: Supplementary file 13 — Appendix and EV Figures Source Data [file 44319_2024_287_MOESM13_ESM.zip › FigureEV4A/Confocal image/D47N_D255N/D47N_D255N_ch01.png]

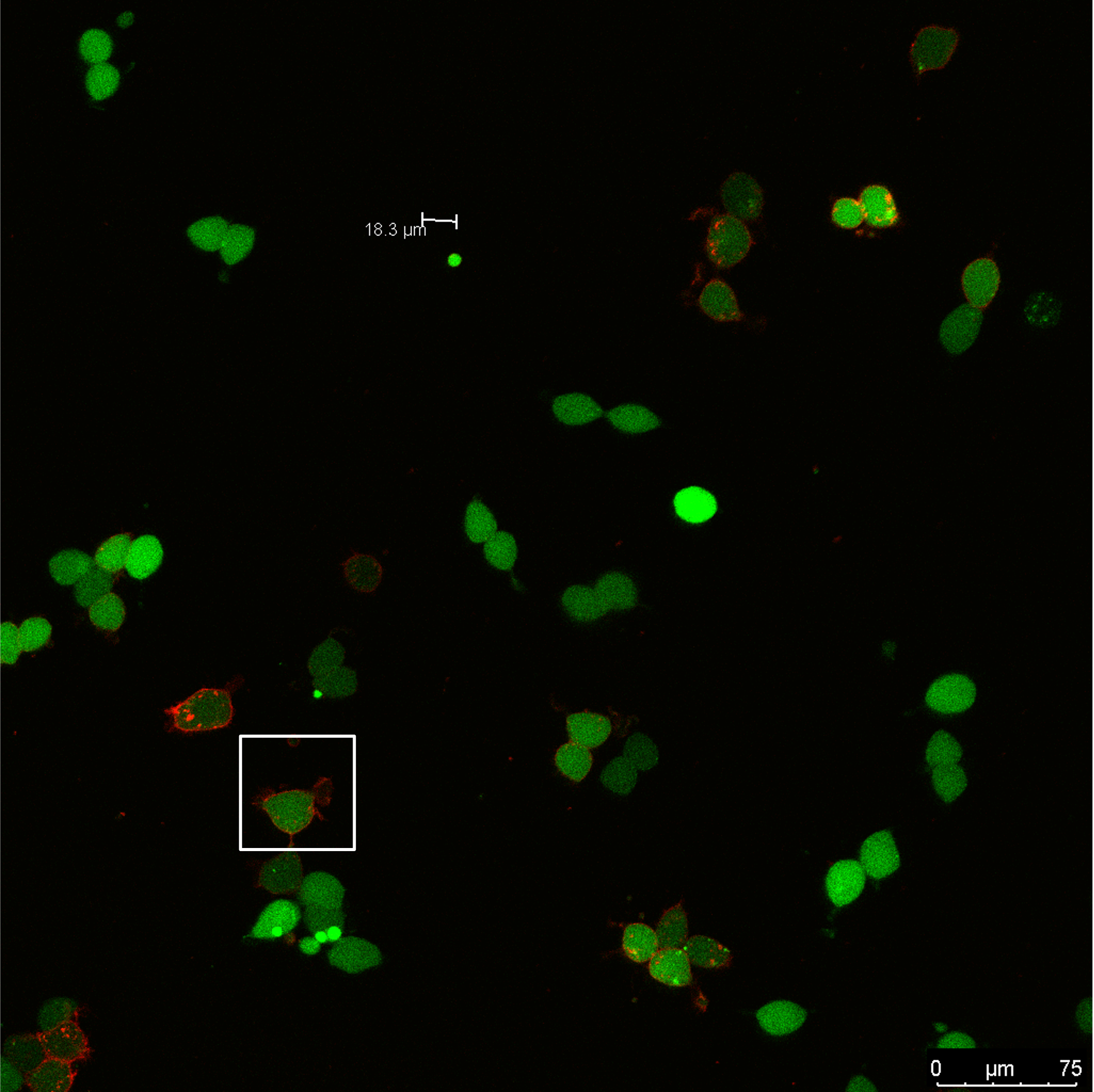

Supplement: Supplementary file 13 — Appendix and EV Figures Source Data [file 44319_2024_287_MOESM13_ESM.zip › FigureEV4A/Confocal image/D47N_D255N/D47N_D255N_merge.png]

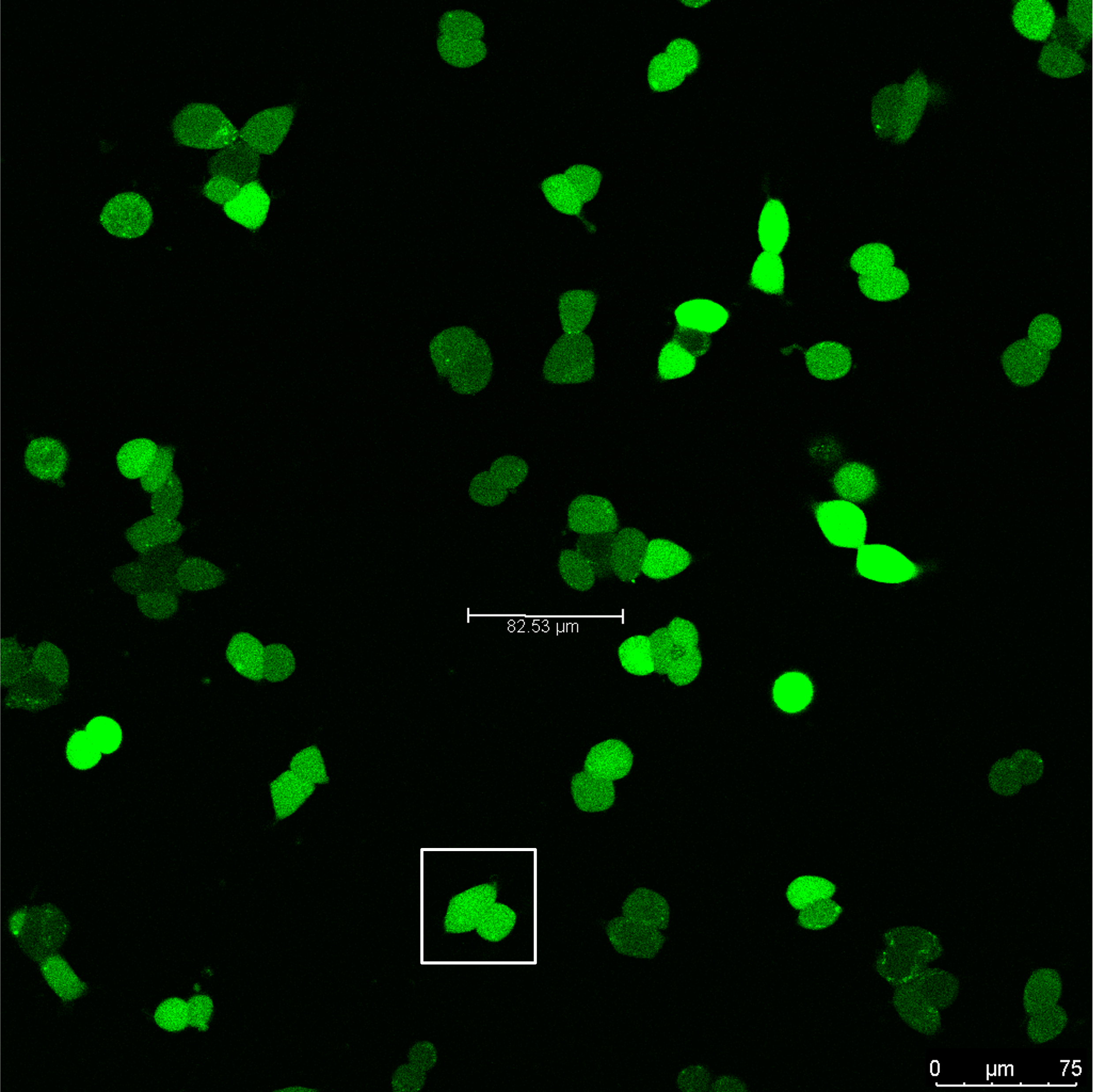

Supplement: Supplementary file 13 — Appendix and EV Figures Source Data [file 44319_2024_287_MOESM13_ESM.zip › FigureEV4A/Confocal image/Empty Vector/Empty Vector_ch00.png]

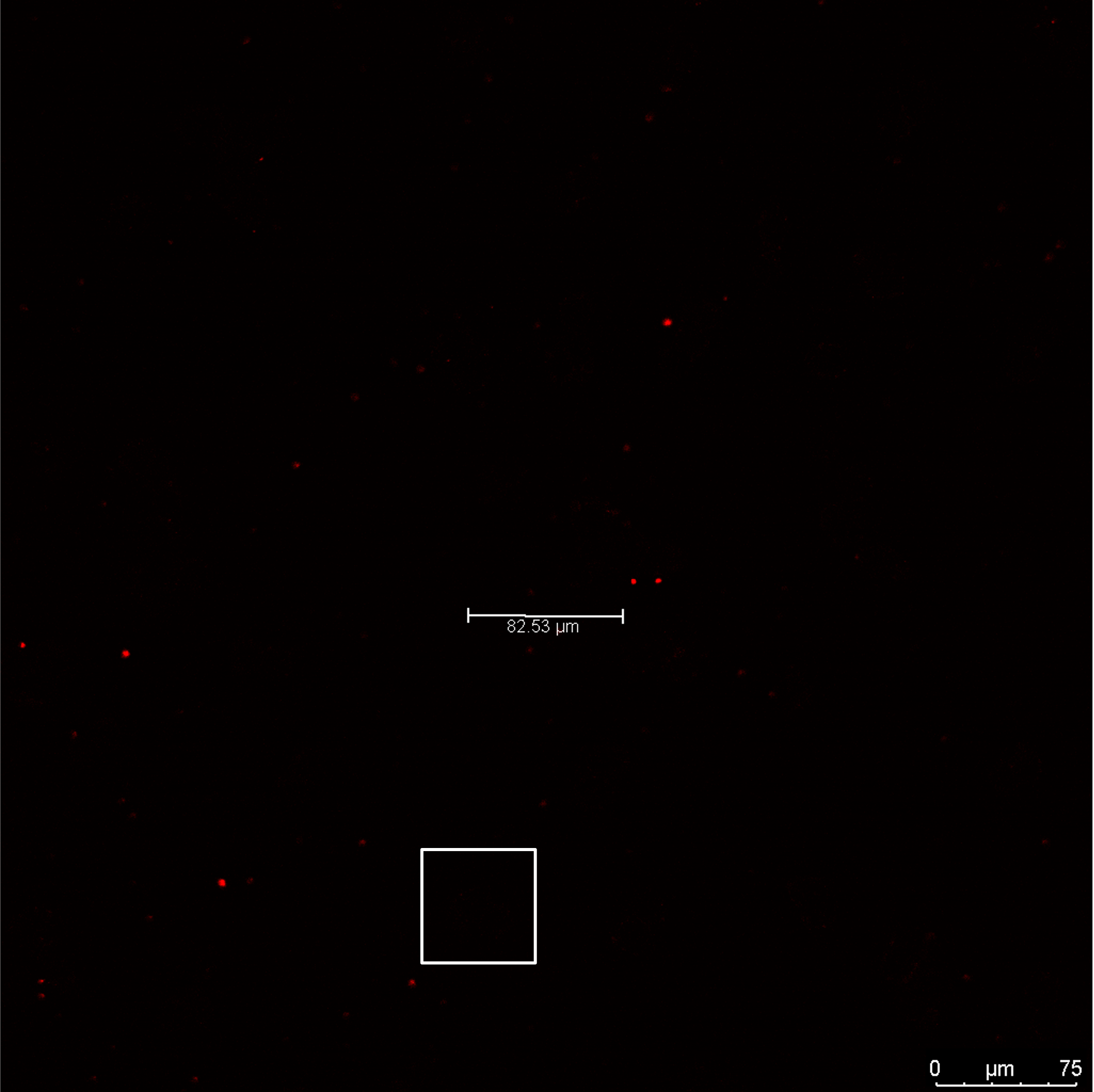

Supplement: Supplementary file 13 — Appendix and EV Figures Source Data [file 44319_2024_287_MOESM13_ESM.zip › FigureEV4A/Confocal image/Empty Vector/Empty Vector_ch01.png]

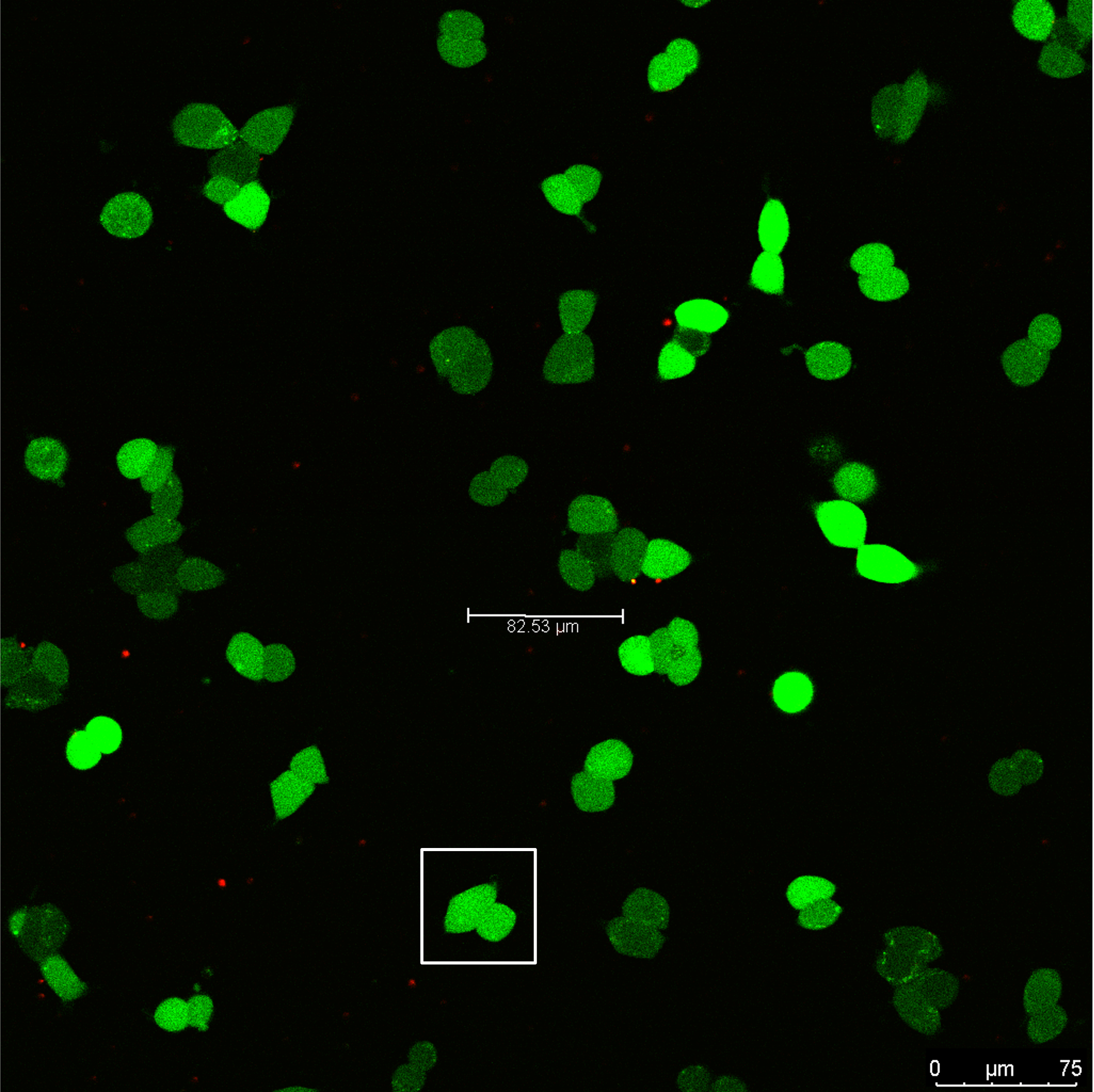

Supplement: Supplementary file 13 — Appendix and EV Figures Source Data [file 44319_2024_287_MOESM13_ESM.zip › FigureEV4A/Confocal image/Empty Vector/Empty Vector_merge.png]

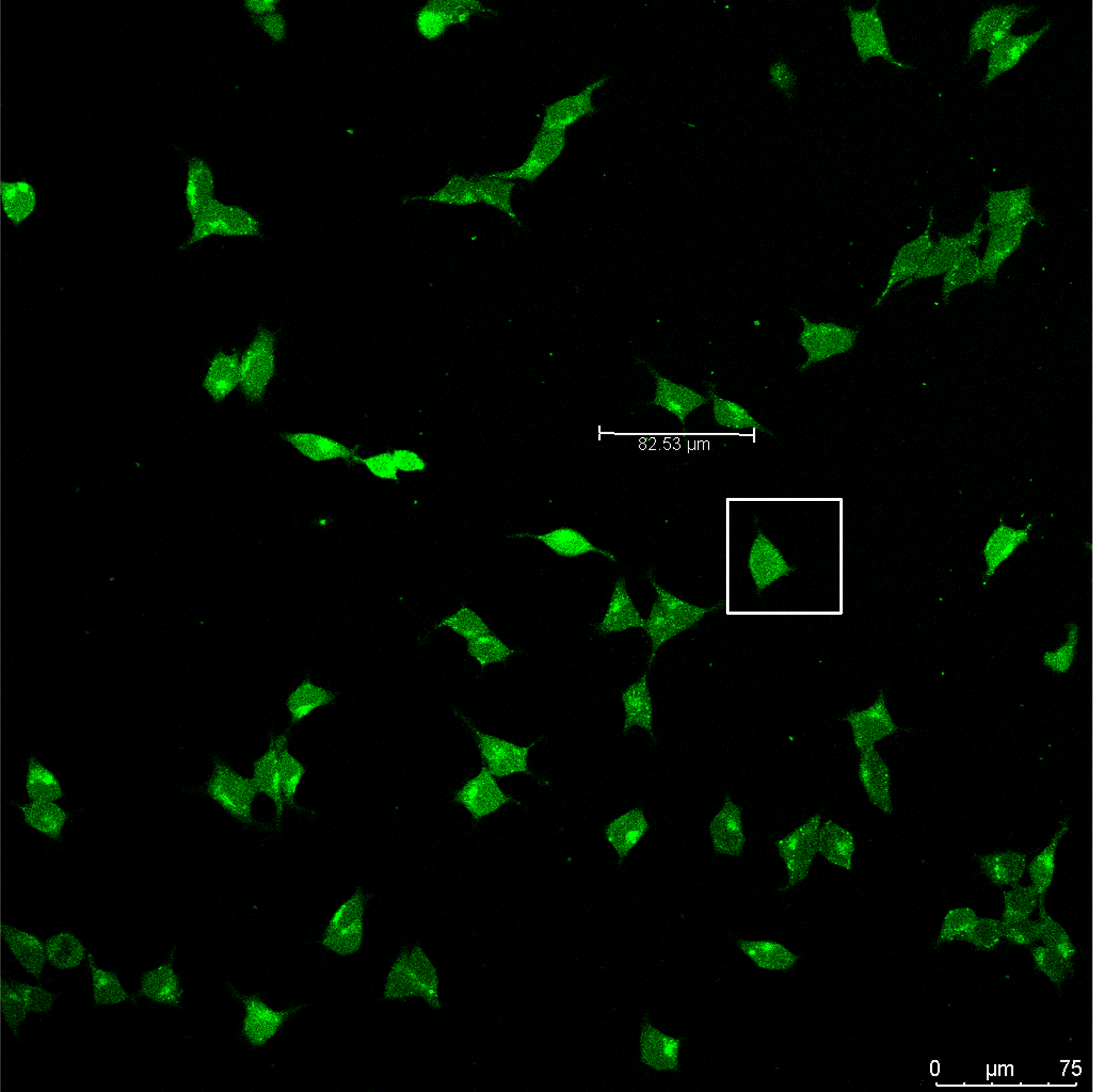

Supplement: Supplementary file 13 — Appendix and EV Figures Source Data [file 44319_2024_287_MOESM13_ESM.zip › FigureEV4A/Confocal image/H43A/H43A_ch00.png]

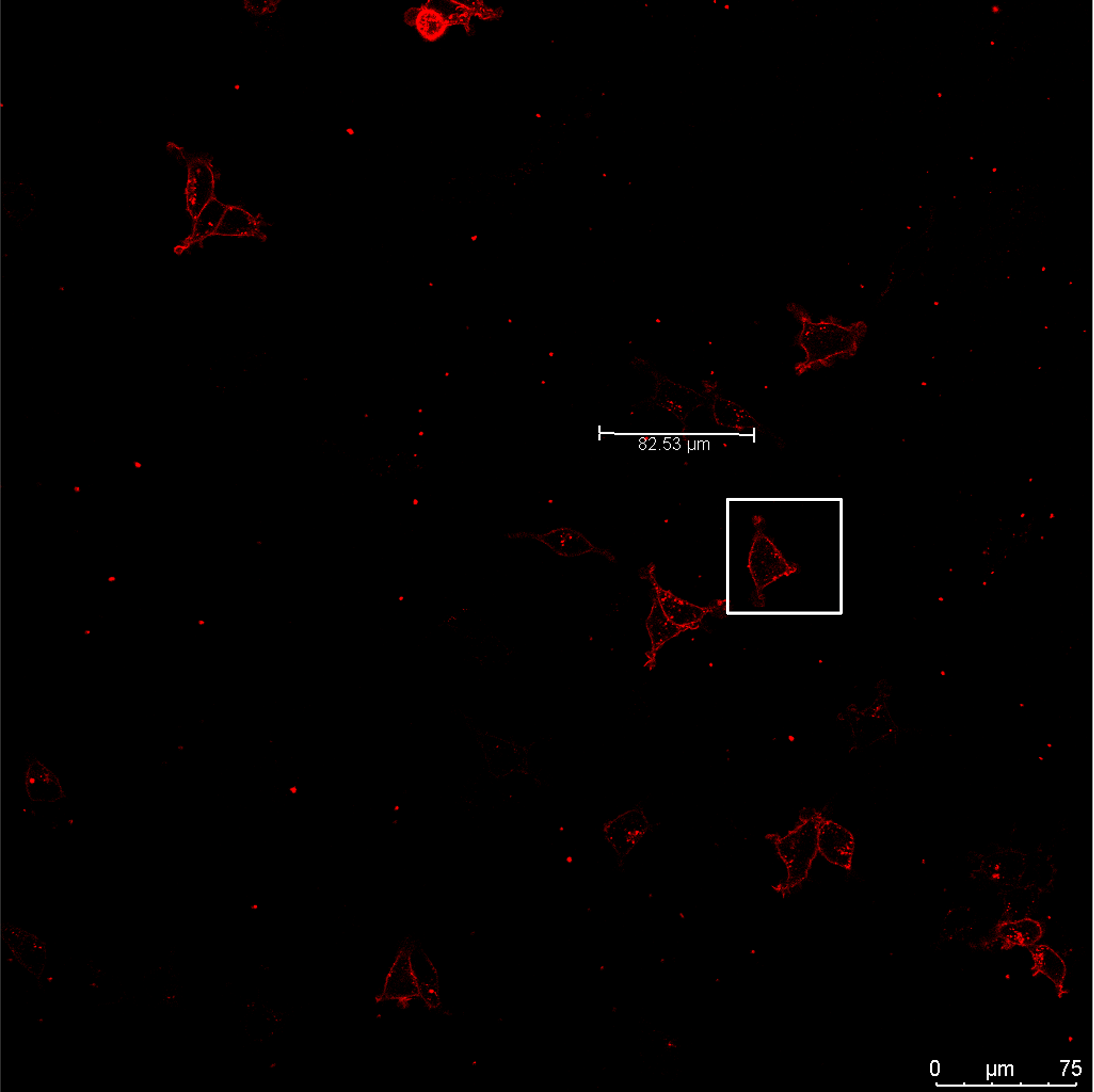

Supplement: Supplementary file 13 — Appendix and EV Figures Source Data [file 44319_2024_287_MOESM13_ESM.zip › FigureEV4A/Confocal image/H43A/H43A_ch01.png]

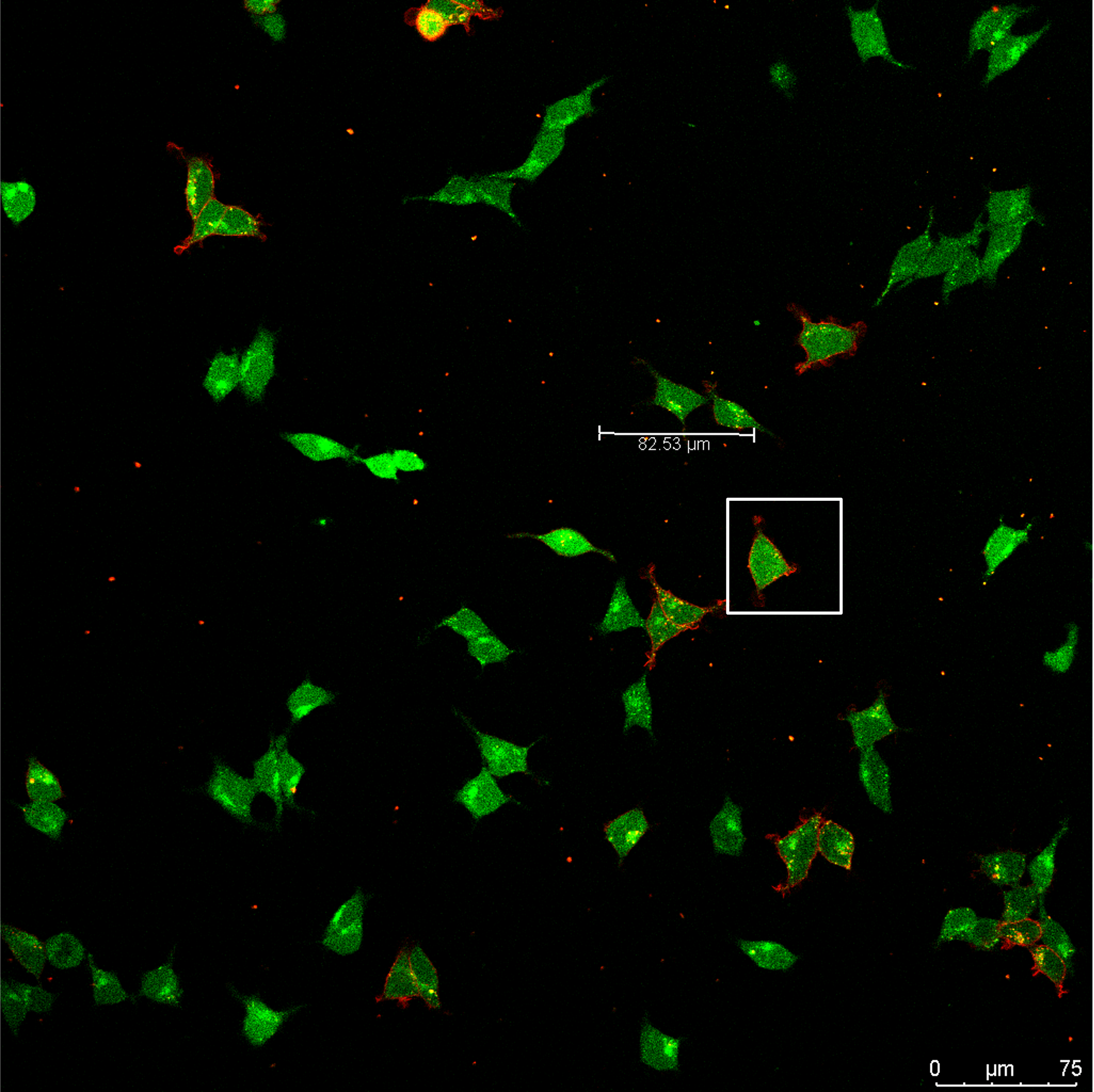

Supplement: Supplementary file 13 — Appendix and EV Figures Source Data [file 44319_2024_287_MOESM13_ESM.zip › FigureEV4A/Confocal image/H43A/H43A_merge.png]

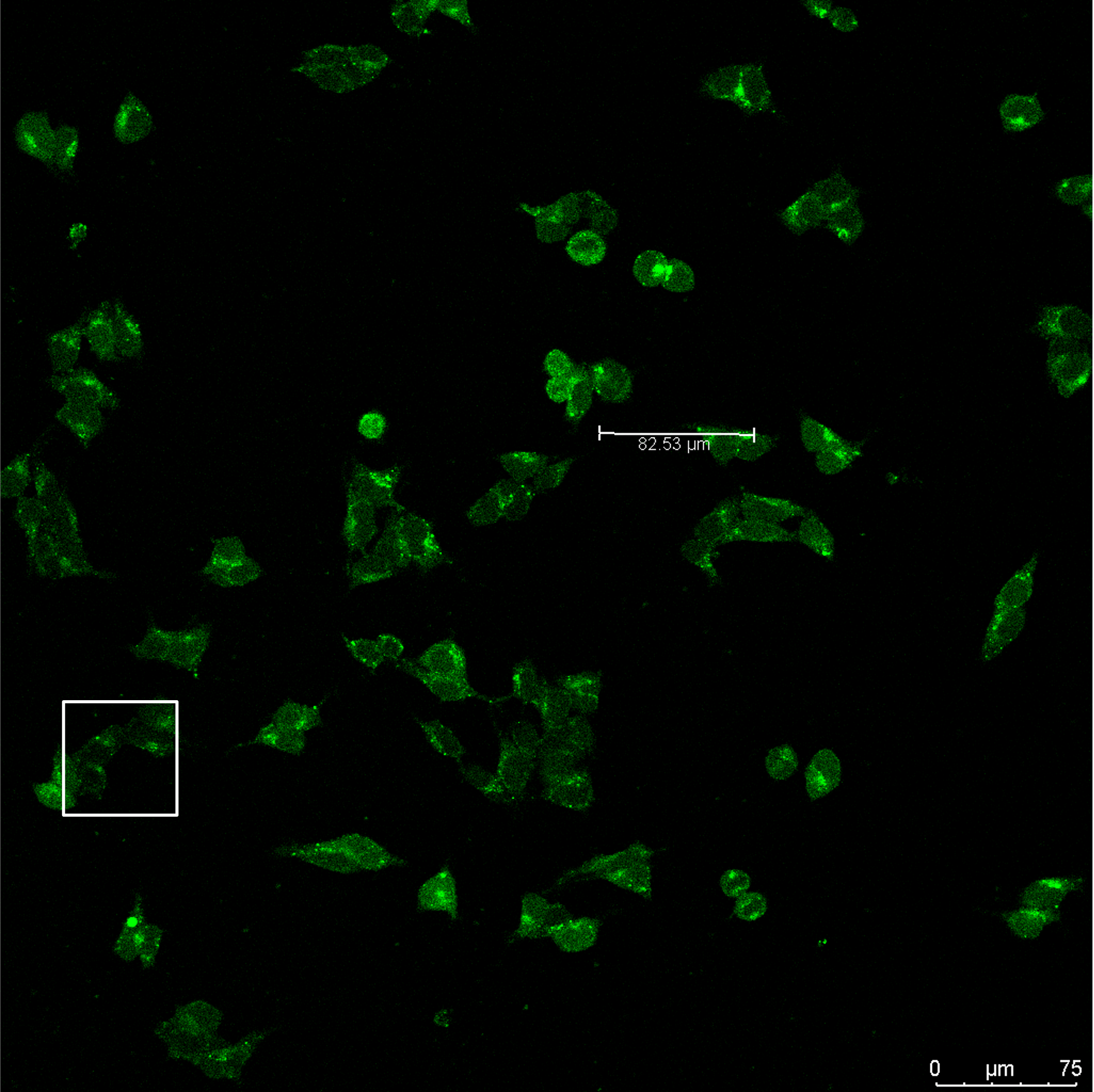

Supplement: Supplementary file 13 — Appendix and EV Figures Source Data [file 44319_2024_287_MOESM13_ESM.zip › FigureEV4A/Confocal image/H43D/H43D_ch00.png]

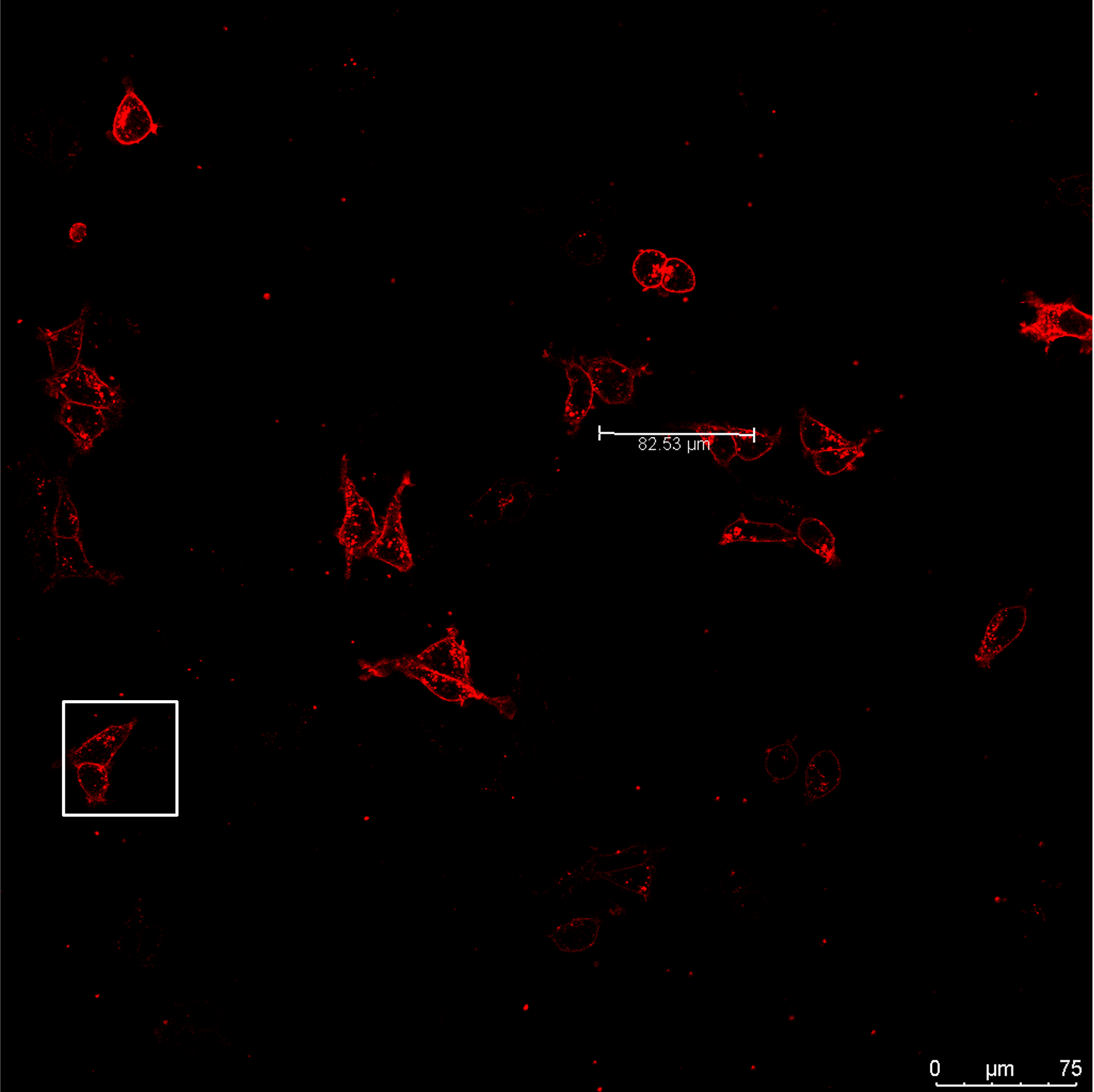

Supplement: Supplementary file 13 — Appendix and EV Figures Source Data [file 44319_2024_287_MOESM13_ESM.zip › FigureEV4A/Confocal image/H43D/H43D_ch01.png]

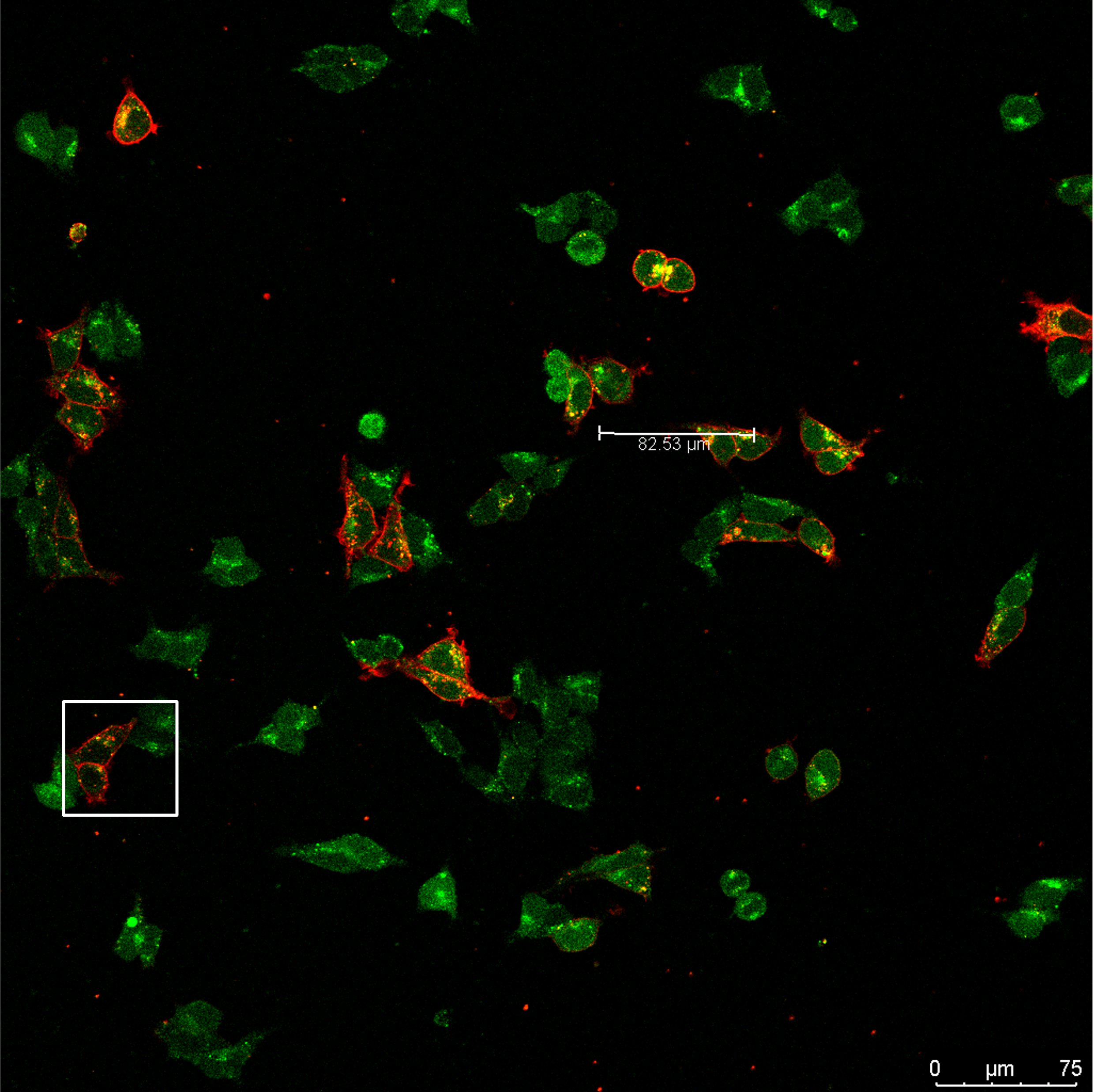

Supplement: Supplementary file 13 — Appendix and EV Figures Source Data [file 44319_2024_287_MOESM13_ESM.zip › FigureEV4A/Confocal image/H43D/H43D_merge.png]

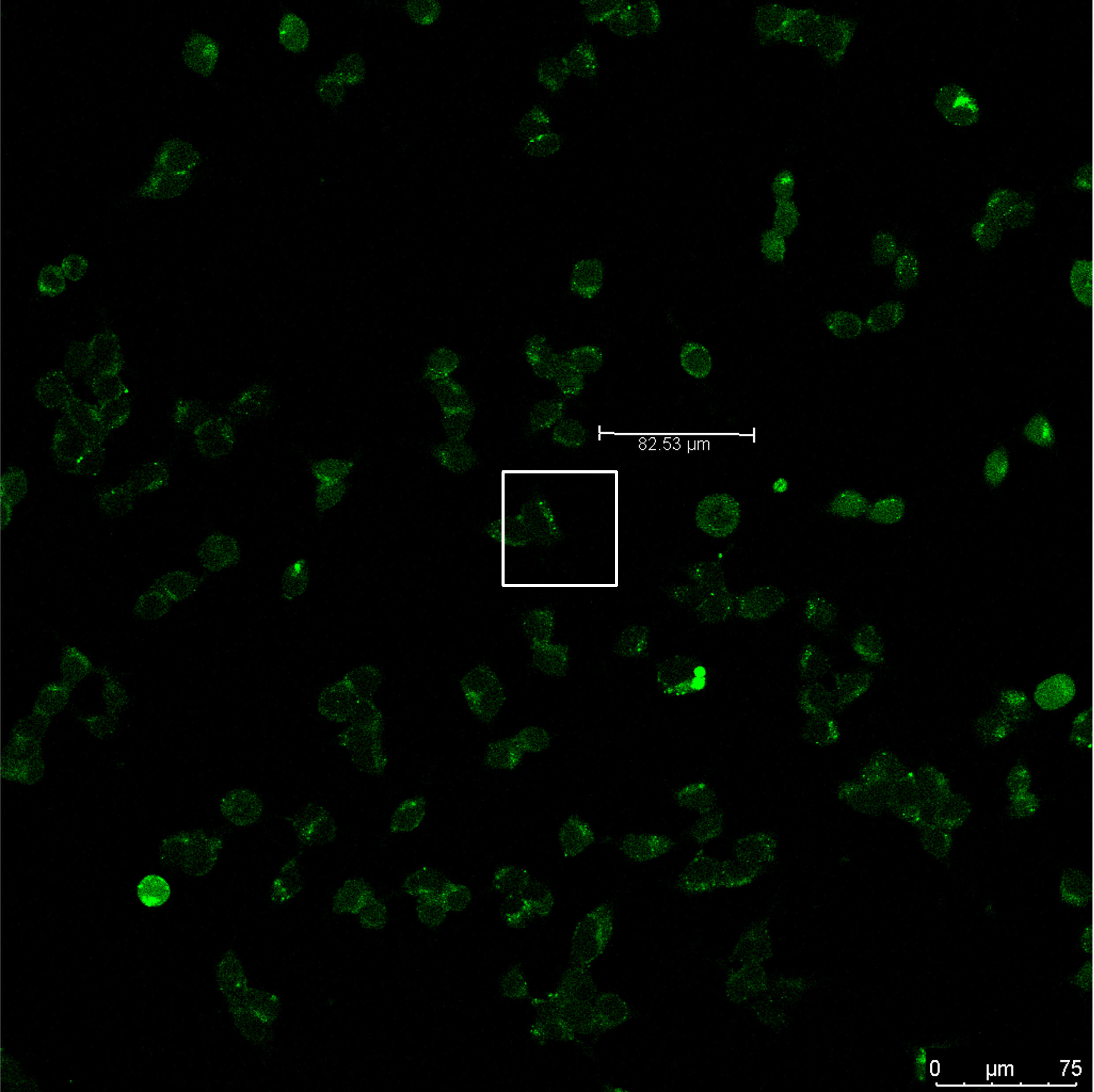

Supplement: Supplementary file 13 — Appendix and EV Figures Source Data [file 44319_2024_287_MOESM13_ESM.zip › FigureEV4A/Confocal image/H43N/H43N_ch00.png]

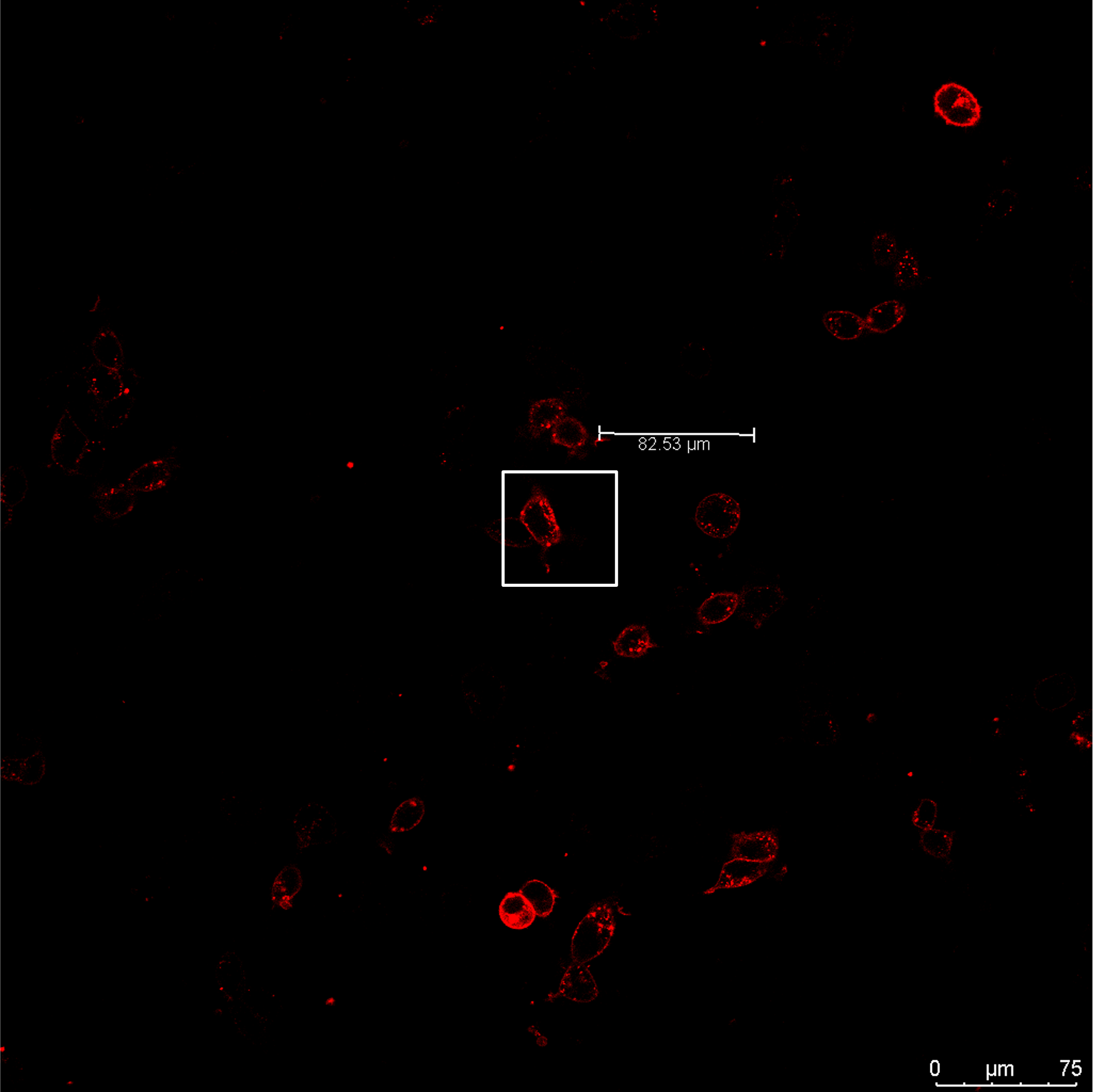

Supplement: Supplementary file 13 — Appendix and EV Figures Source Data [file 44319_2024_287_MOESM13_ESM.zip › FigureEV4A/Confocal image/H43N/H43N_ch01.png]

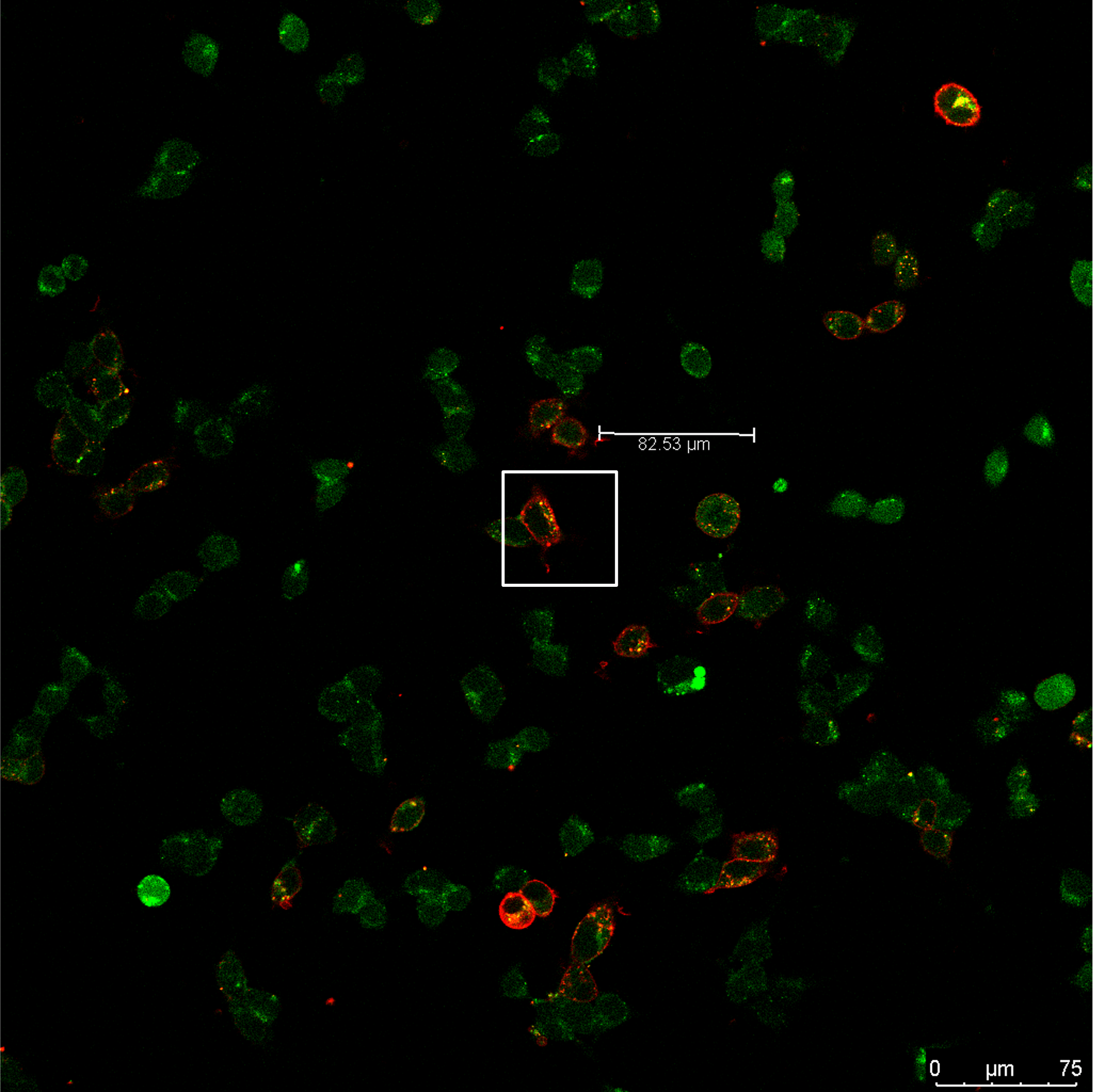

Supplement: Supplementary file 13 — Appendix and EV Figures Source Data [file 44319_2024_287_MOESM13_ESM.zip › FigureEV4A/Confocal image/H43N/H43N_merge.png]

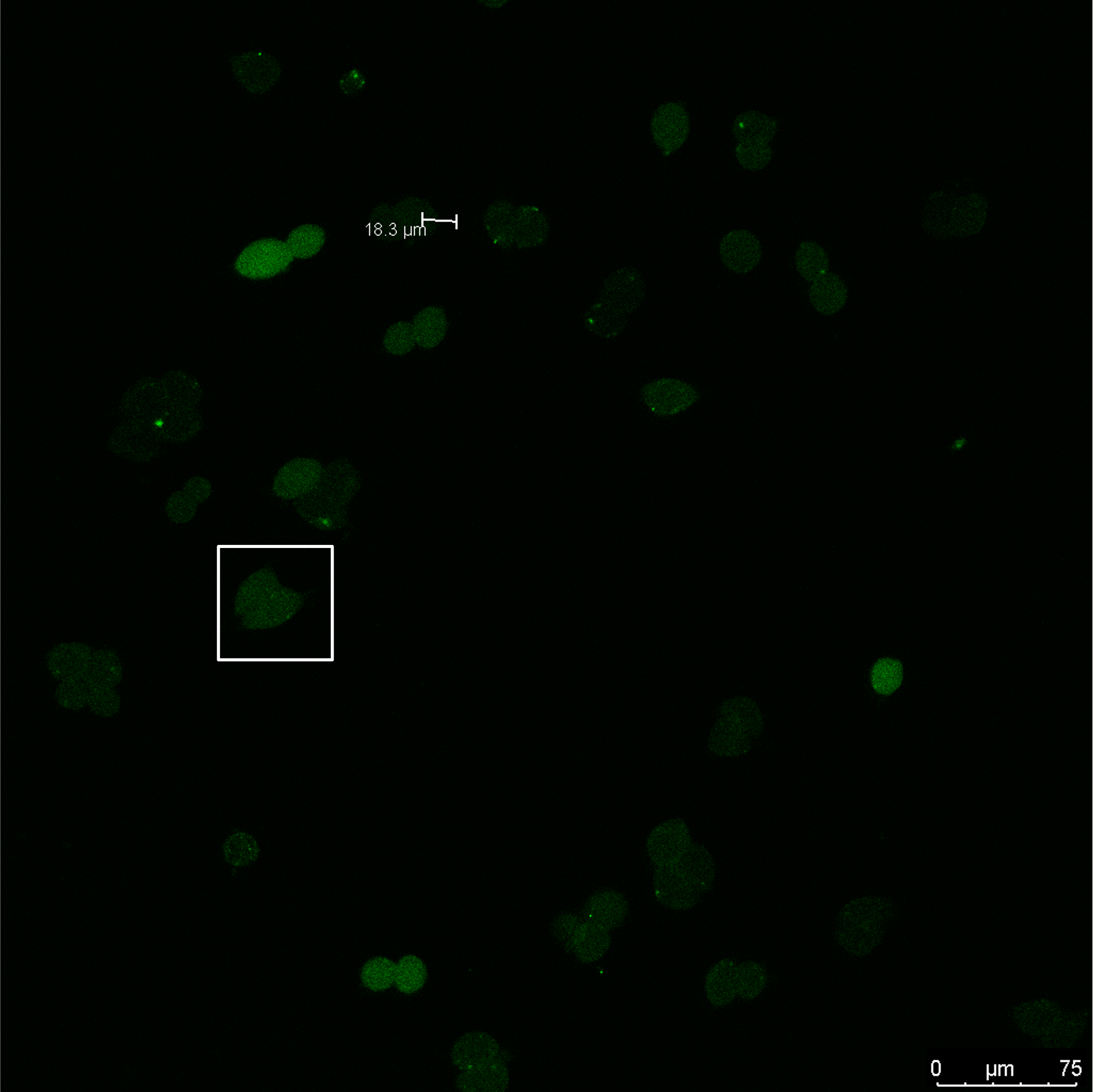

Supplement: Supplementary file 13 — Appendix and EV Figures Source Data [file 44319_2024_287_MOESM13_ESM.zip › FigureEV4A/Confocal image/WT/WT_ch00.png]

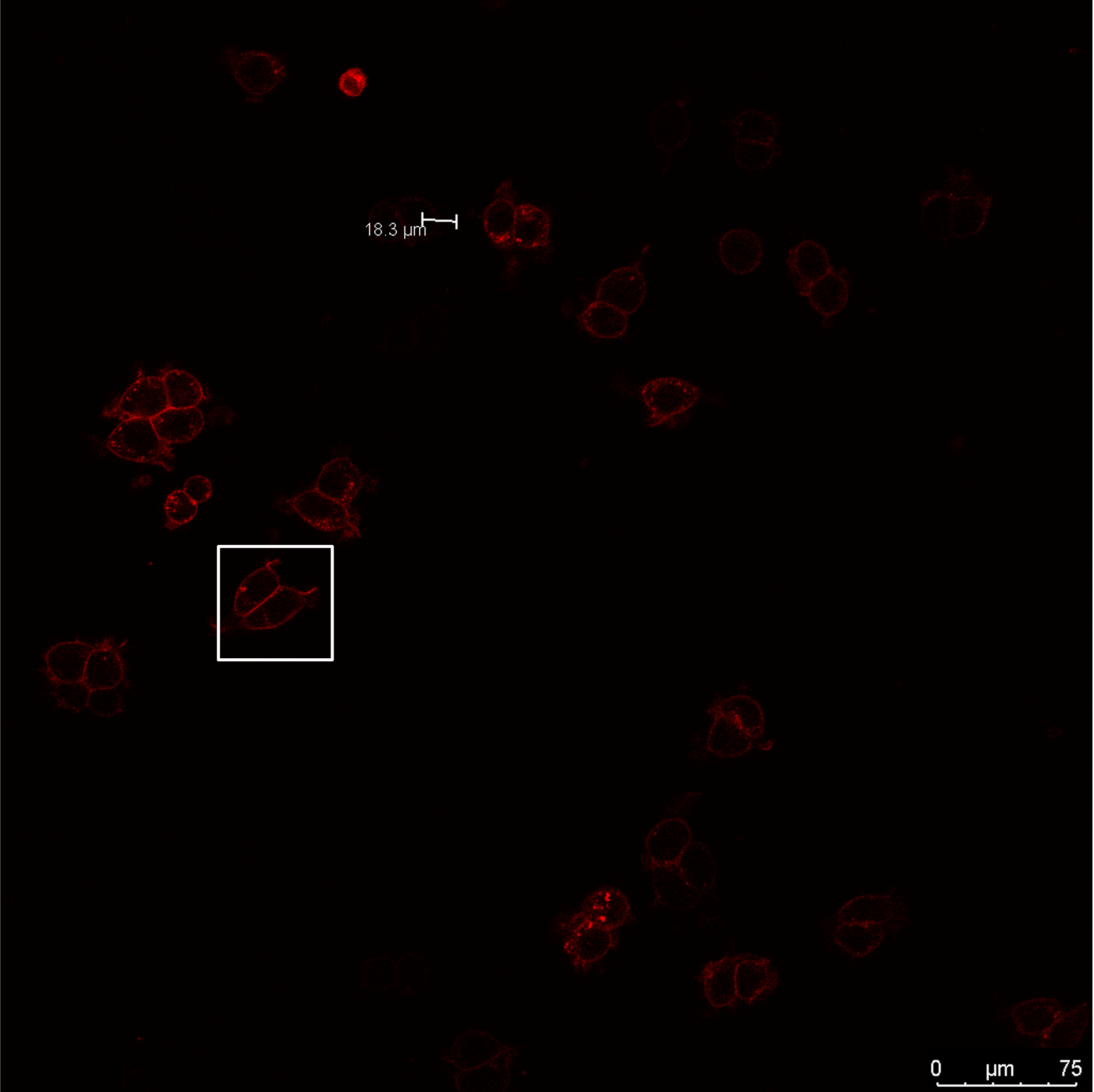

Supplement: Supplementary file 13 — Appendix and EV Figures Source Data [file 44319_2024_287_MOESM13_ESM.zip › FigureEV4A/Confocal image/WT/WT_ch01.png]

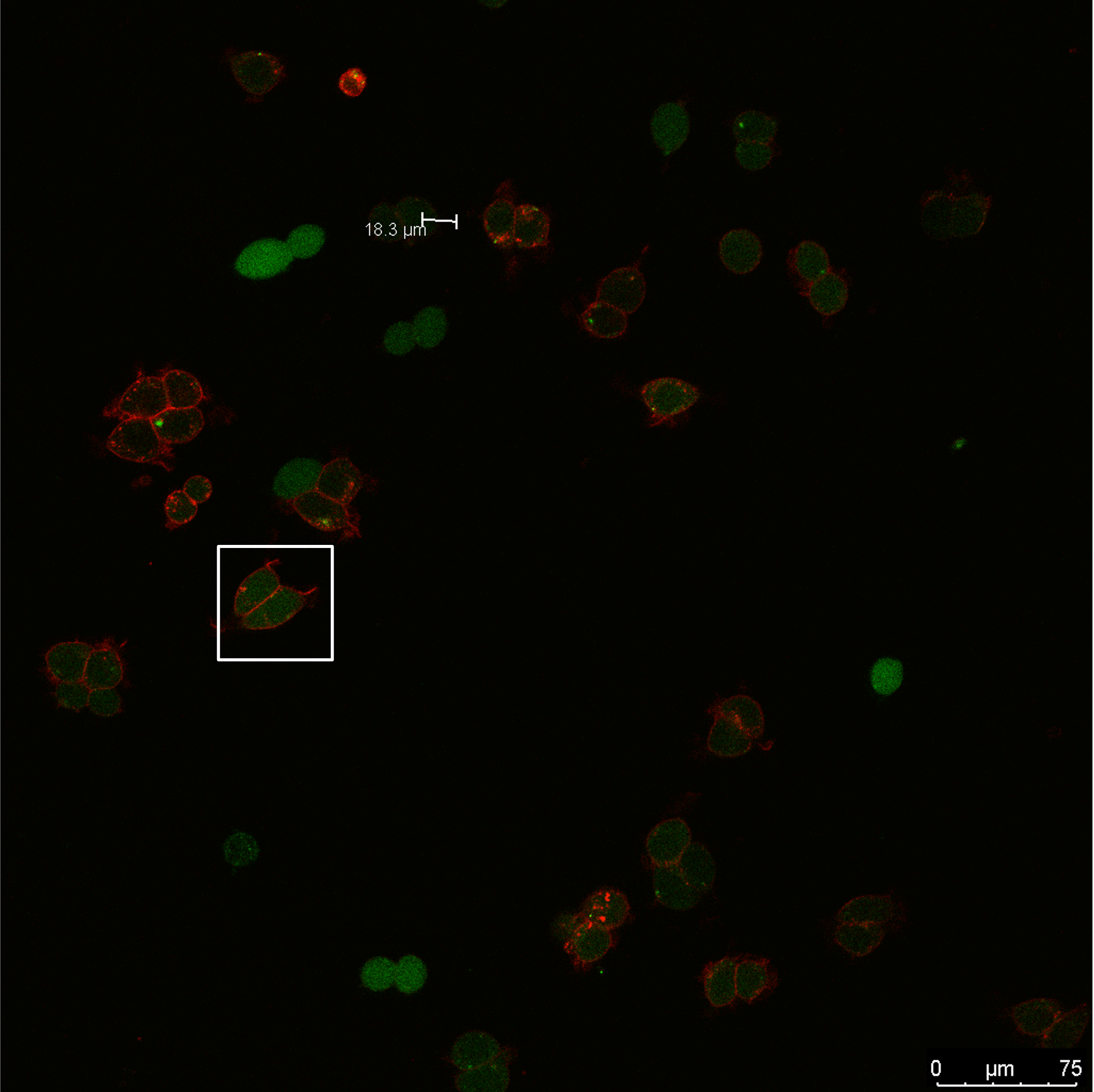

Supplement: Supplementary file 13 — Appendix and EV Figures Source Data [file 44319_2024_287_MOESM13_ESM.zip › FigureEV4A/Confocal image/WT/WT_merge.png]

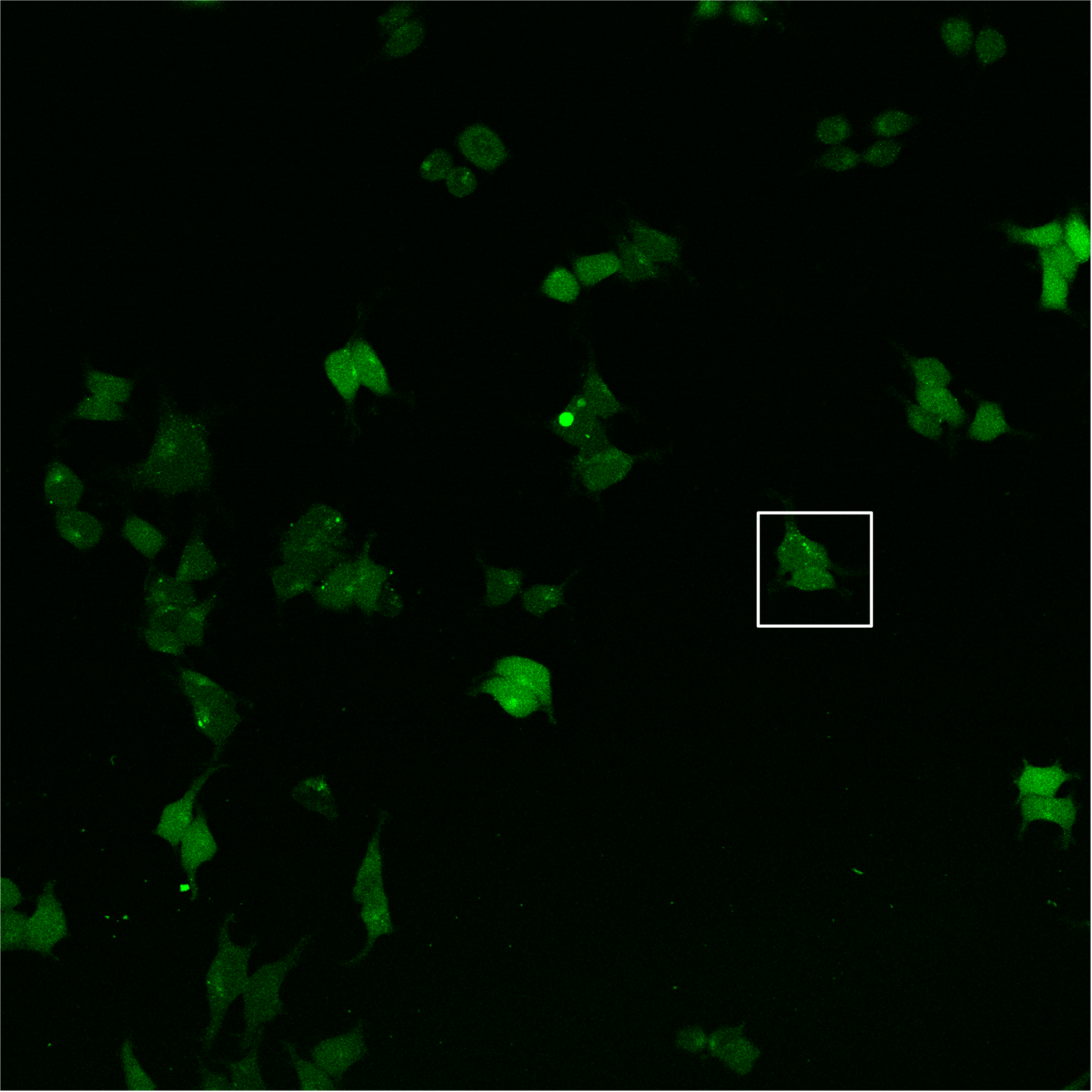

Supplement: Supplementary file 13 — Appendix and EV Figures Source Data [file 44319_2024_287_MOESM13_ESM.zip › FigureEV4A/Confocal image/Δ141-210/Δ141-210 _ch00.png]

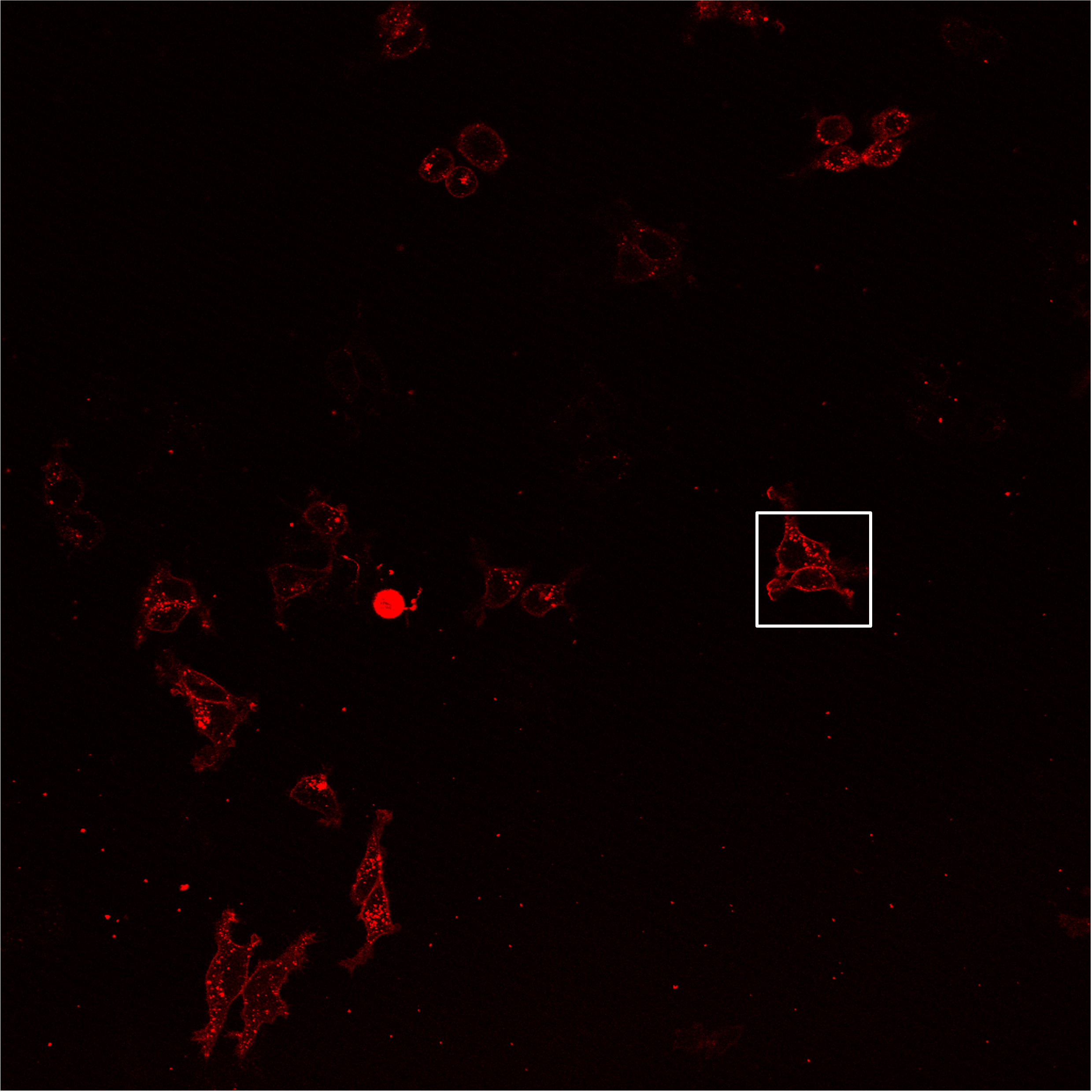

Supplement: Supplementary file 13 — Appendix and EV Figures Source Data [file 44319_2024_287_MOESM13_ESM.zip › FigureEV4A/Confocal image/Δ141-210/Δ141-210 _ch01.png]

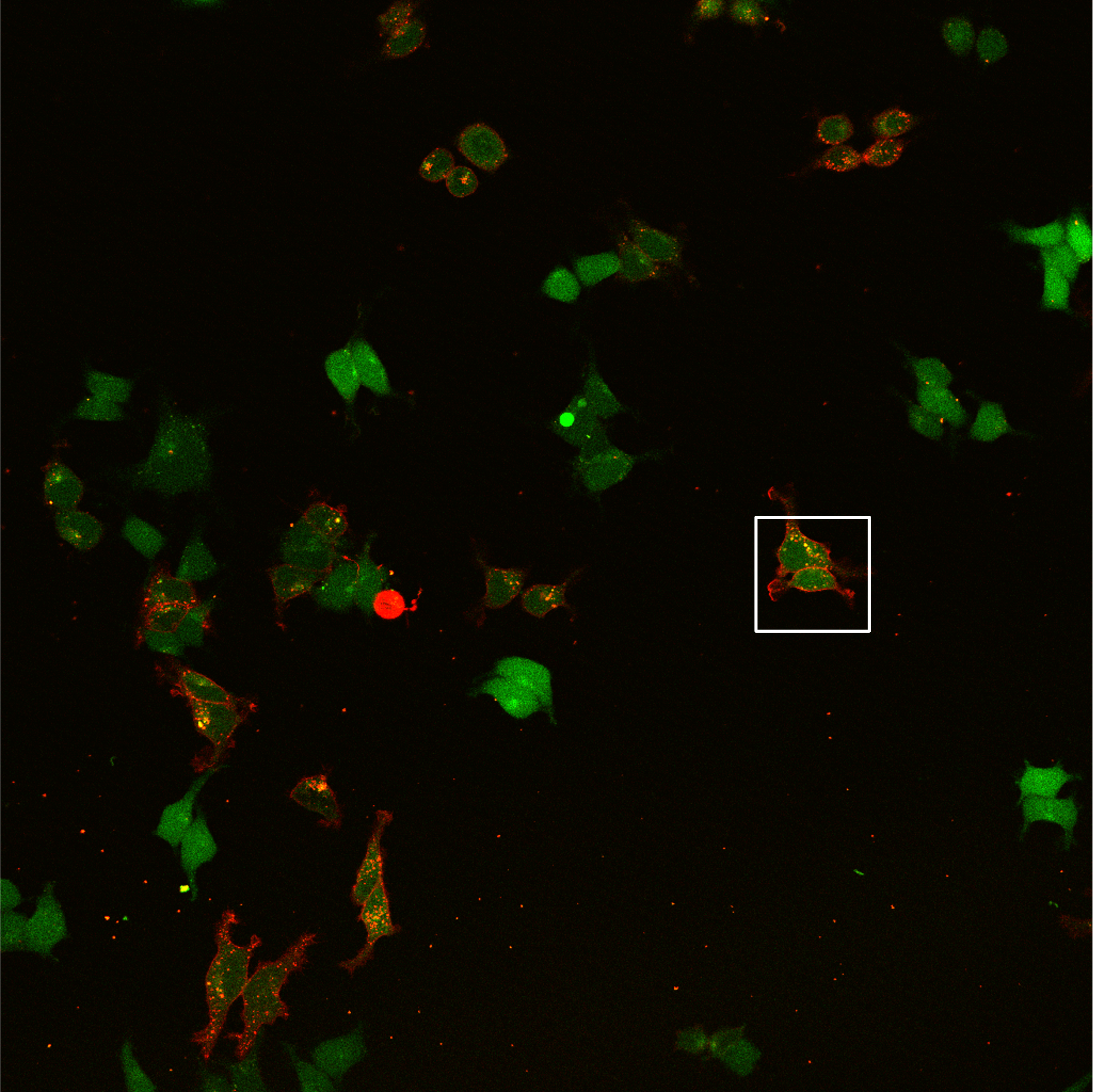

Supplement: Supplementary file 13 — Appendix and EV Figures Source Data [file 44319_2024_287_MOESM13_ESM.zip › FigureEV4A/Confocal image/Δ141-210/Δ141-210 _merge.png]

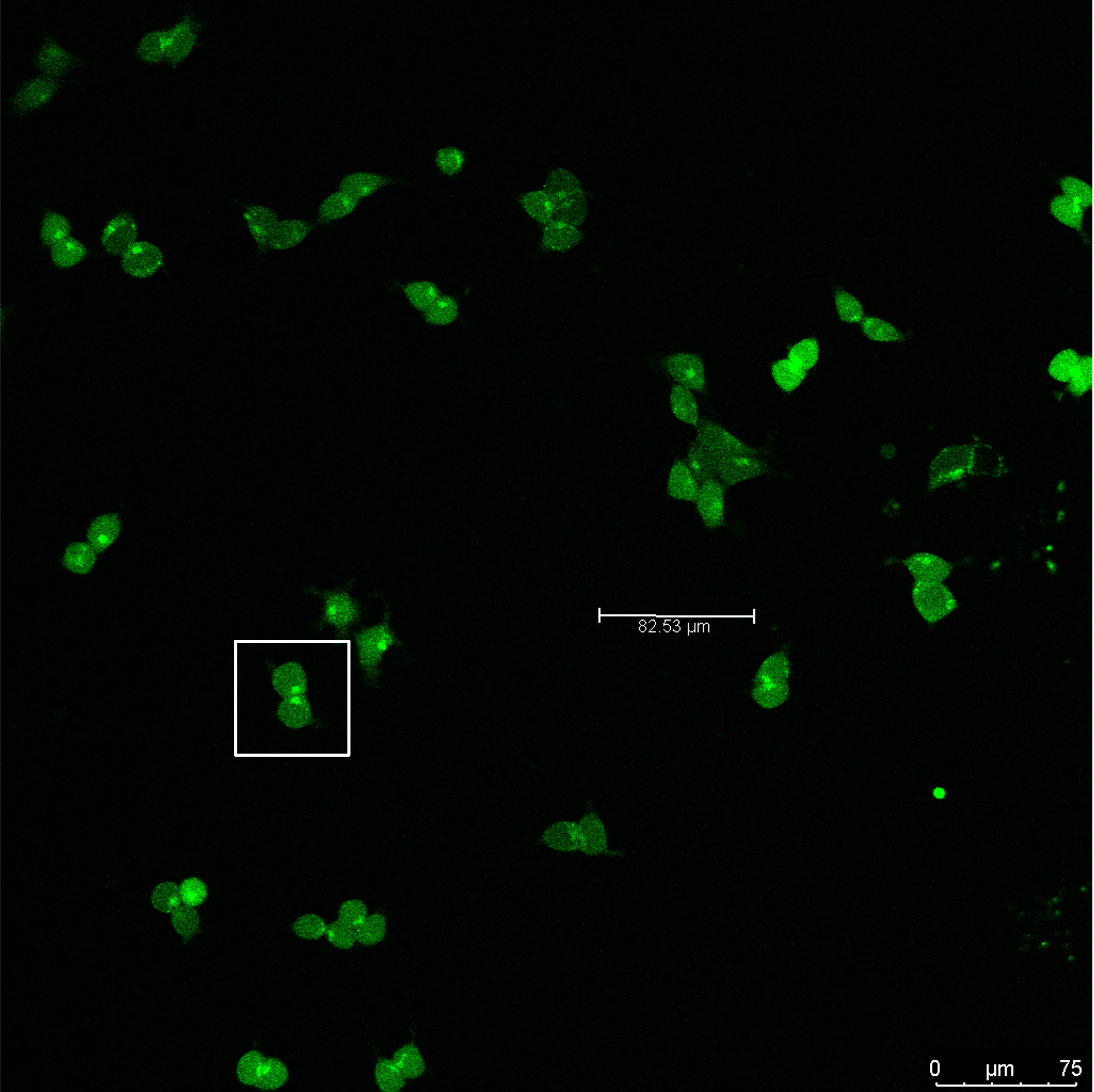

Supplement: Supplementary file 13 — Appendix and EV Figures Source Data [file 44319_2024_287_MOESM13_ESM.zip › FigureEV4A/Confocal image/Δ281-305/Δ281-305_ch00.png]

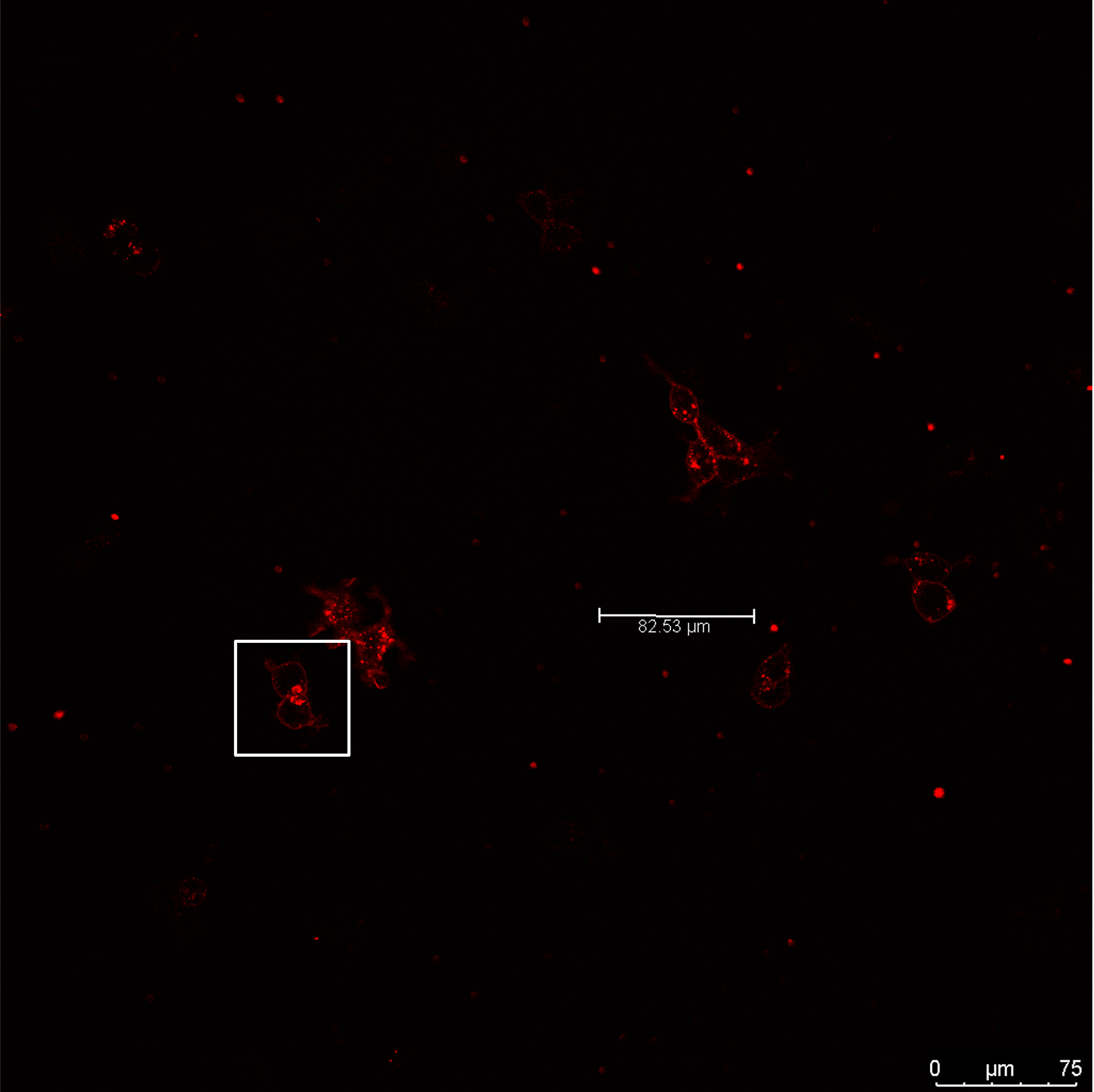

Supplement: Supplementary file 13 — Appendix and EV Figures Source Data [file 44319_2024_287_MOESM13_ESM.zip › FigureEV4A/Confocal image/Δ281-305/Δ281-305_ch01.png]

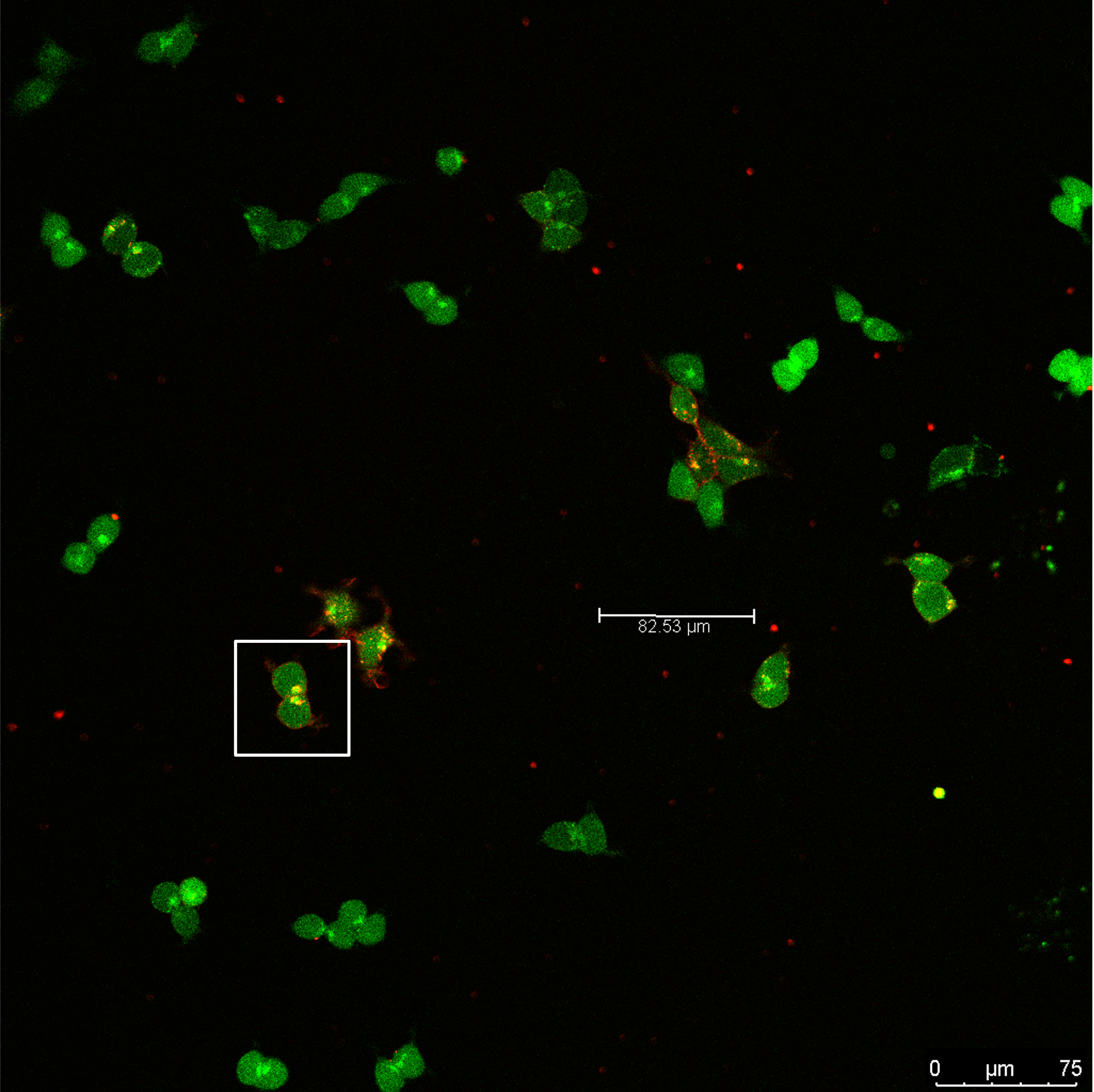

Supplement: Supplementary file 13 — Appendix and EV Figures Source Data [file 44319_2024_287_MOESM13_ESM.zip › FigureEV4A/Confocal image/Δ281-305/Δ281-305_merge.png]

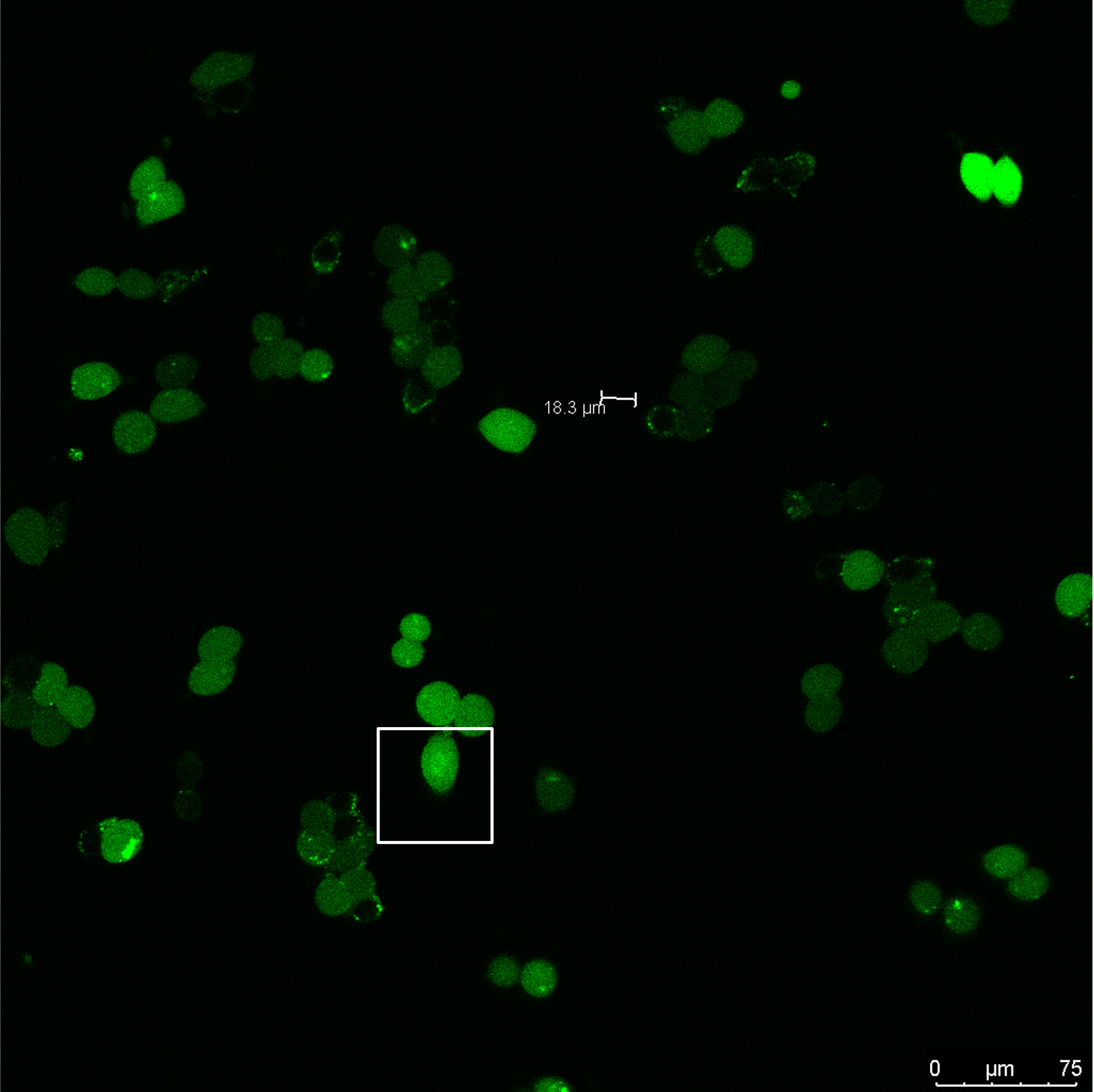

Supplement: Supplementary file 13 — Appendix and EV Figures Source Data [file 44319_2024_287_MOESM13_ESM.zip › FigureEV4A/Confocal image/Δ424-507/Δ424-507_ch0.png]

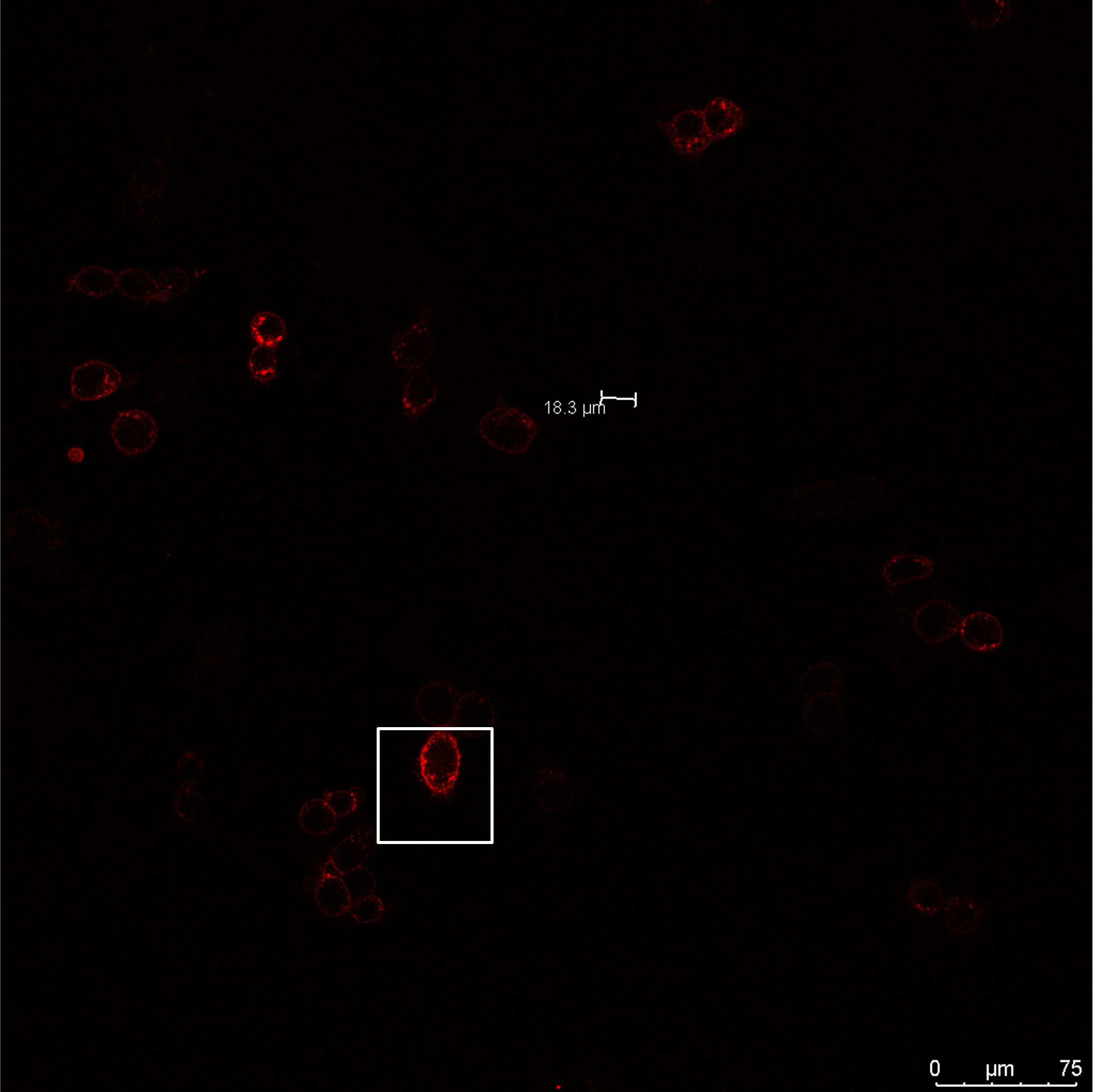

Supplement: Supplementary file 13 — Appendix and EV Figures Source Data [file 44319_2024_287_MOESM13_ESM.zip › FigureEV4A/Confocal image/Δ424-507/Δ424-507_ch01.png]

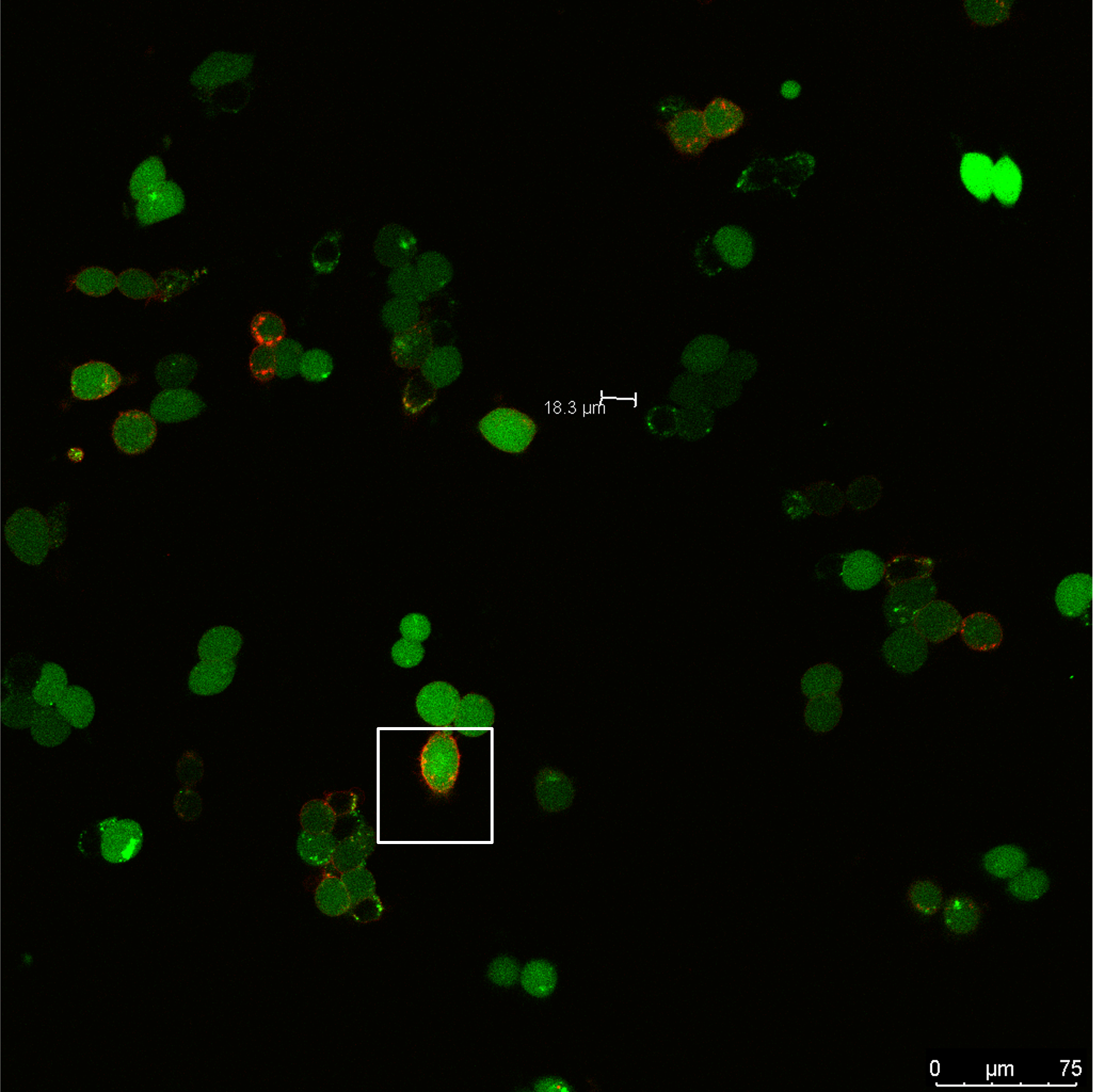

Supplement: Supplementary file 13 — Appendix and EV Figures Source Data [file 44319_2024_287_MOESM13_ESM.zip › FigureEV4A/Confocal image/Δ424-507/Δ424-507_merge.png]

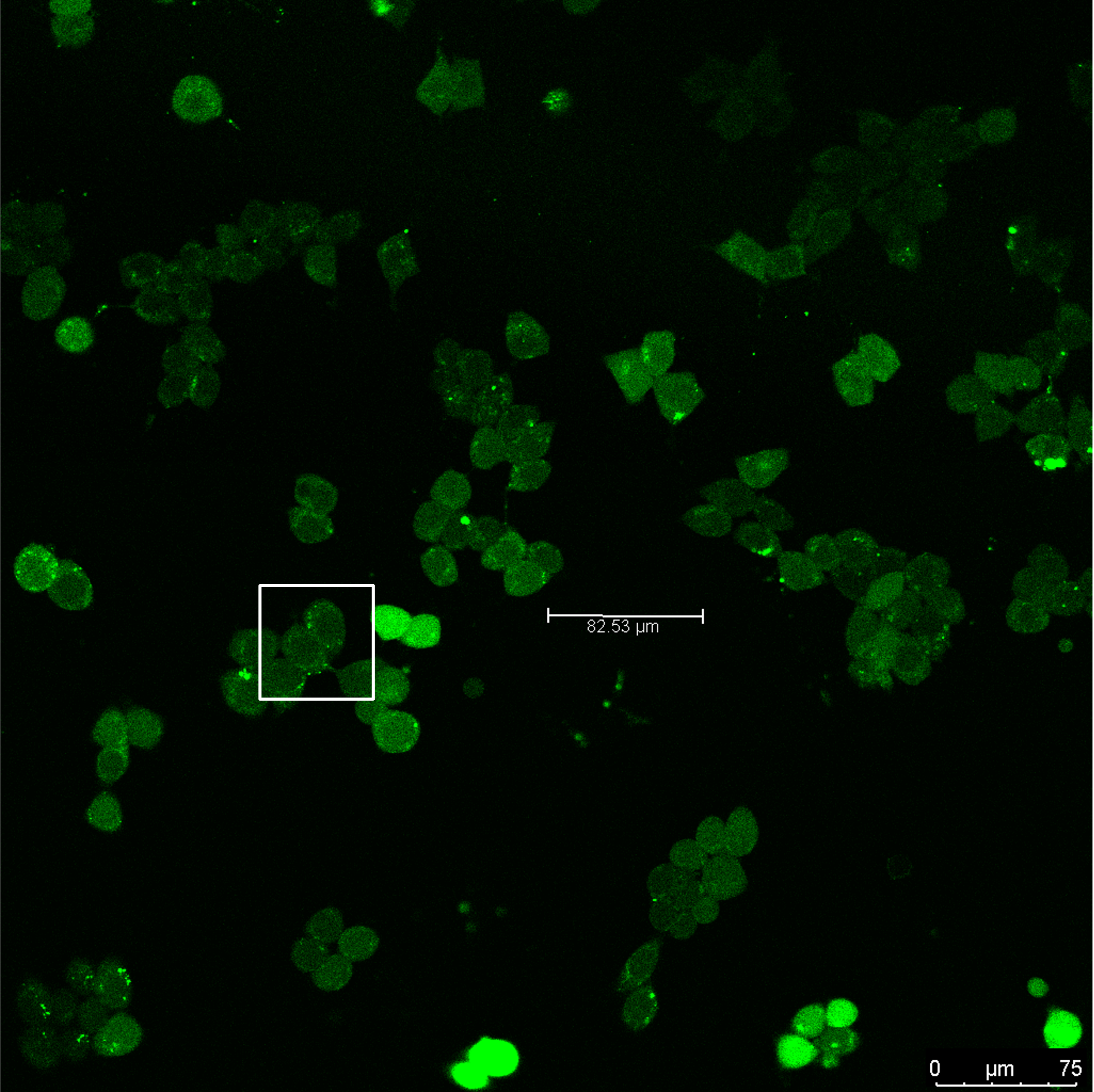

Supplement: Supplementary file 13 — Appendix and EV Figures Source Data [file 44319_2024_287_MOESM13_ESM.zip › FigureEV4A/Confocal image/Δ437-507/Δ437-507_ch00.png]

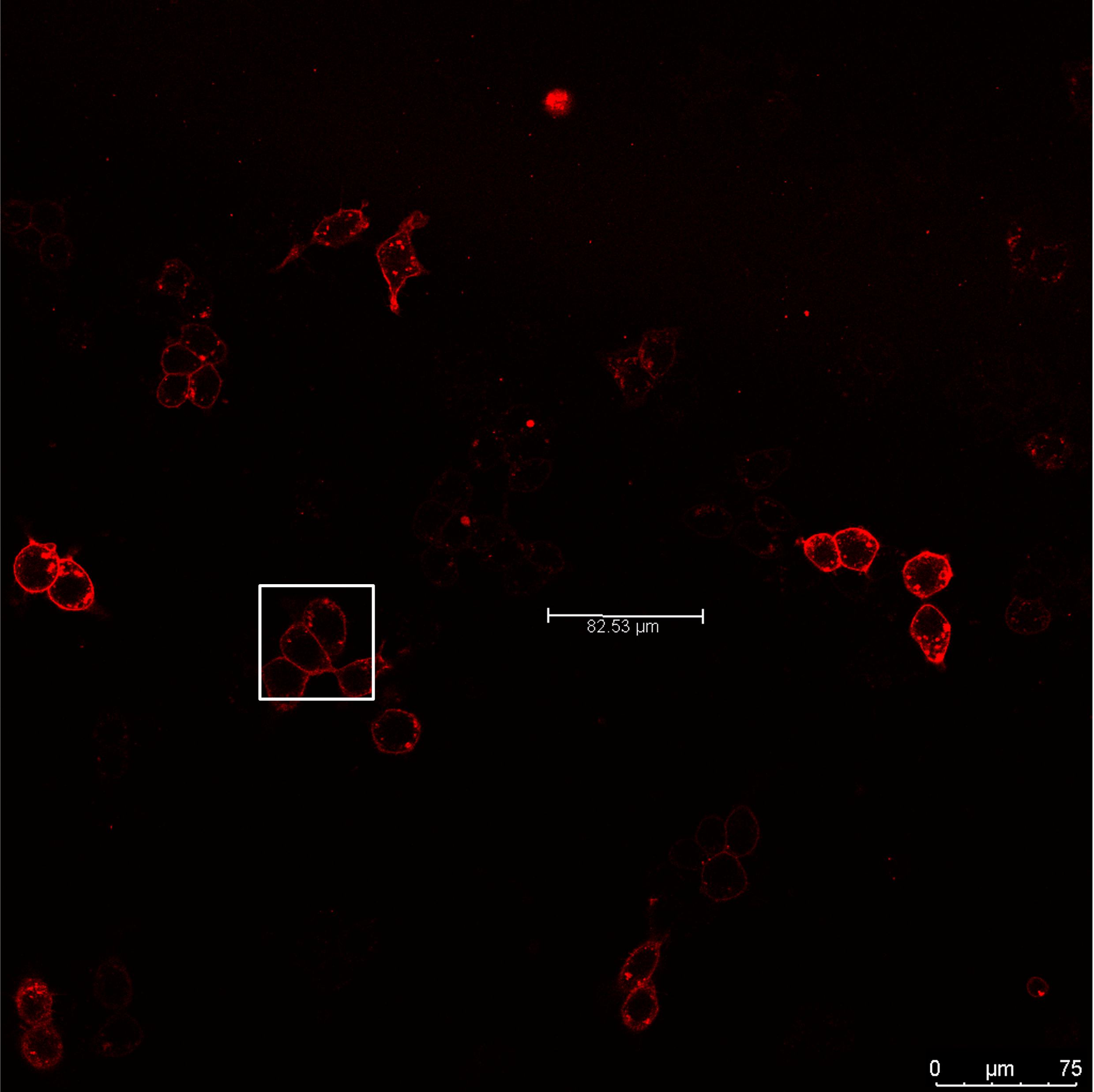

Supplement: Supplementary file 13 — Appendix and EV Figures Source Data [file 44319_2024_287_MOESM13_ESM.zip › FigureEV4A/Confocal image/Δ437-507/Δ437-507_ch01.png]

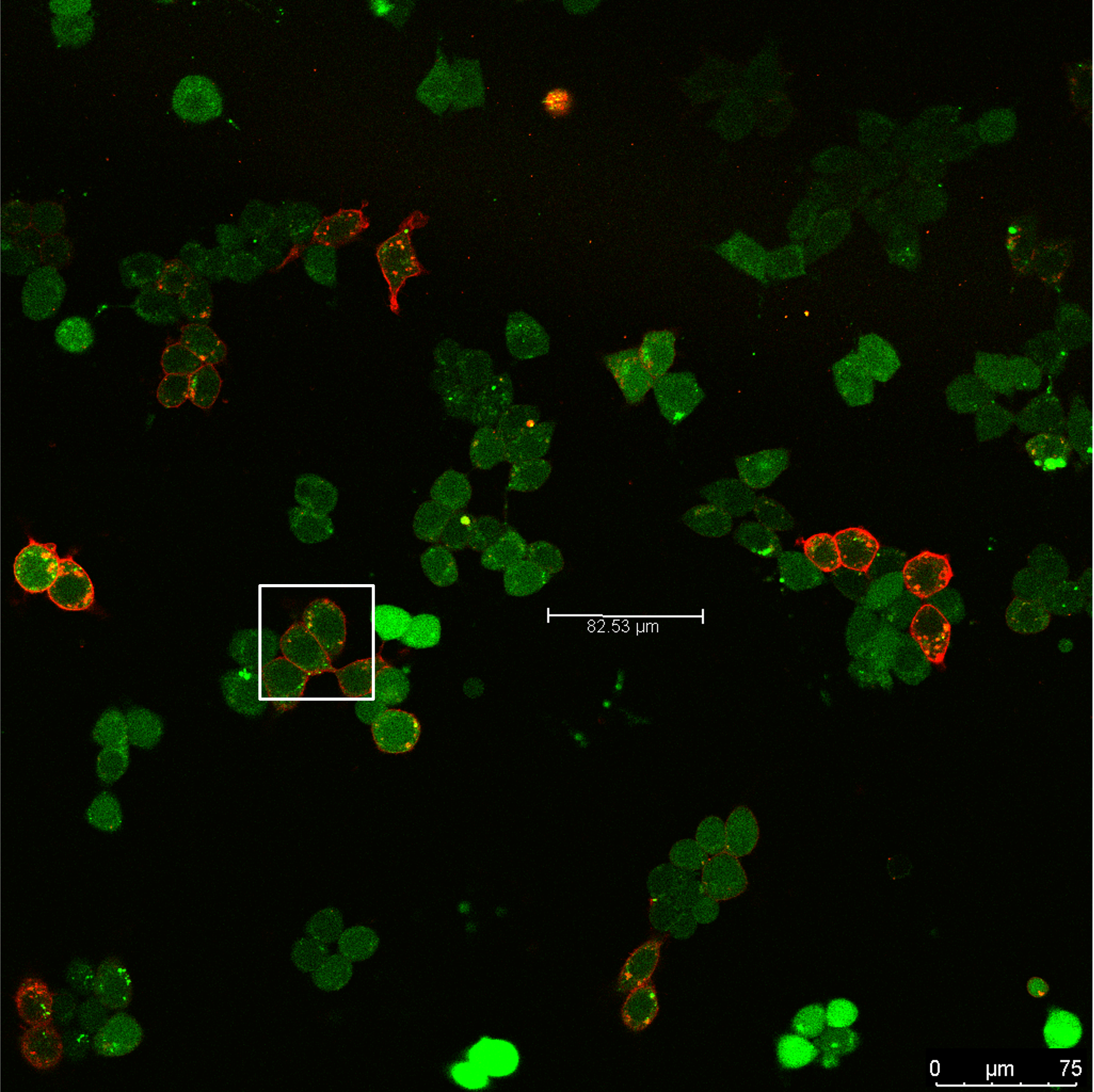

Supplement: Supplementary file 13 — Appendix and EV Figures Source Data [file 44319_2024_287_MOESM13_ESM.zip › FigureEV4A/Confocal image/Δ437-507/Δ437-507_merge.png]

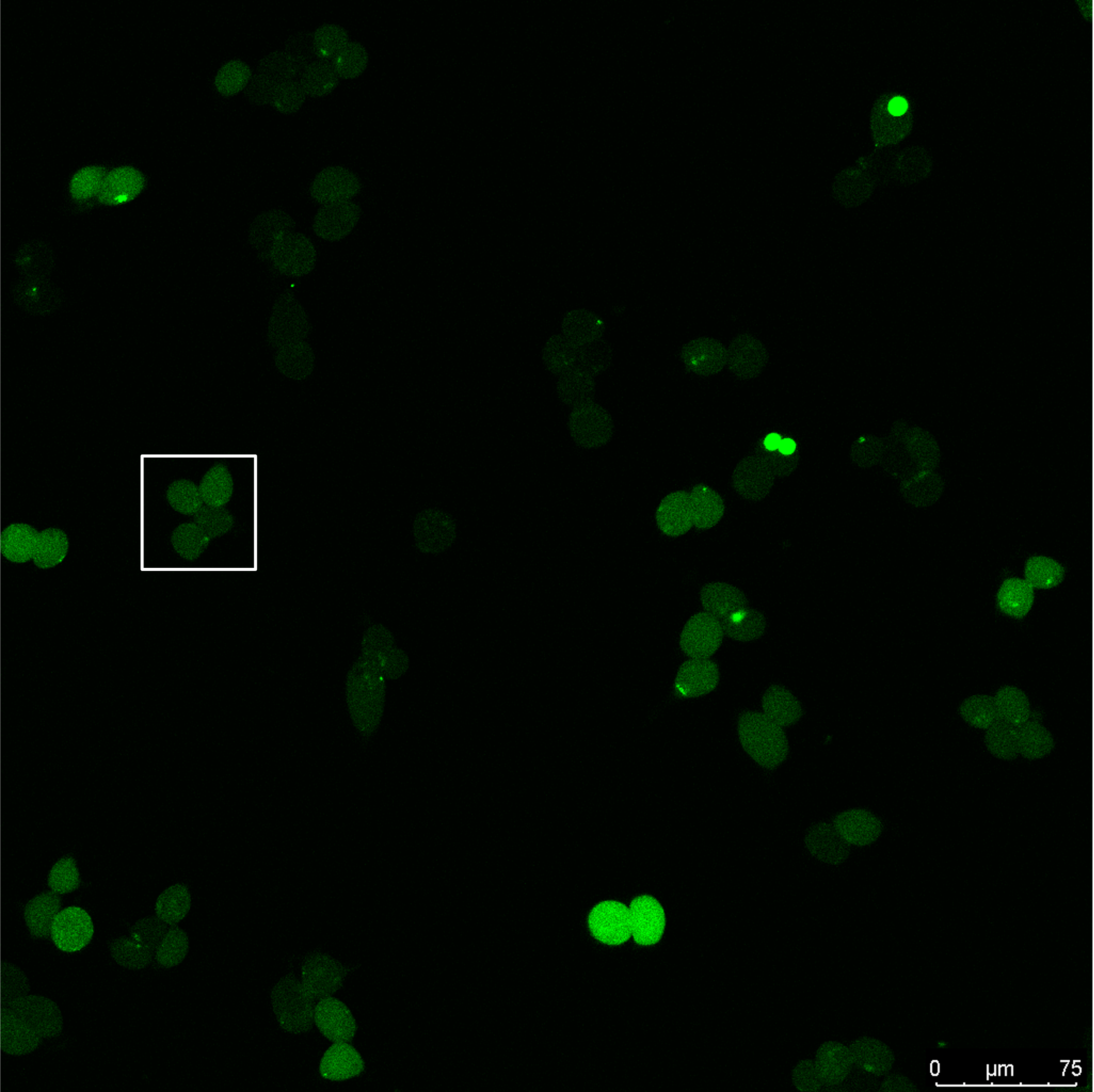

Supplement: Supplementary file 13 — Appendix and EV Figures Source Data [file 44319_2024_287_MOESM13_ESM.zip › FigureEV4A/Confocal image/Δ446-507/Δ446-507_ch00.png]

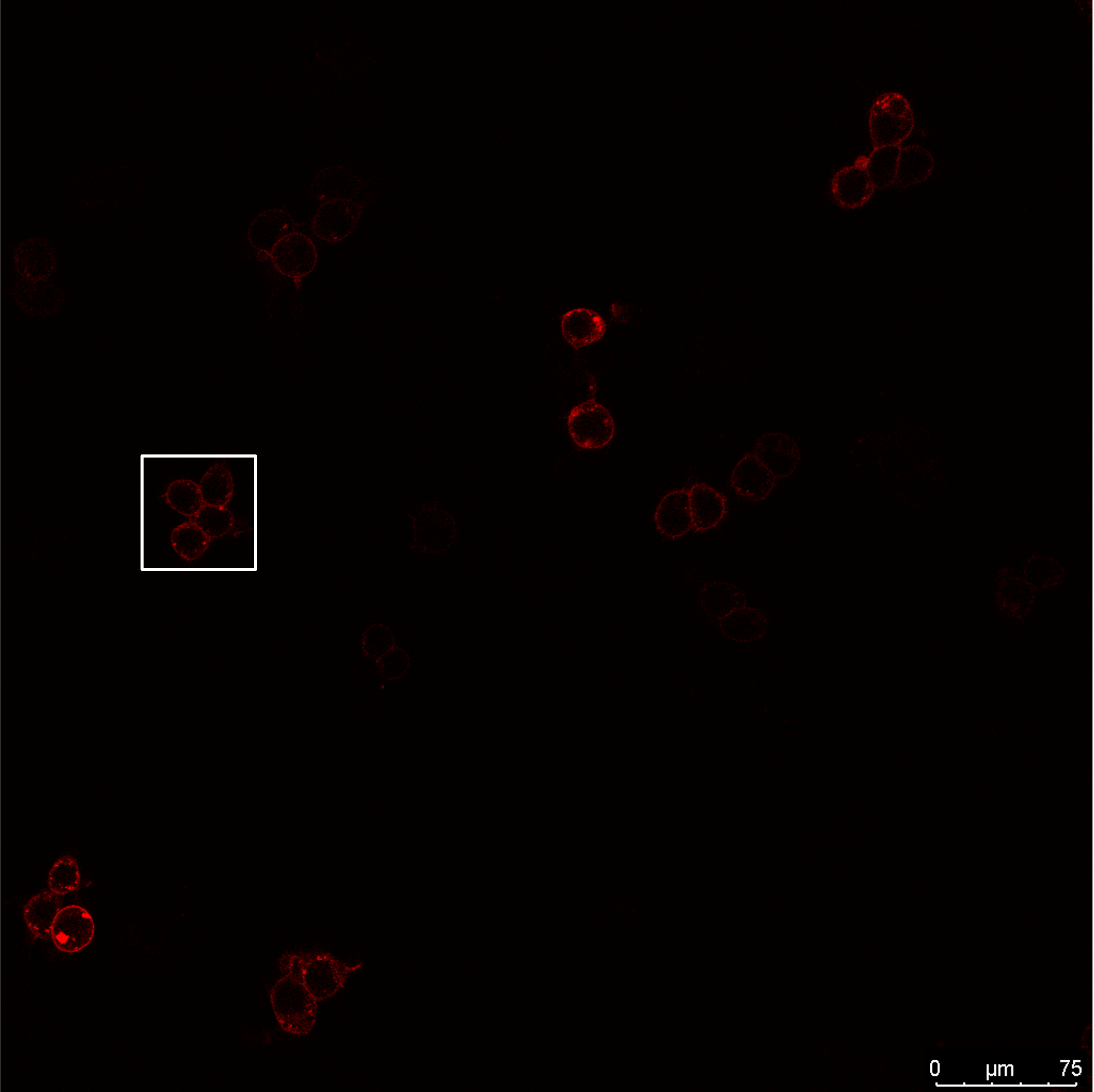

Supplement: Supplementary file 13 — Appendix and EV Figures Source Data [file 44319_2024_287_MOESM13_ESM.zip › FigureEV4A/Confocal image/Δ446-507/Δ446-507_ch01.png]

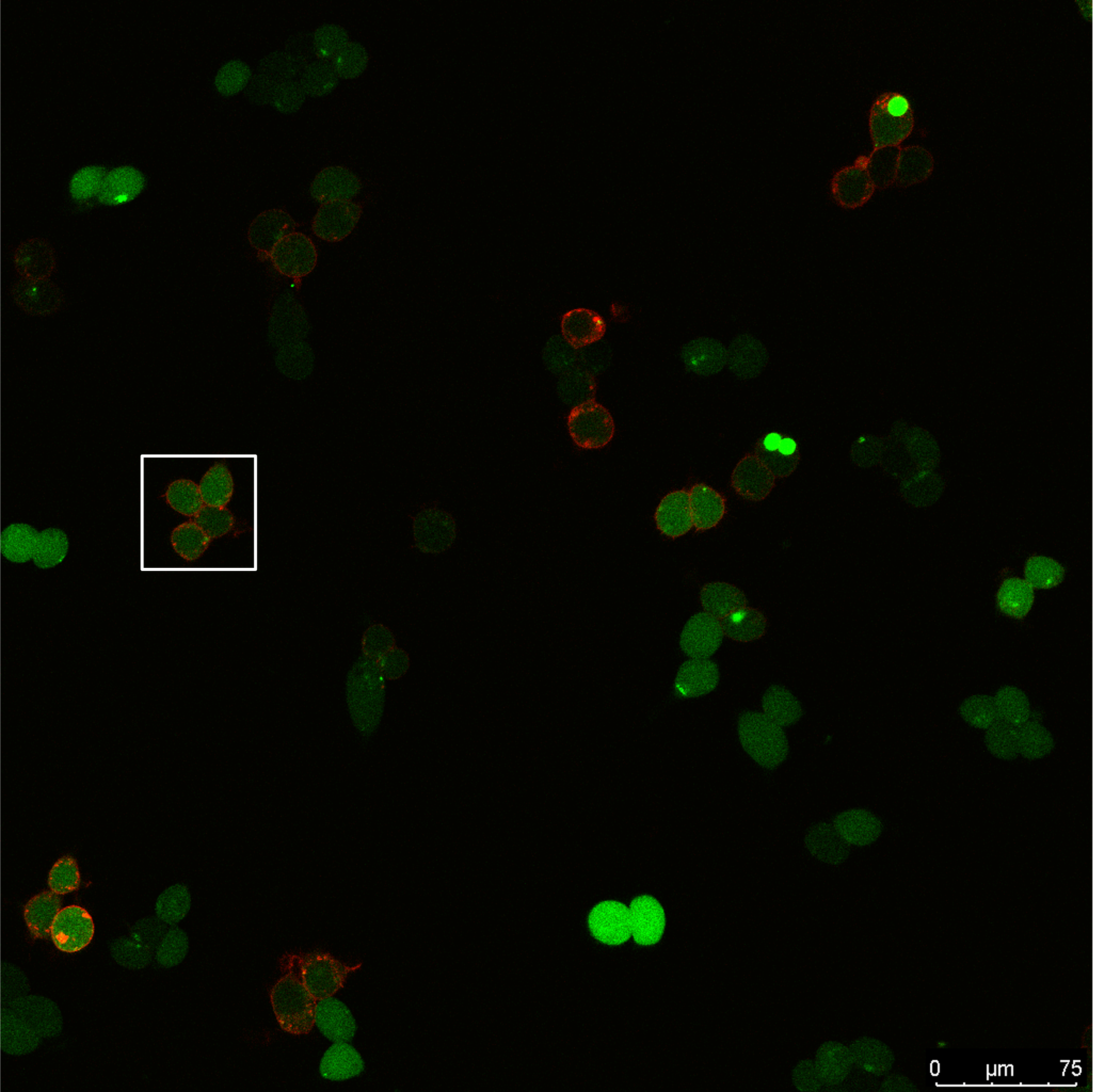

Supplement: Supplementary file 13 — Appendix and EV Figures Source Data [file 44319_2024_287_MOESM13_ESM.zip › FigureEV4A/Confocal image/Δ446-507/Δ446-507_merge.png]

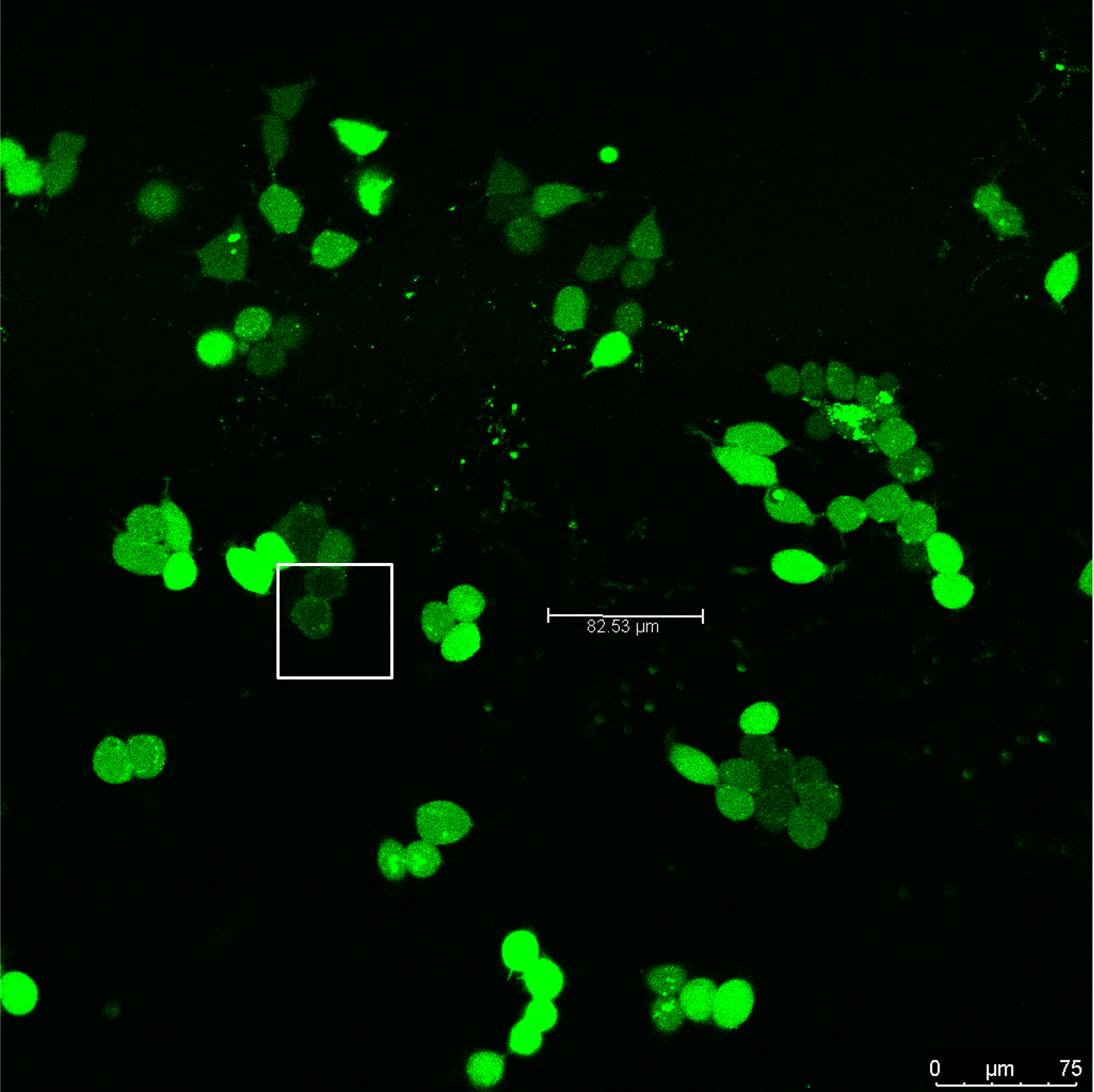

Supplement: Supplementary file 13 — Appendix and EV Figures Source Data [file 44319_2024_287_MOESM13_ESM.zip › FigureEV4A/Confocal image/Δ451-507/Δ451-507_ch00.png]

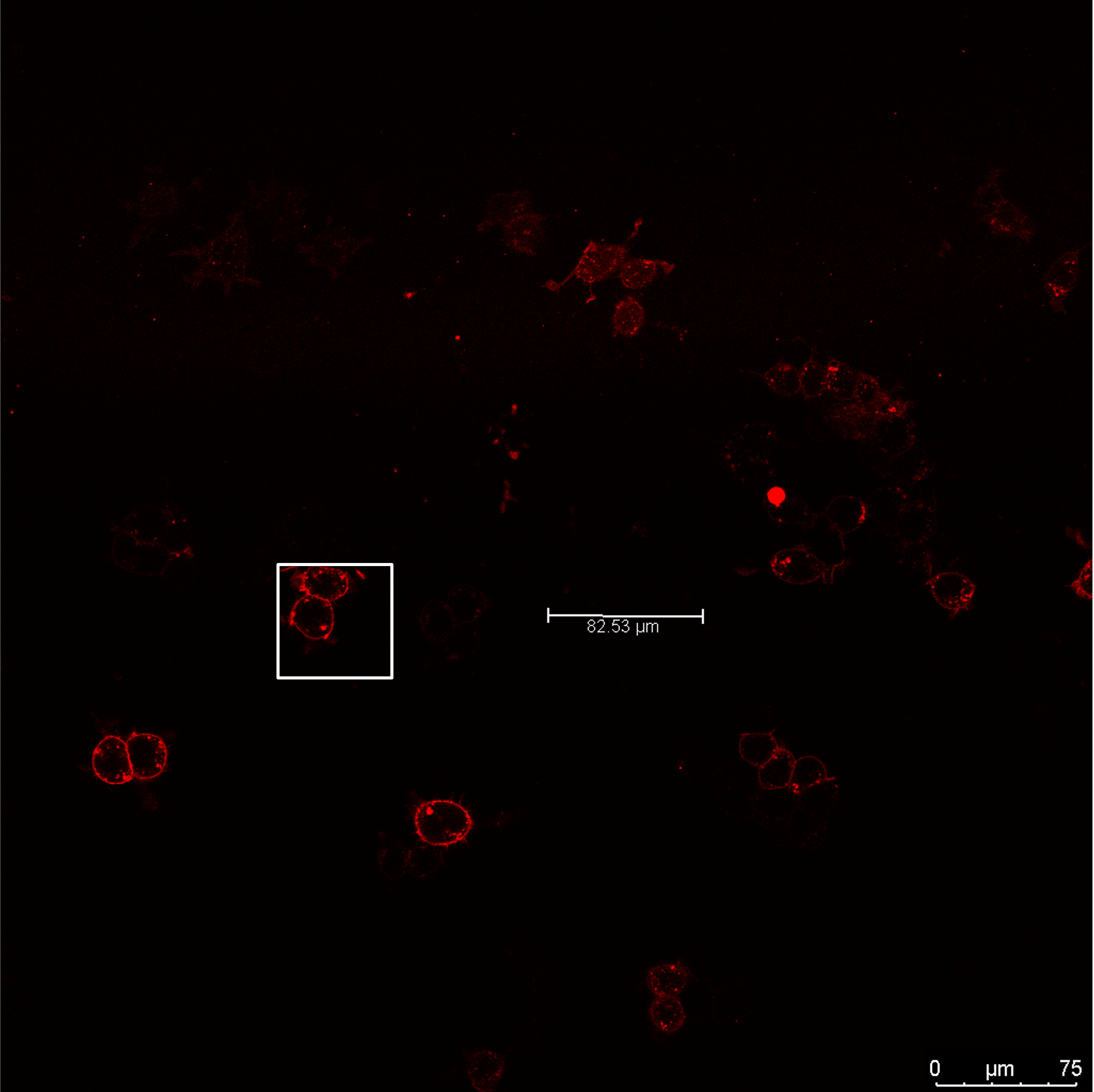

Supplement: Supplementary file 13 — Appendix and EV Figures Source Data [file 44319_2024_287_MOESM13_ESM.zip › FigureEV4A/Confocal image/Δ451-507/Δ451-507_ch01.png]

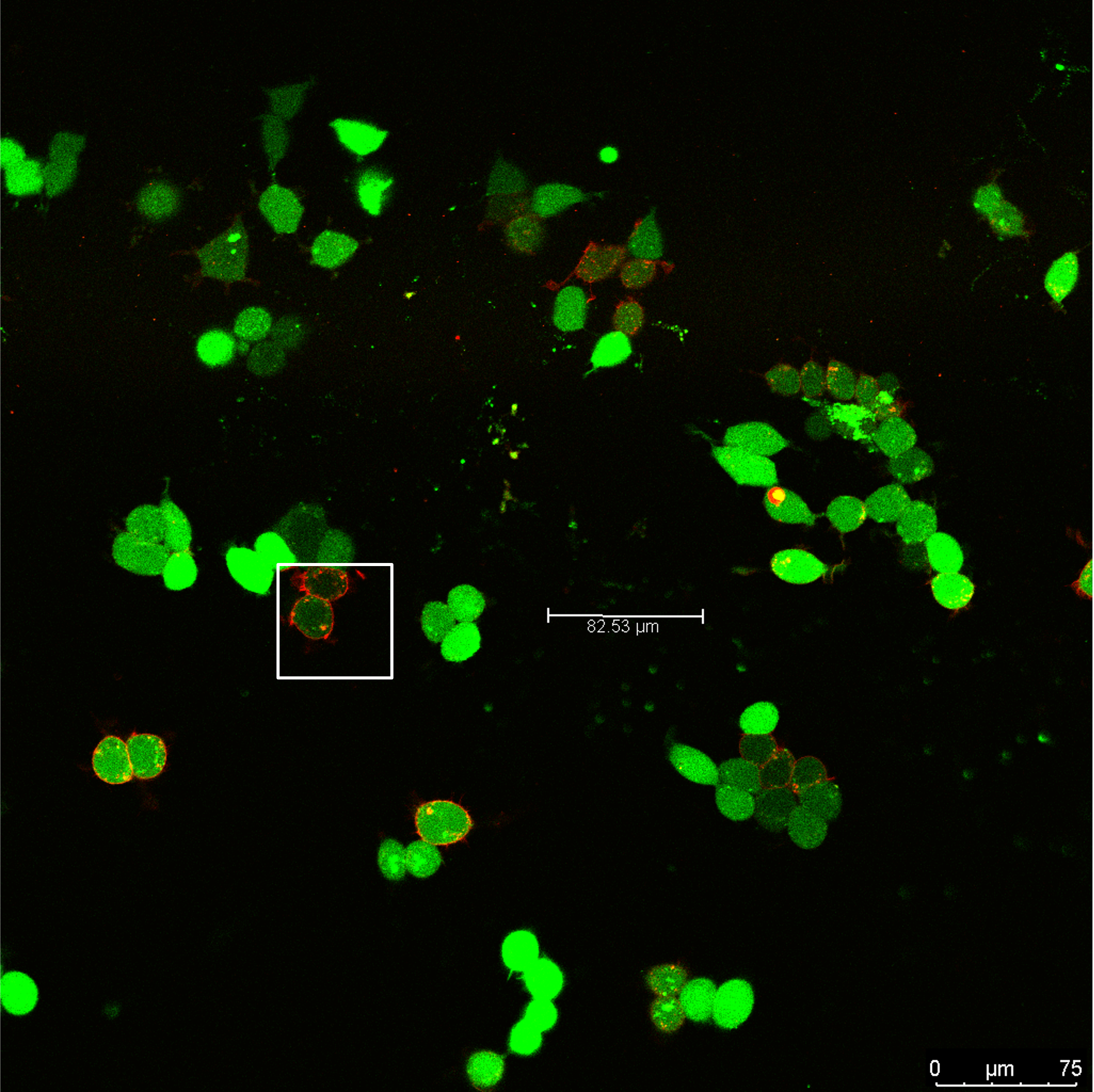

Supplement: Supplementary file 13 — Appendix and EV Figures Source Data [file 44319_2024_287_MOESM13_ESM.zip › FigureEV4A/Confocal image/Δ451-507/Δ451-507_merge.png]

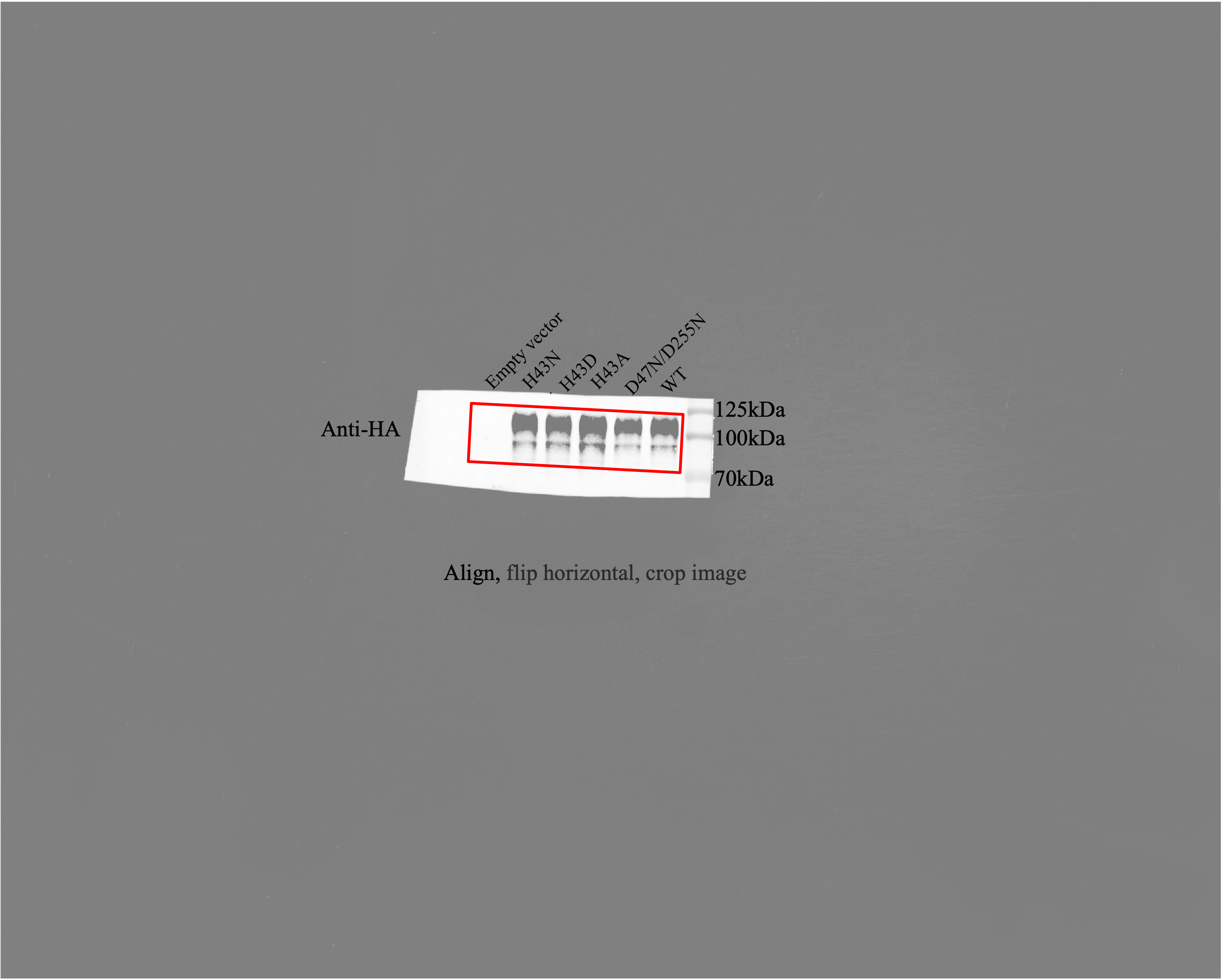

Supplement: Supplementary file 13 — Appendix and EV Figures Source Data [file 44319_2024_287_MOESM13_ESM.zip › FigureEV4A/WB-1/HA cropping area.png]

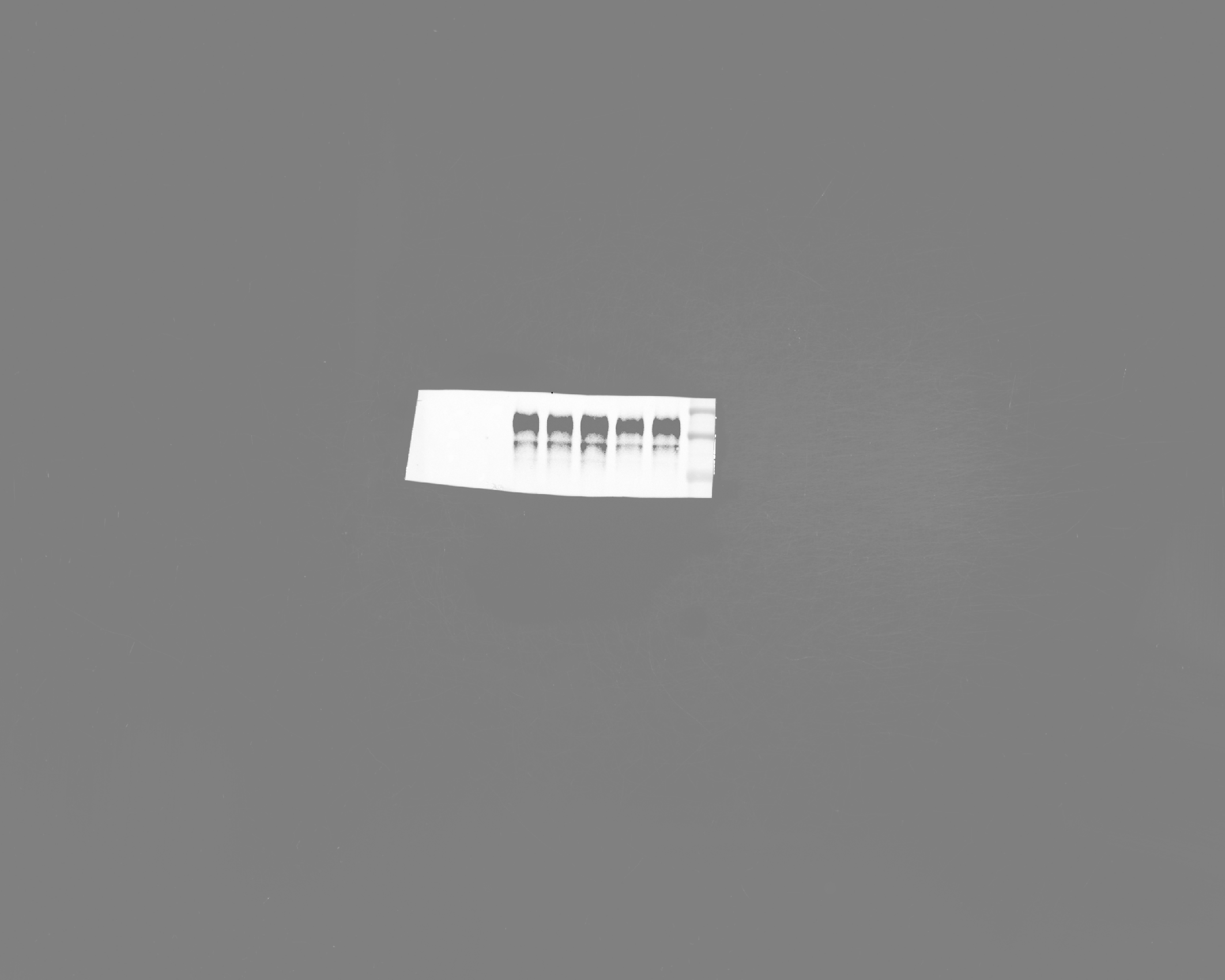

Supplement: Supplementary file 13 — Appendix and EV Figures Source Data [file 44319_2024_287_MOESM13_ESM.zip › FigureEV4A/WB-1/HA(Composite).tif]

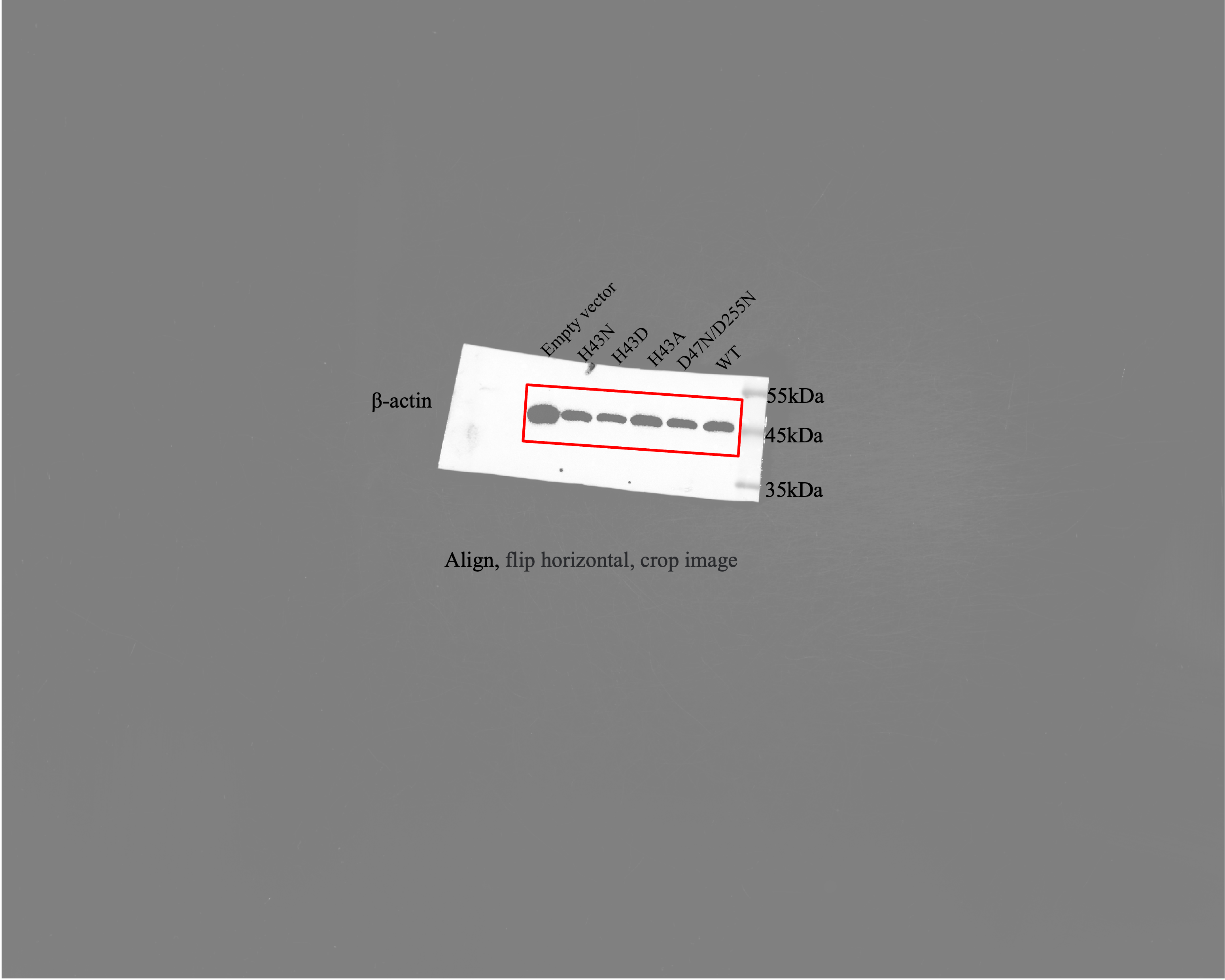

Supplement: Supplementary file 13 — Appendix and EV Figures Source Data [file 44319_2024_287_MOESM13_ESM.zip › FigureEV4A/WB-1/β-actin cropping area.png]

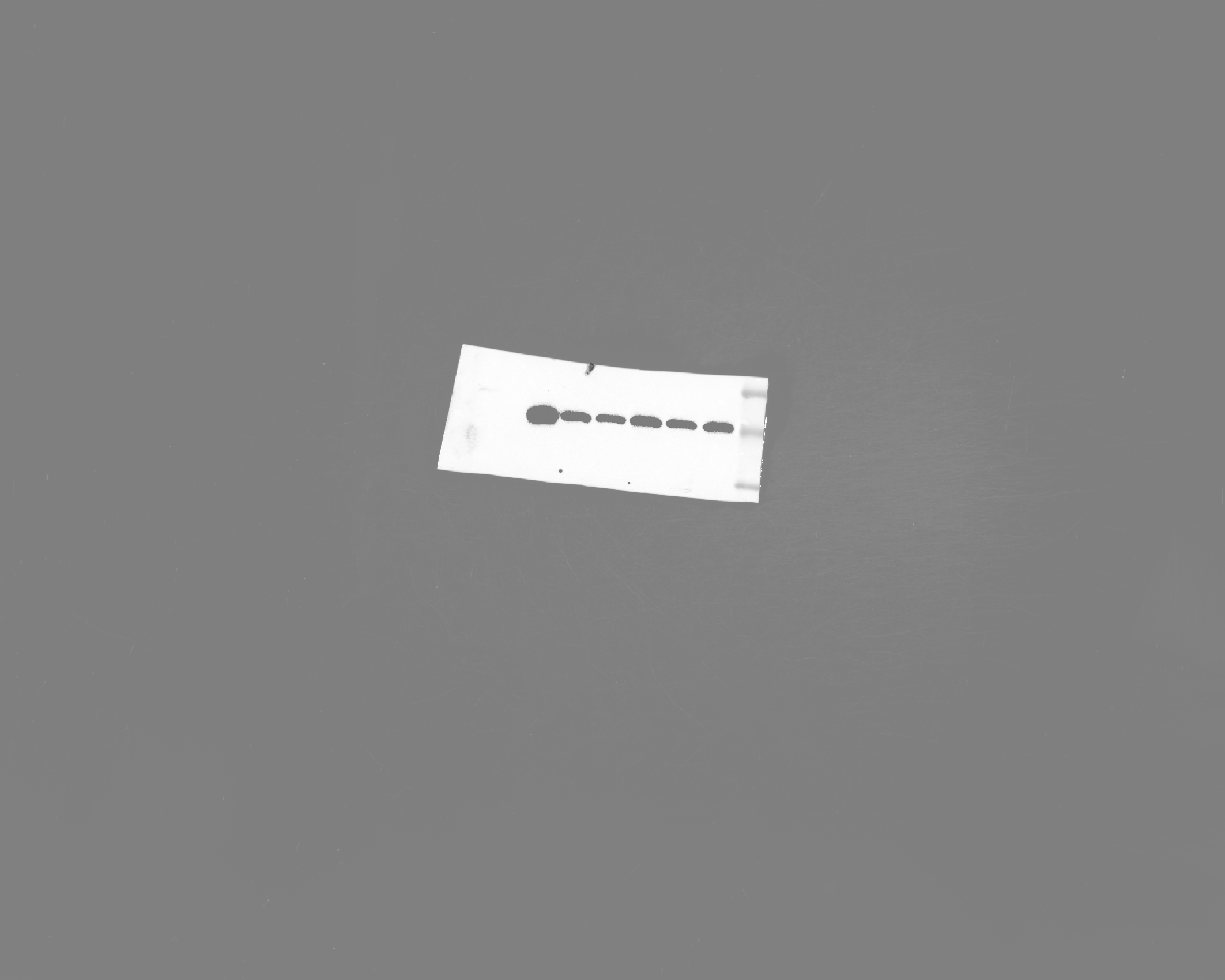

Supplement: Supplementary file 13 — Appendix and EV Figures Source Data [file 44319_2024_287_MOESM13_ESM.zip › FigureEV4A/WB-1/β-actin(Composite).tif]

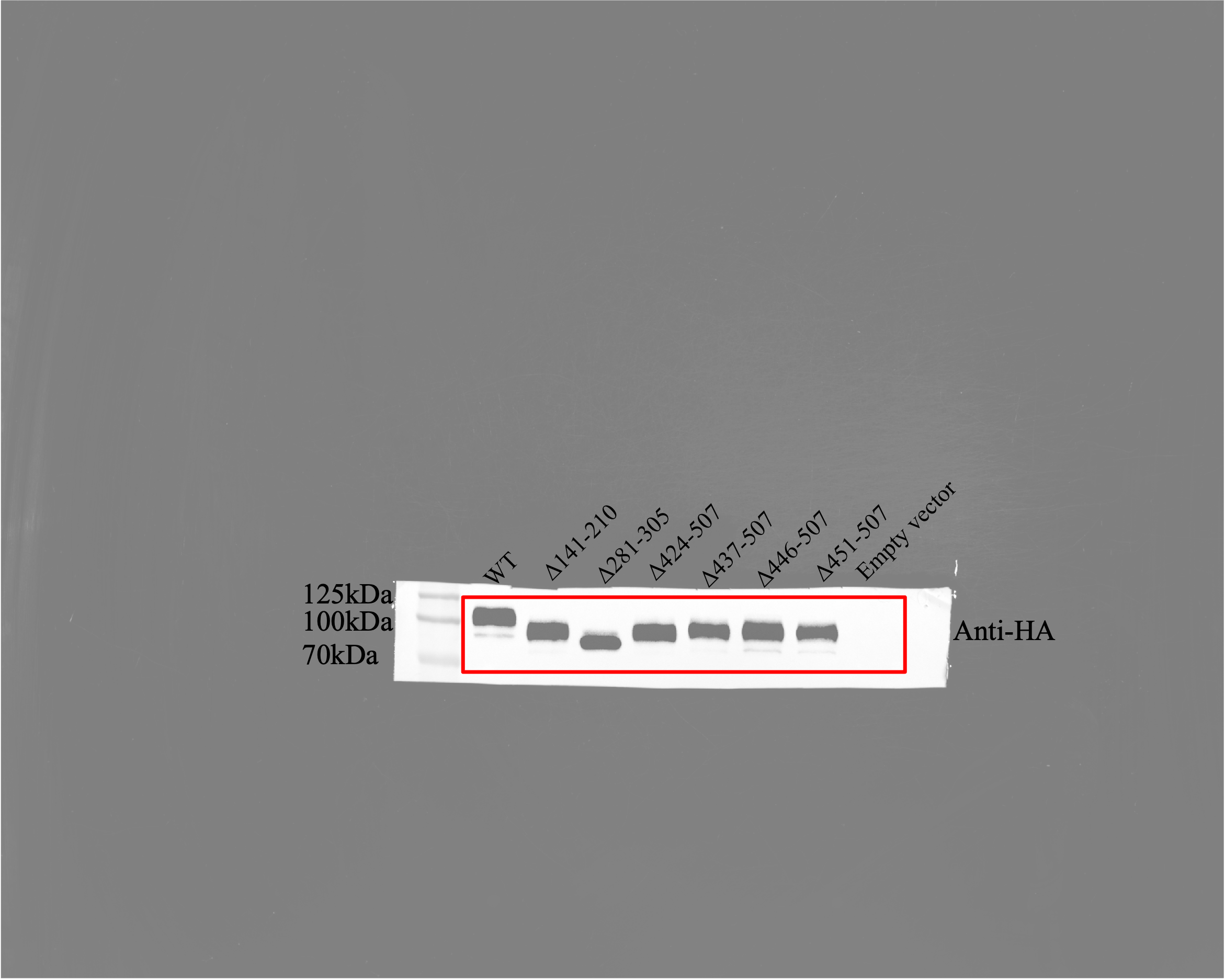

Supplement: Supplementary file 13 — Appendix and EV Figures Source Data [file 44319_2024_287_MOESM13_ESM.zip › FigureEV4A/WB-2/Anti-HA cropping area.png]

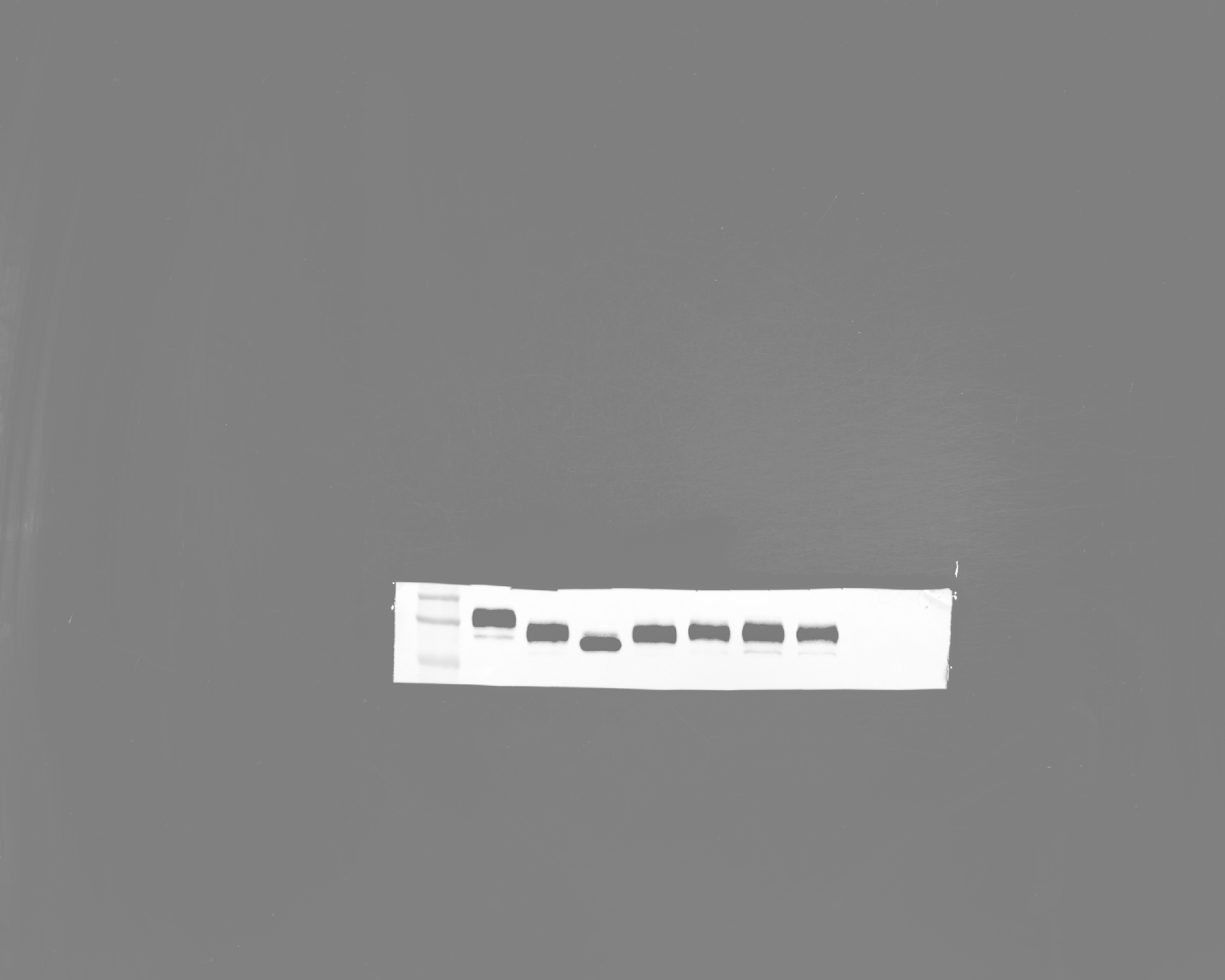

Supplement: Supplementary file 13 — Appendix and EV Figures Source Data [file 44319_2024_287_MOESM13_ESM.zip › FigureEV4A/WB-2/Anti-HA.tif]

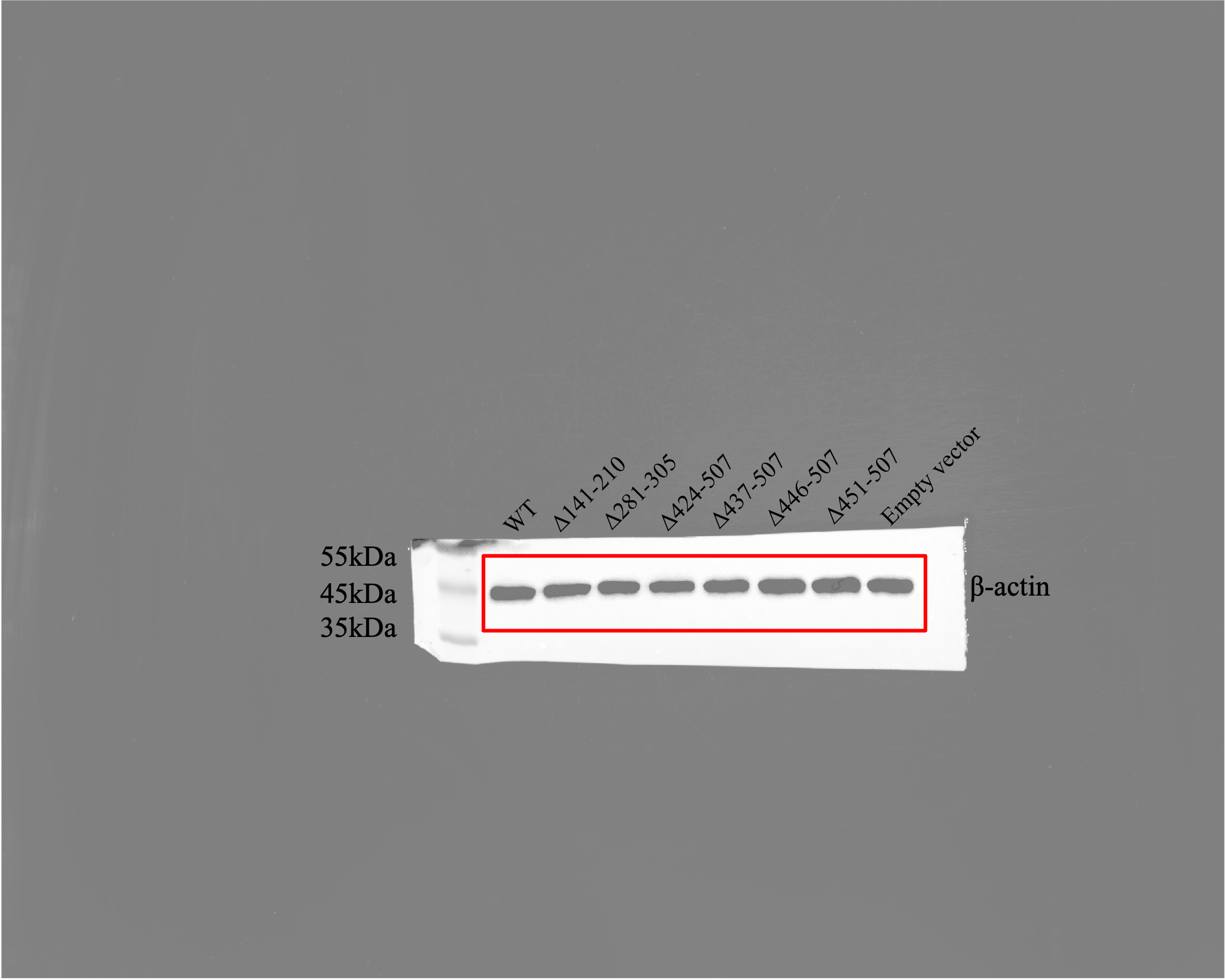

Supplement: Supplementary file 13 — Appendix and EV Figures Source Data [file 44319_2024_287_MOESM13_ESM.zip › FigureEV4A/WB-2/β-actin cropping area.png]

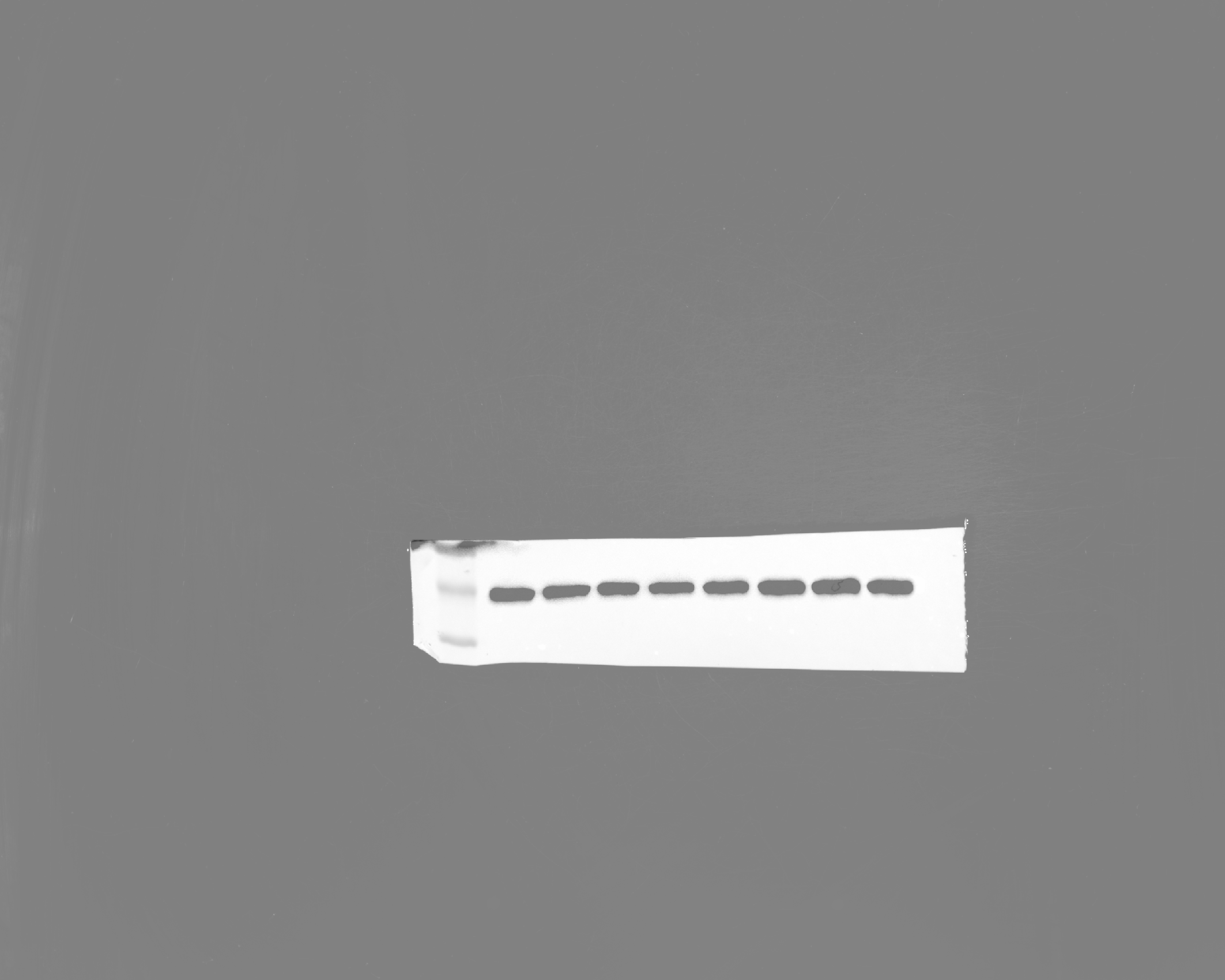

Supplement: Supplementary file 13 — Appendix and EV Figures Source Data [file 44319_2024_287_MOESM13_ESM.zip › FigureEV4A/WB-2/β-actin.tif]

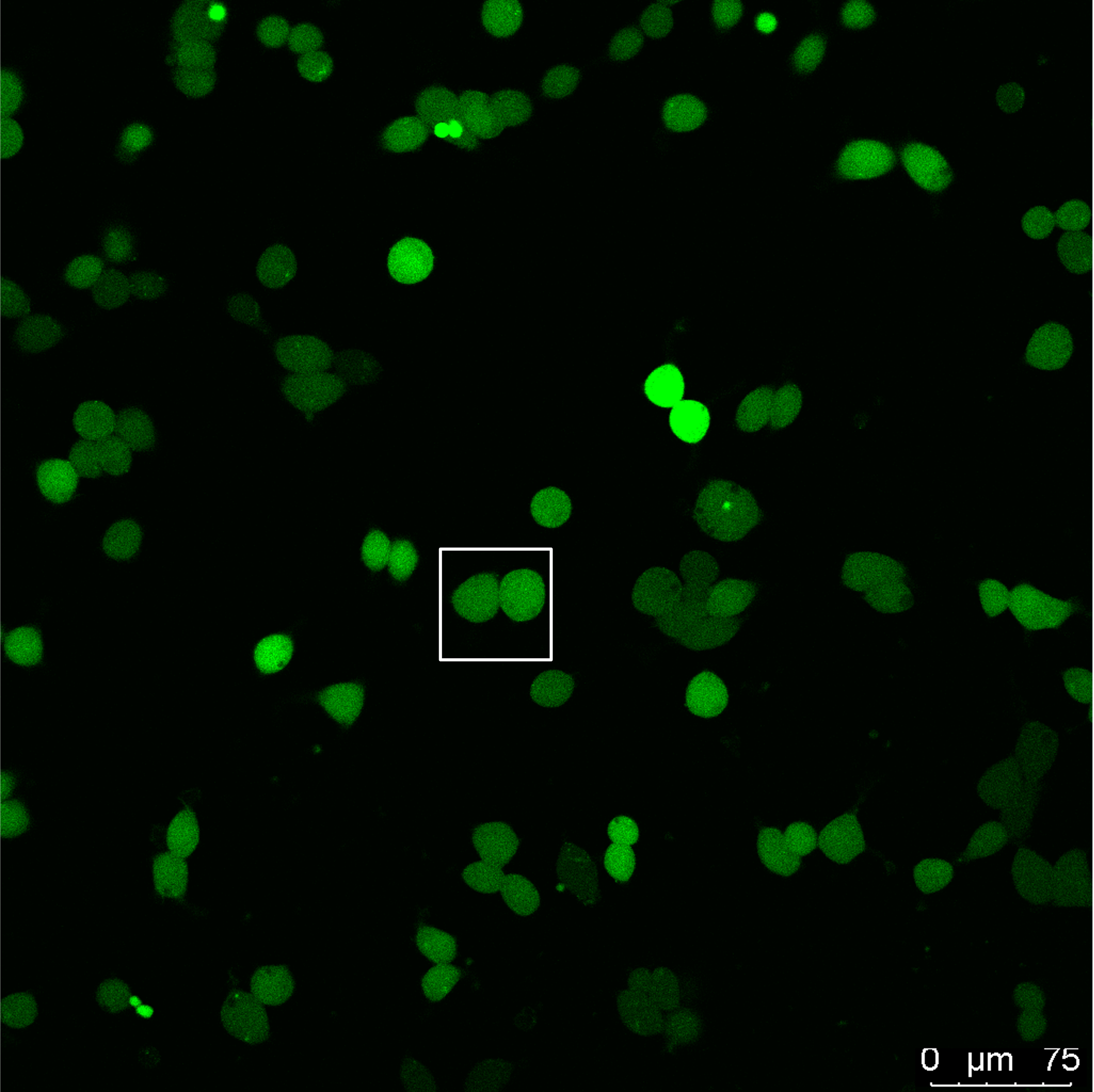

Supplement: Supplementary file 13 — Appendix and EV Figures Source Data [file 44319_2024_287_MOESM13_ESM.zip › FigureEV4B/13D/Confocal image/D112N_D242N/D112N_D242N_ch00.png]

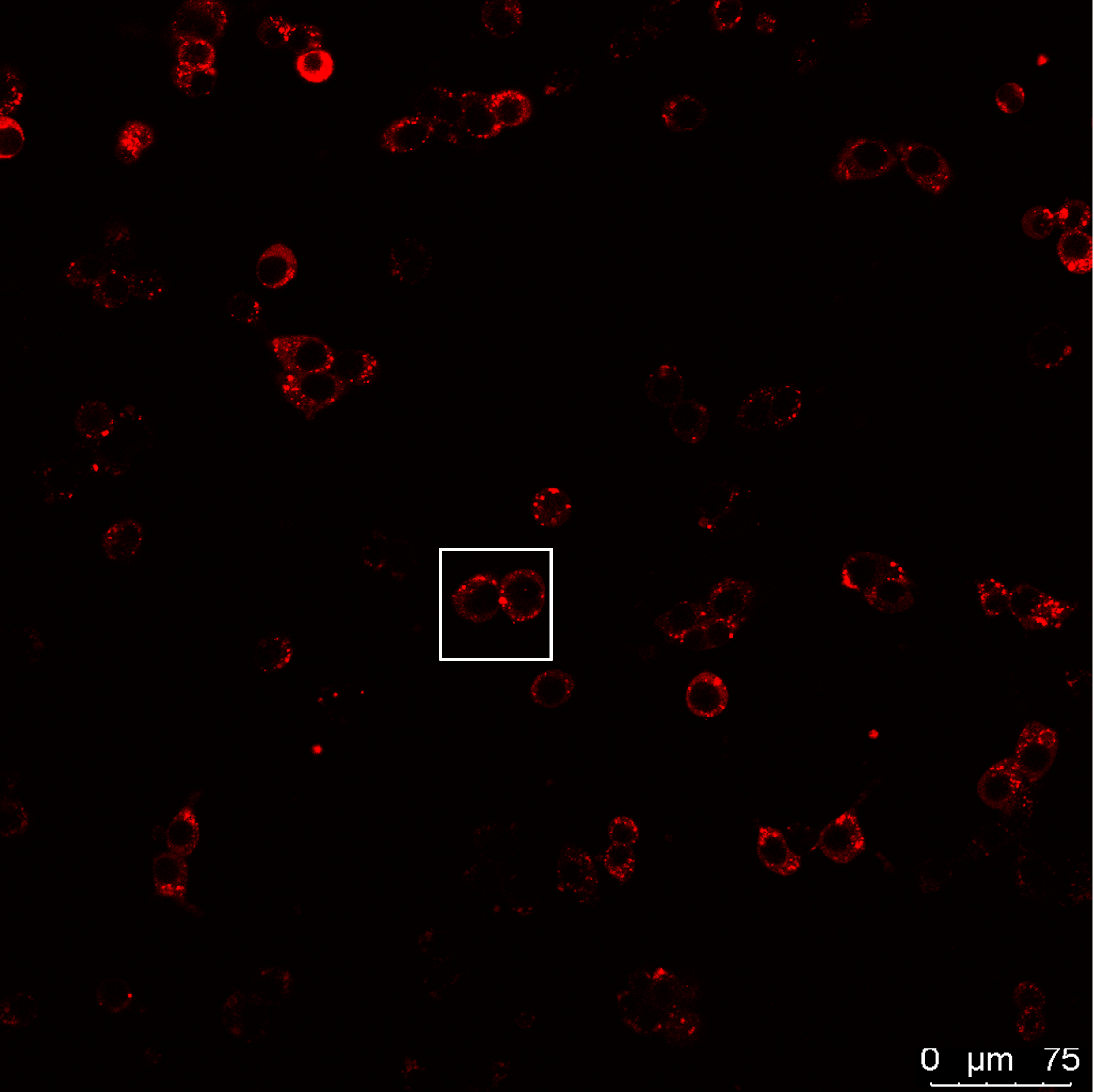

Supplement: Supplementary file 13 — Appendix and EV Figures Source Data [file 44319_2024_287_MOESM13_ESM.zip › FigureEV4B/13D/Confocal image/D112N_D242N/D112N_D242N_ch01.png]

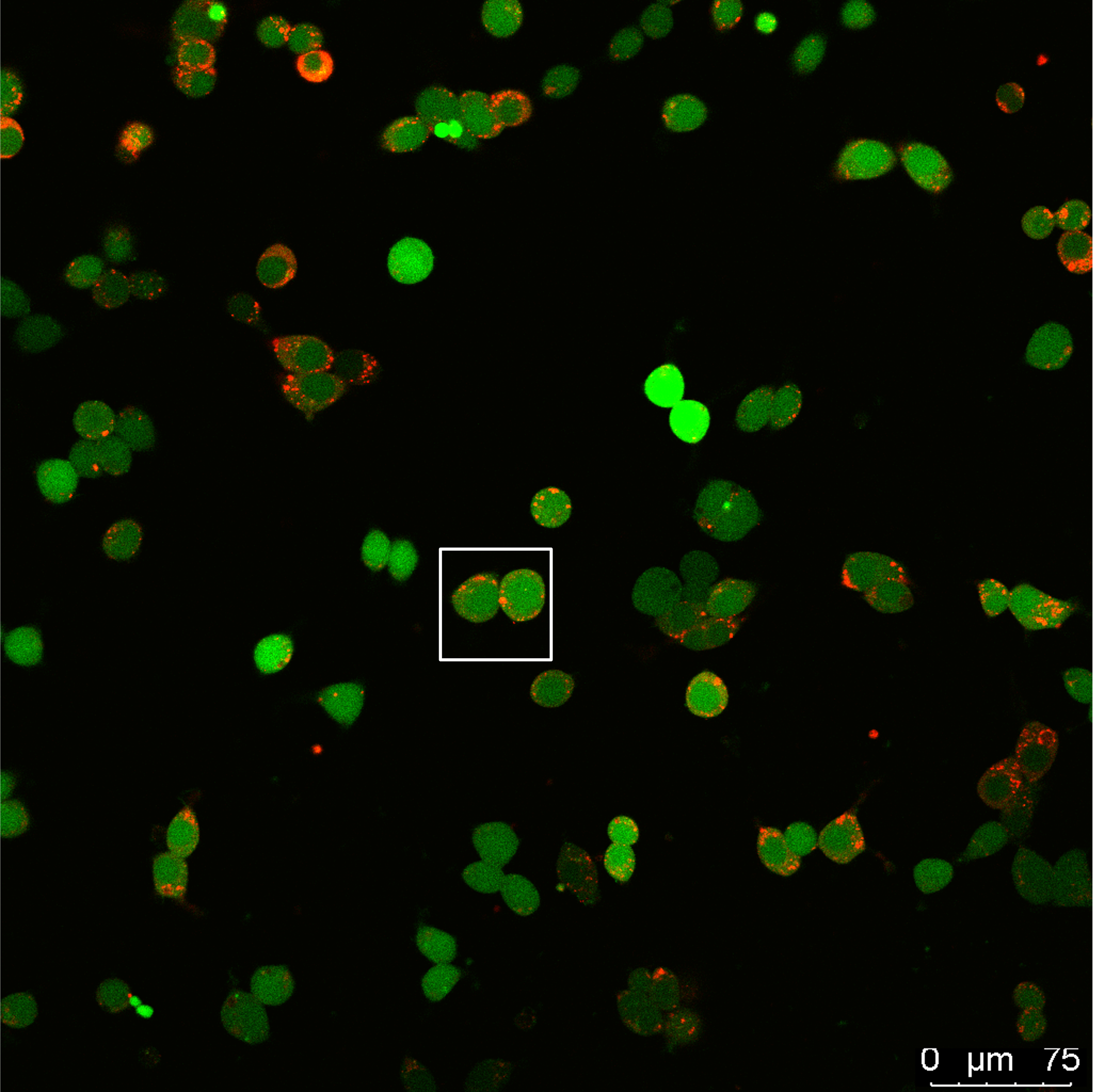

Supplement: Supplementary file 13 — Appendix and EV Figures Source Data [file 44319_2024_287_MOESM13_ESM.zip › FigureEV4B/13D/Confocal image/D112N_D242N/D112N_D242N_merge.png]

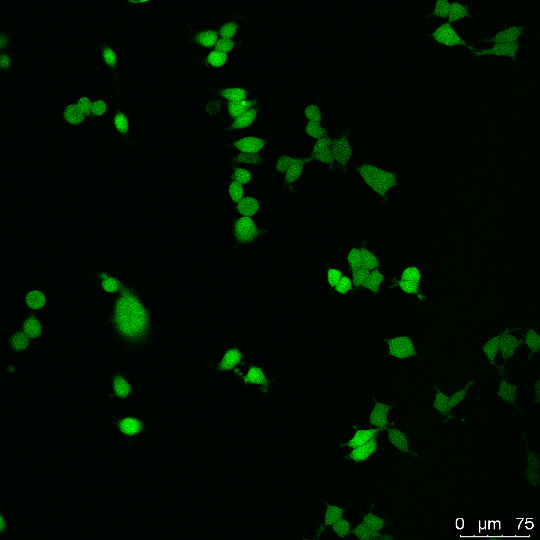

Supplement: Supplementary file 13 — Appendix and EV Figures Source Data [file 44319_2024_287_MOESM13_ESM.zip › FigureEV4B/13D/Confocal image/Empty Vector/Empty Vector_ch00.png]

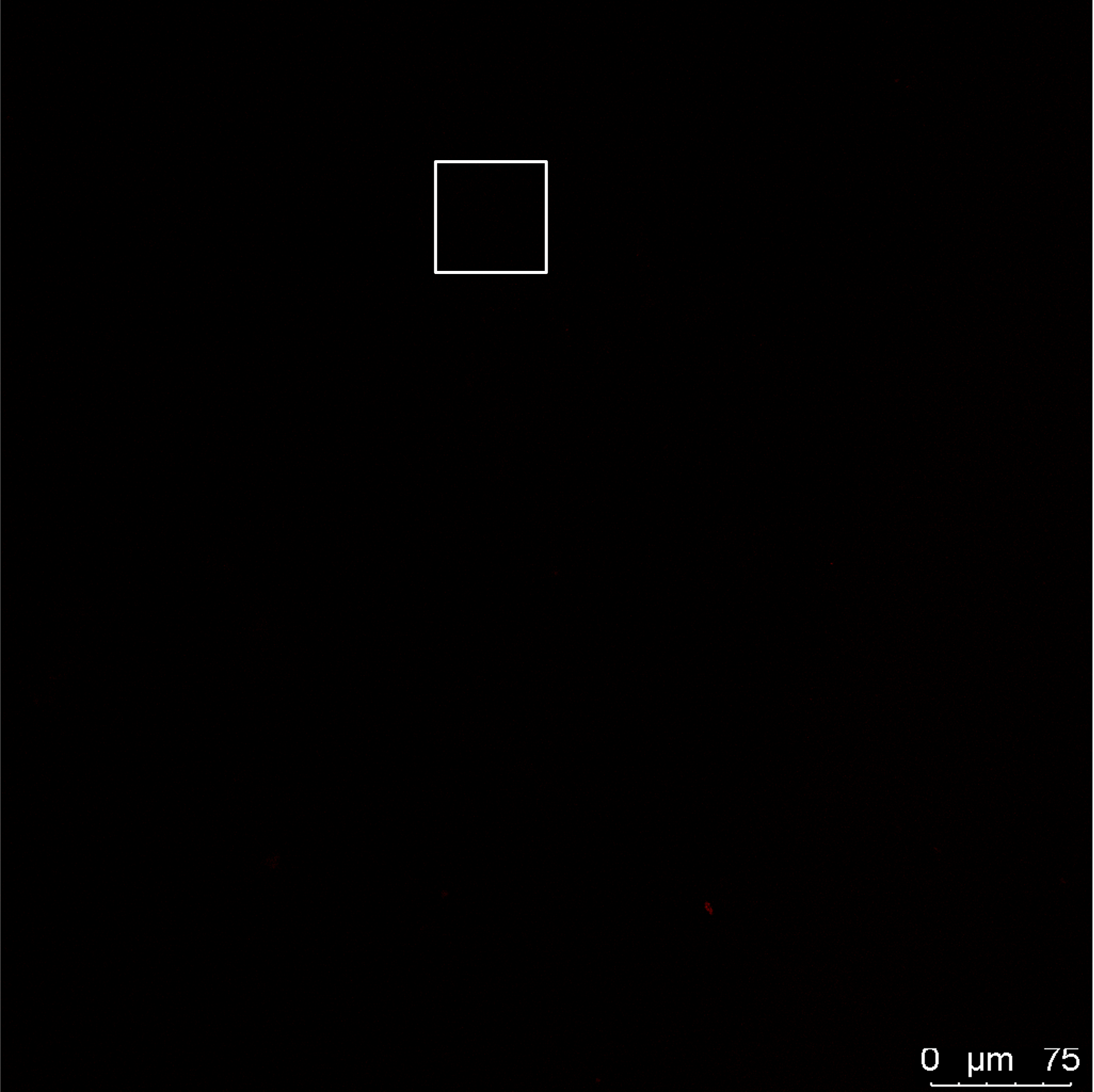

Supplement: Supplementary file 13 — Appendix and EV Figures Source Data [file 44319_2024_287_MOESM13_ESM.zip › FigureEV4B/13D/Confocal image/Empty Vector/Empty Vector_ch01.png]

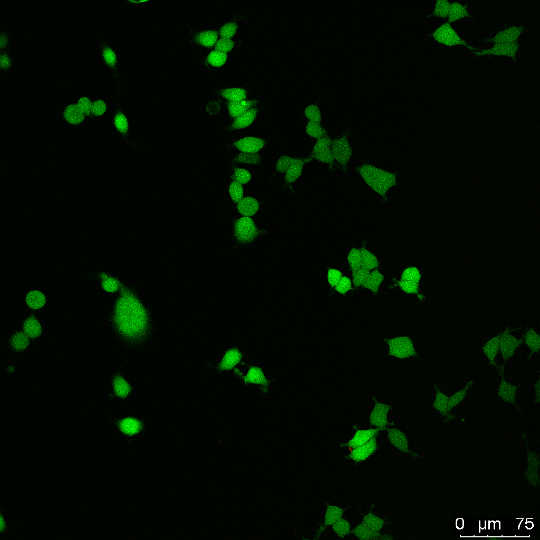

Supplement: Supplementary file 13 — Appendix and EV Figures Source Data [file 44319_2024_287_MOESM13_ESM.zip › FigureEV4B/13D/Confocal image/Empty Vector/Empty Vector_merge.png]

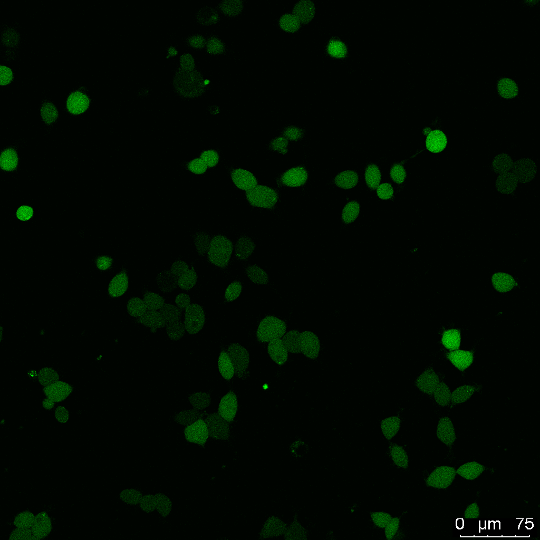

Supplement: Supplementary file 13 — Appendix and EV Figures Source Data [file 44319_2024_287_MOESM13_ESM.zip › FigureEV4B/13D/Confocal image/R286A/R286A_ch00.png]

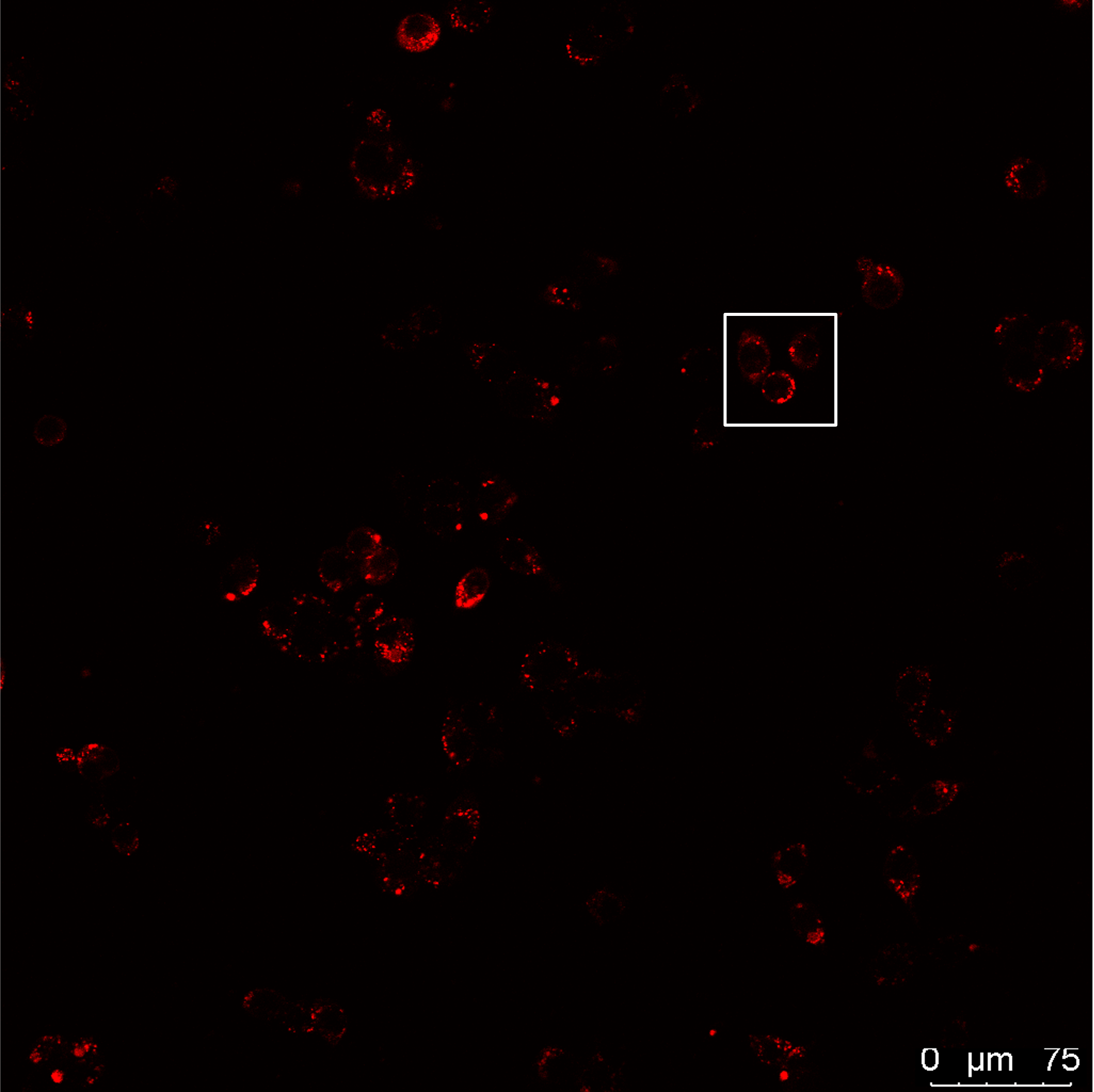

Supplement: Supplementary file 13 — Appendix and EV Figures Source Data [file 44319_2024_287_MOESM13_ESM.zip › FigureEV4B/13D/Confocal image/R286A/R286A_ch01.png]

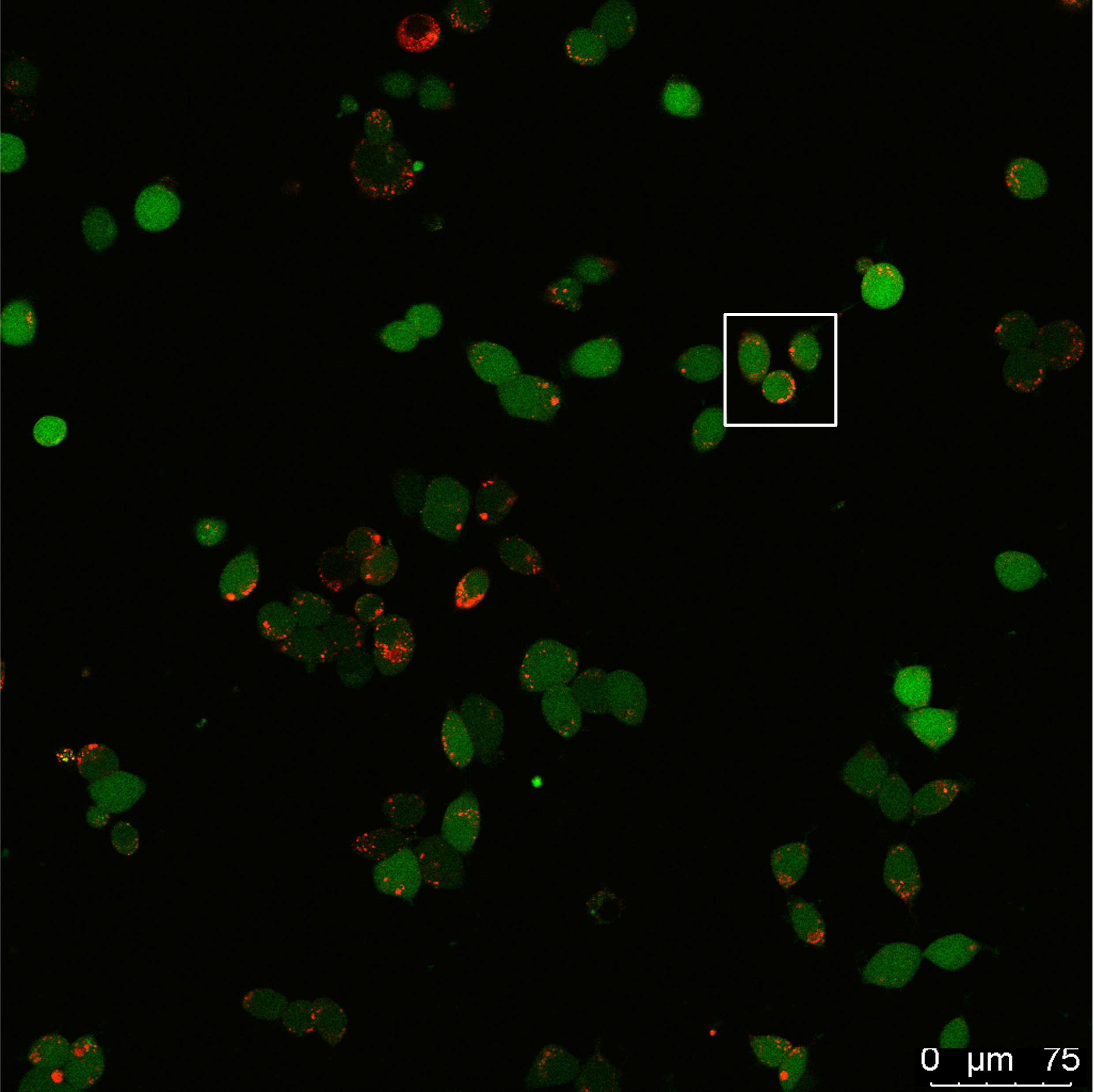

Supplement: Supplementary file 13 — Appendix and EV Figures Source Data [file 44319_2024_287_MOESM13_ESM.zip › FigureEV4B/13D/Confocal image/R286A/R286A_merge.png]

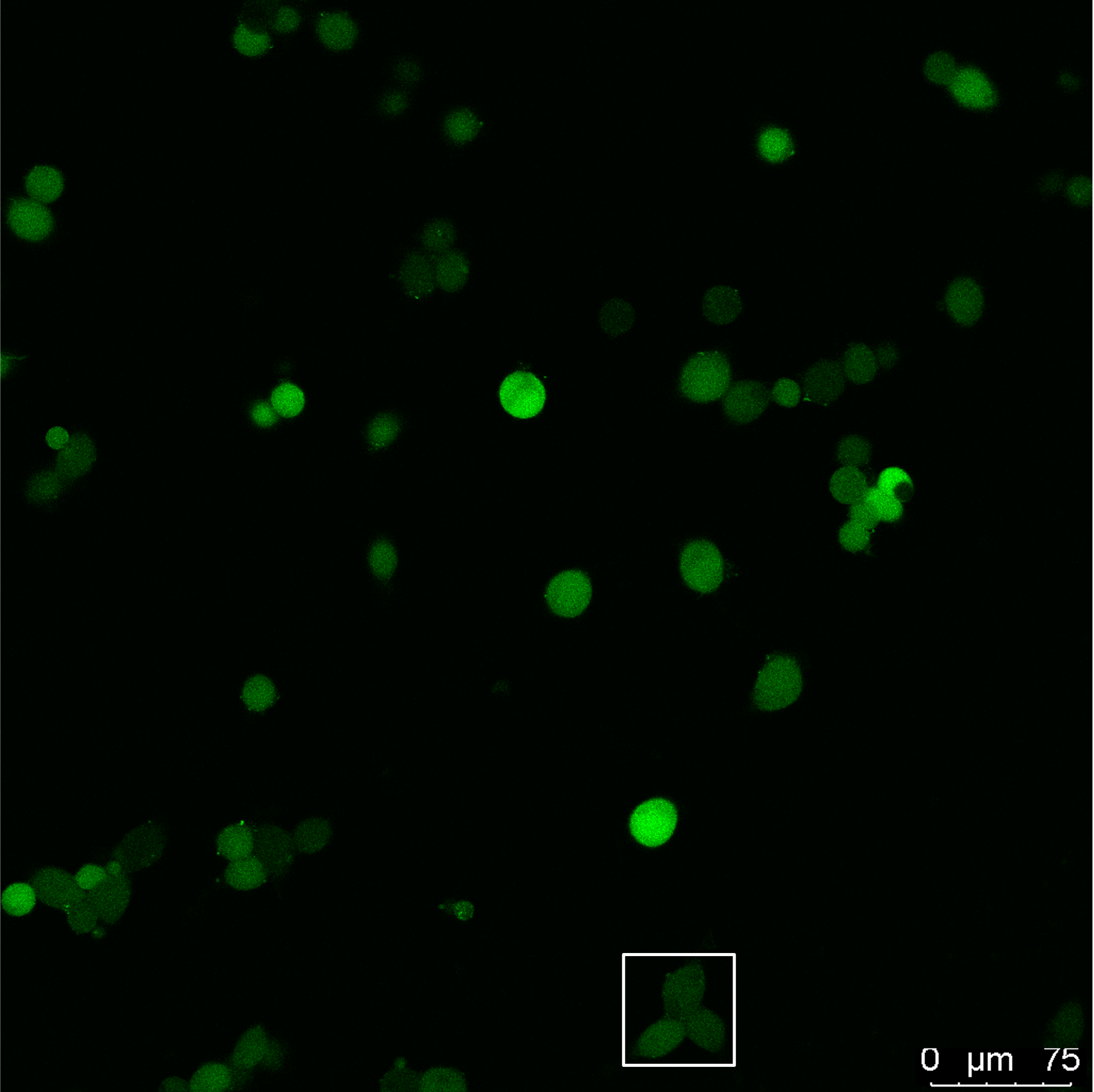

Supplement: Supplementary file 13 — Appendix and EV Figures Source Data [file 44319_2024_287_MOESM13_ESM.zip › FigureEV4B/13D/Confocal image/R286E/R286E_ch00.png]

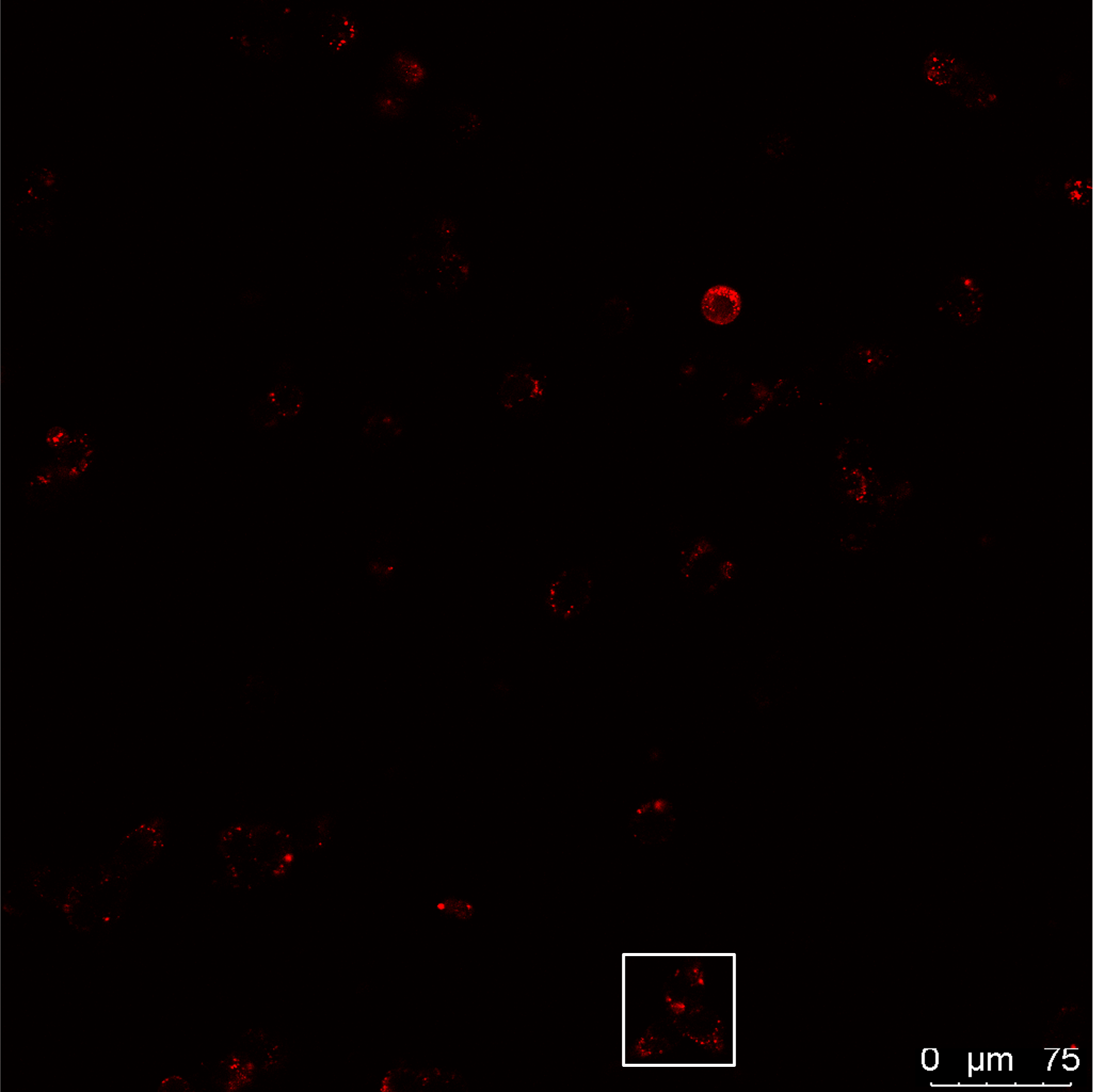

Supplement: Supplementary file 13 — Appendix and EV Figures Source Data [file 44319_2024_287_MOESM13_ESM.zip › FigureEV4B/13D/Confocal image/R286E/R286E_ch01.png]

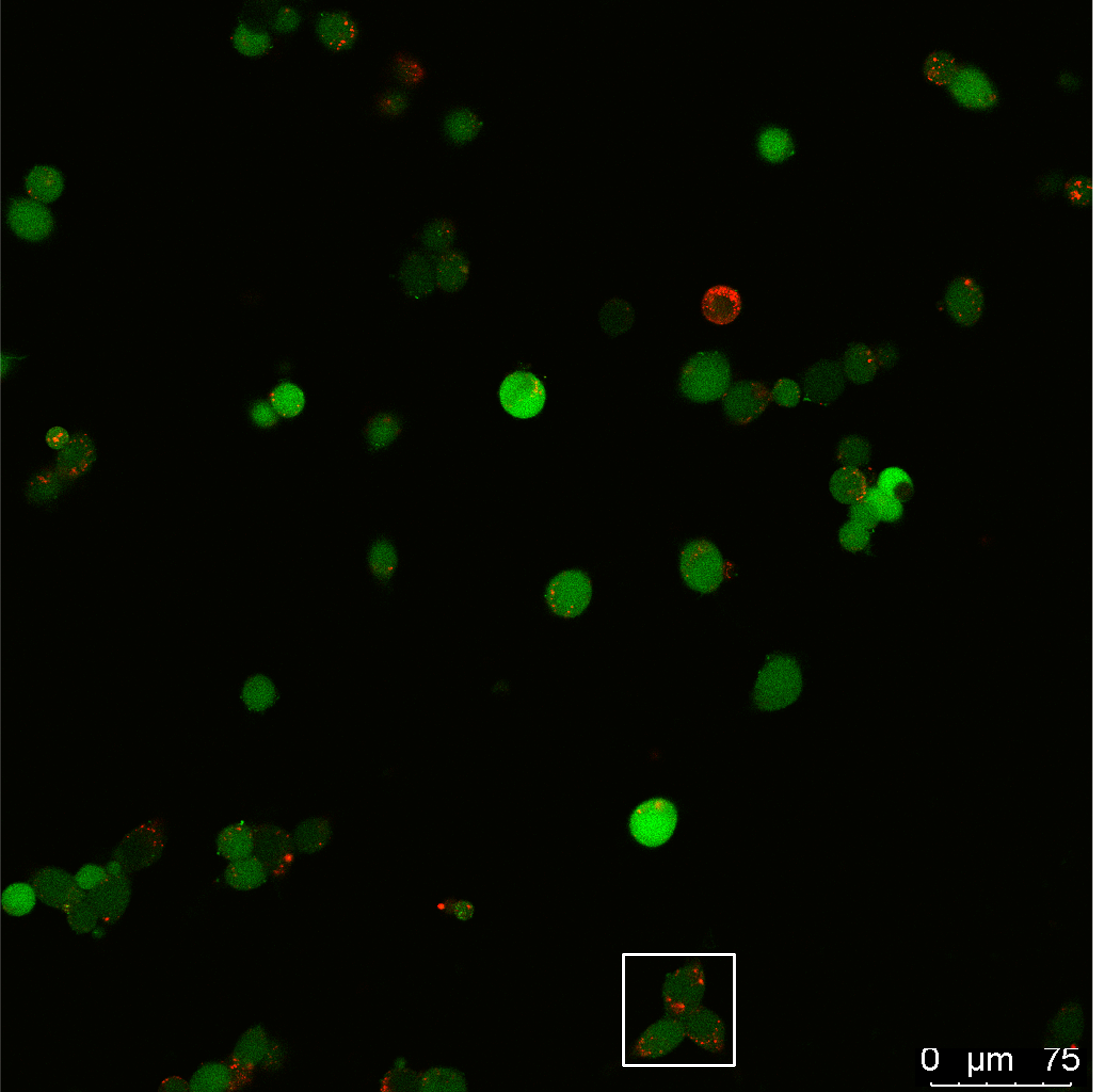

Supplement: Supplementary file 13 — Appendix and EV Figures Source Data [file 44319_2024_287_MOESM13_ESM.zip › FigureEV4B/13D/Confocal image/R286E/R286E_merge.png]

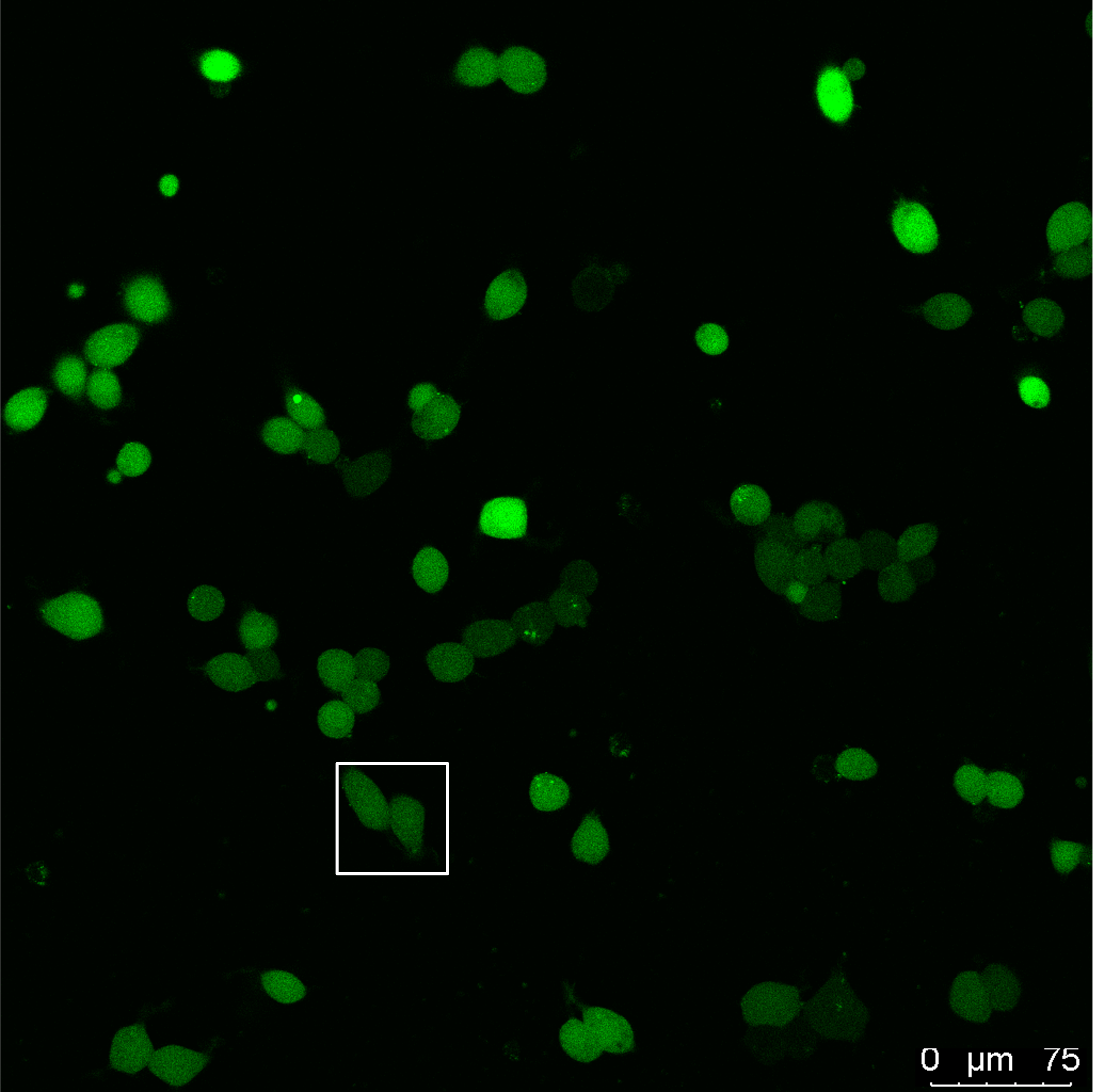

Supplement: Supplementary file 13 — Appendix and EV Figures Source Data [file 44319_2024_287_MOESM13_ESM.zip › FigureEV4B/13D/Confocal image/R298C/R298C_ch00.png]

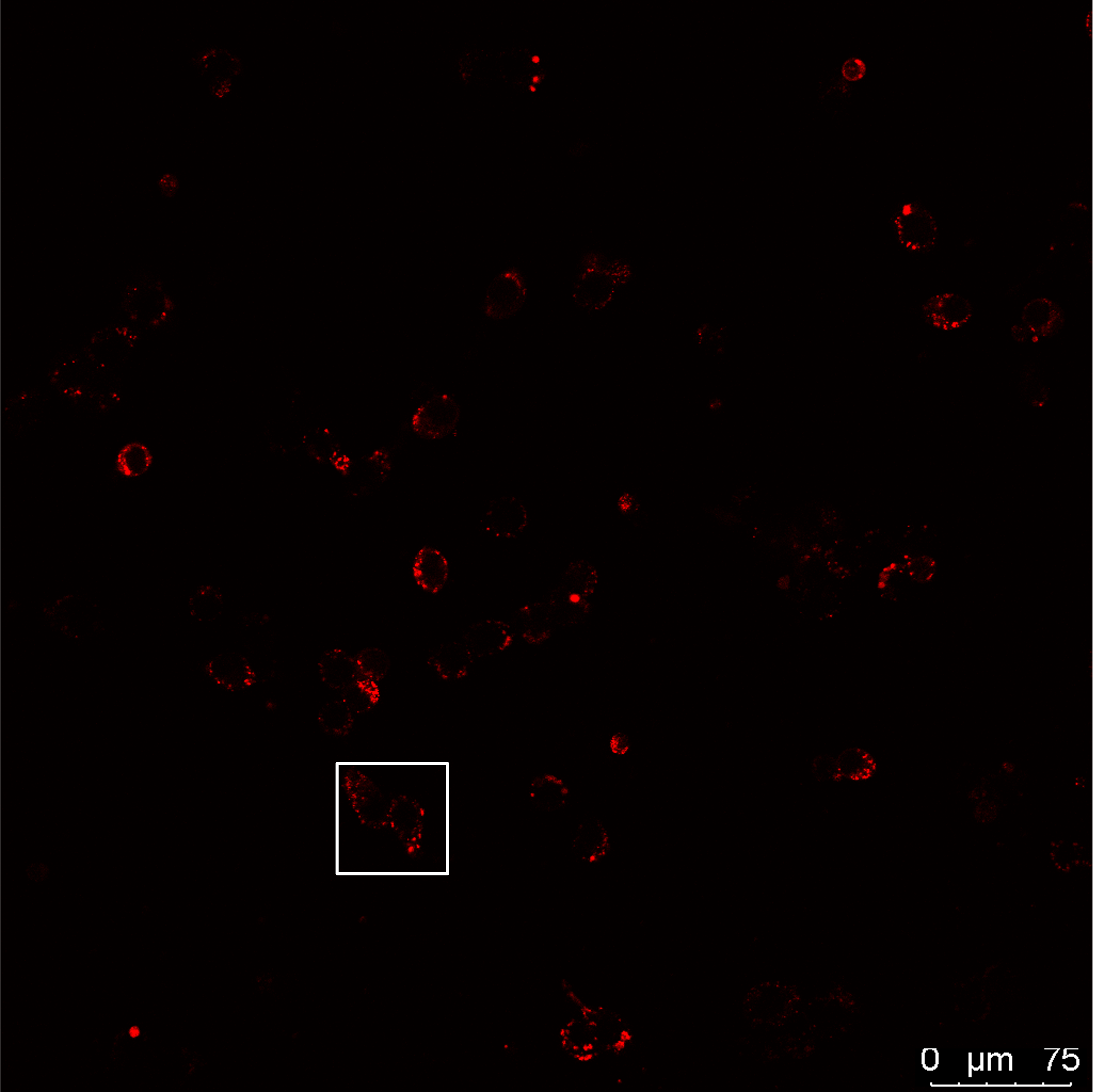

Supplement: Supplementary file 13 — Appendix and EV Figures Source Data [file 44319_2024_287_MOESM13_ESM.zip › FigureEV4B/13D/Confocal image/R298C/R298C_ch01.png]

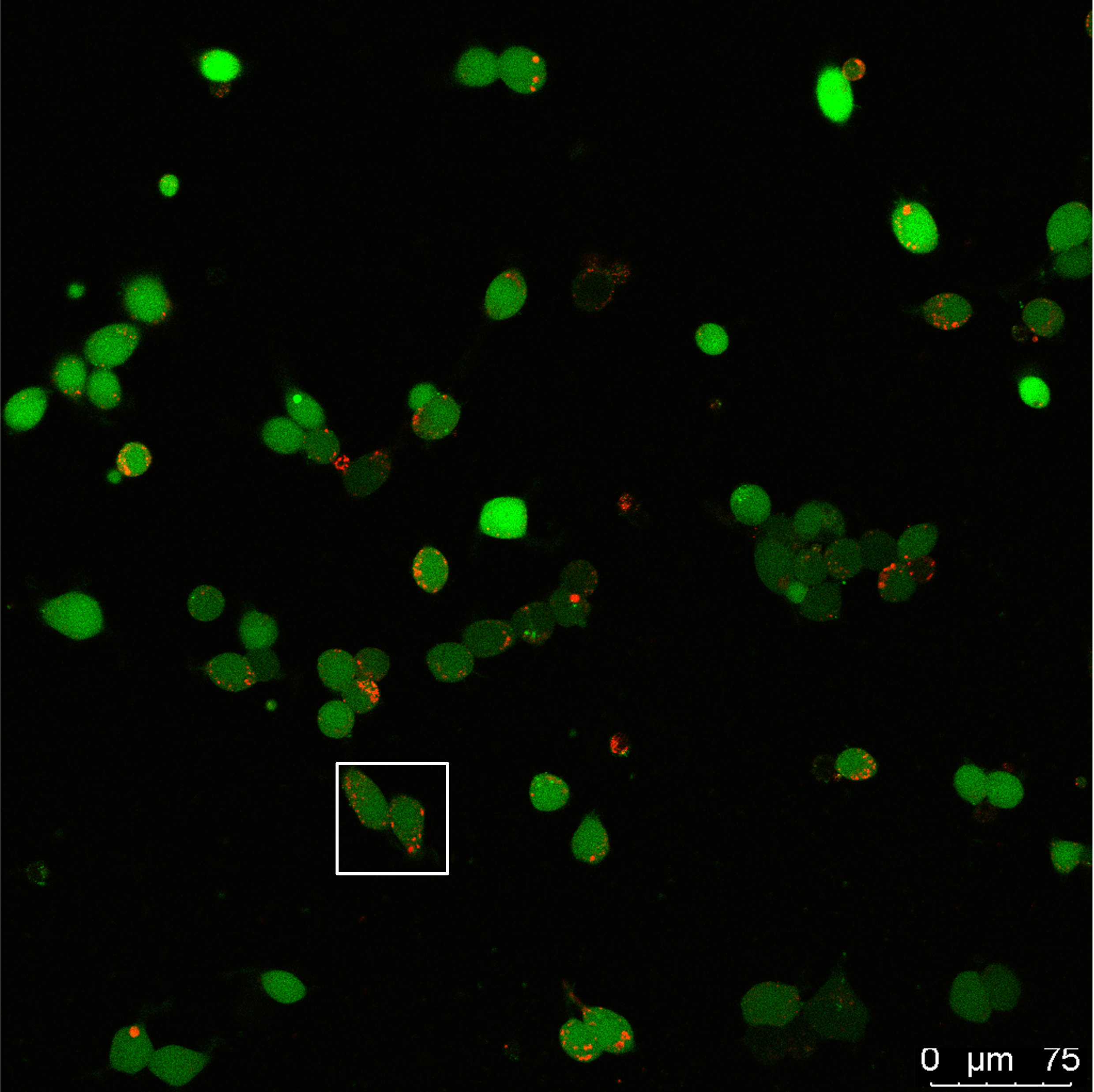

Supplement: Supplementary file 13 — Appendix and EV Figures Source Data [file 44319_2024_287_MOESM13_ESM.zip › FigureEV4B/13D/Confocal image/R298C/R298C_merge.png]

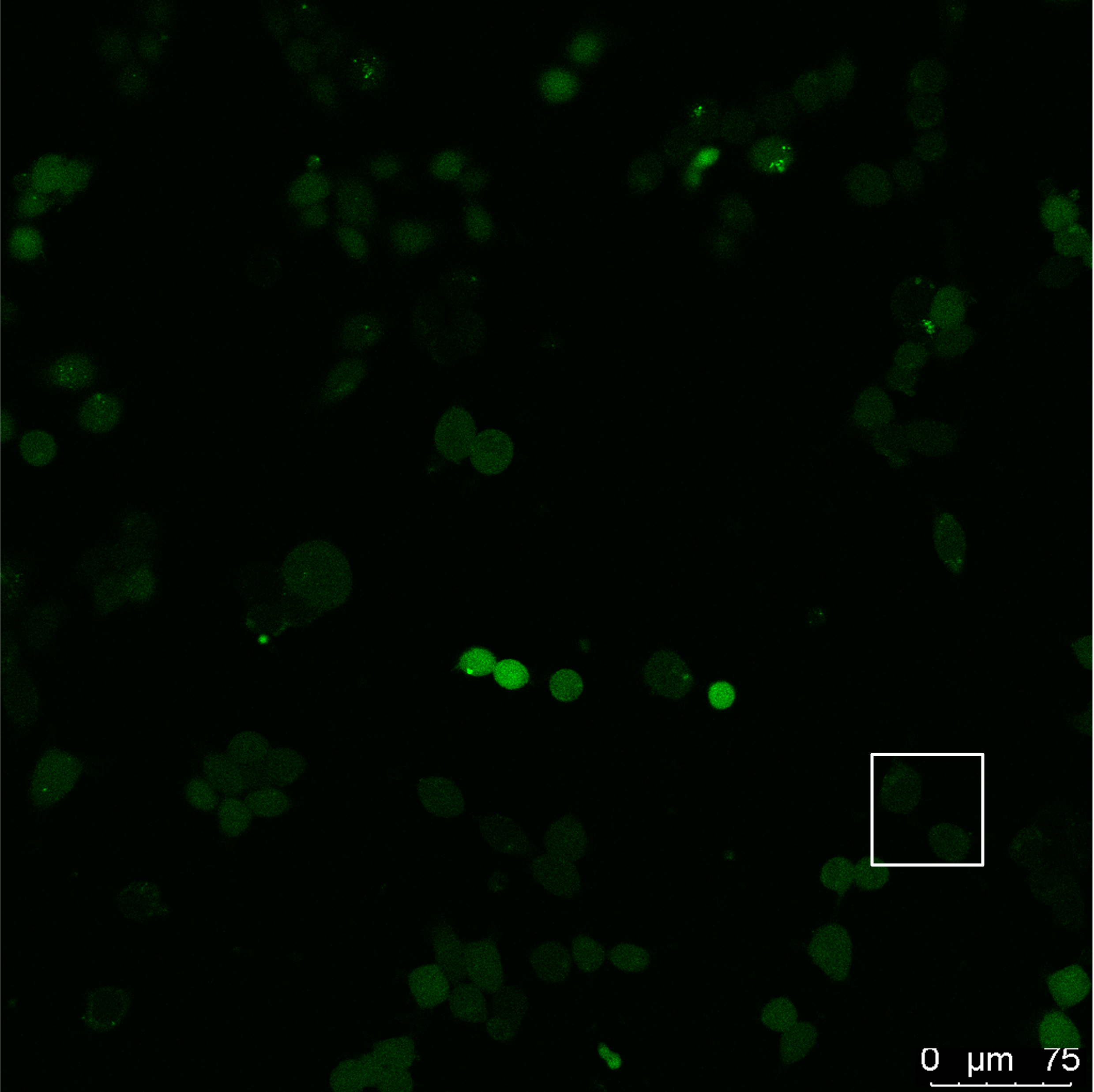

Supplement: Supplementary file 13 — Appendix and EV Figures Source Data [file 44319_2024_287_MOESM13_ESM.zip › FigureEV4B/13D/Confocal image/WT/WT_ch00.png]

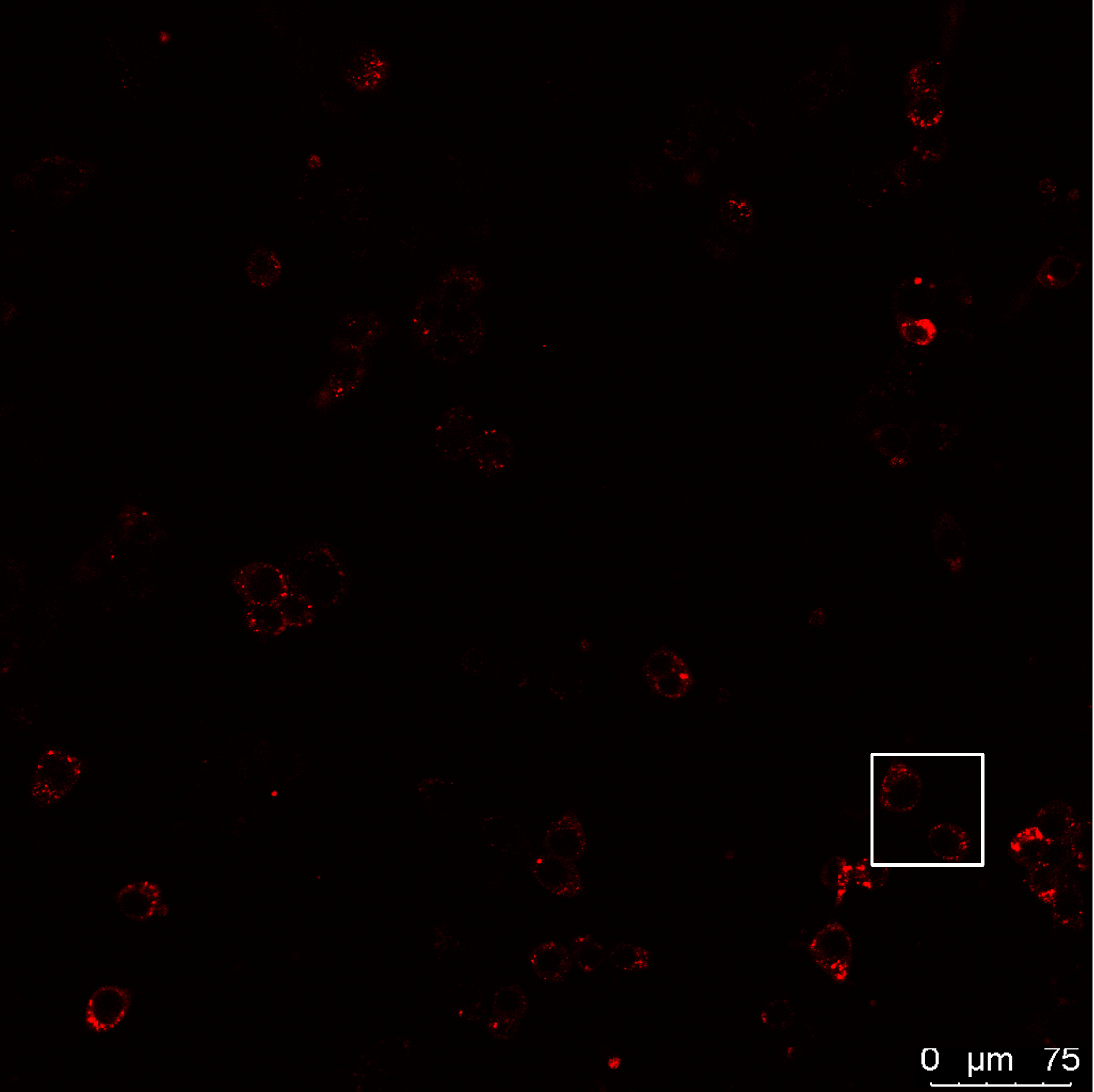

Supplement: Supplementary file 13 — Appendix and EV Figures Source Data [file 44319_2024_287_MOESM13_ESM.zip › FigureEV4B/13D/Confocal image/WT/WT_ch01.png]

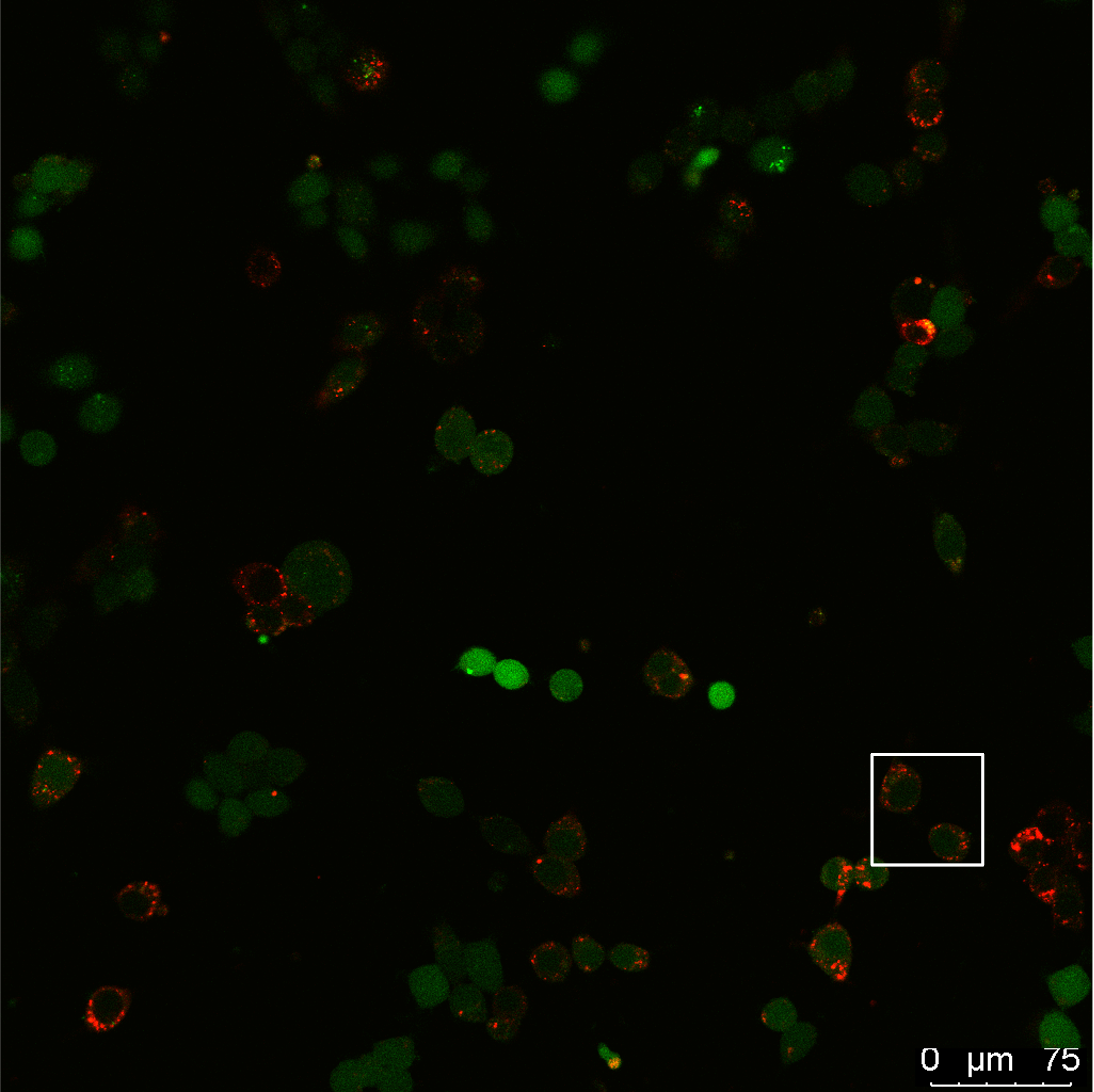

Supplement: Supplementary file 13 — Appendix and EV Figures Source Data [file 44319_2024_287_MOESM13_ESM.zip › FigureEV4B/13D/Confocal image/WT/WT_merge.png]

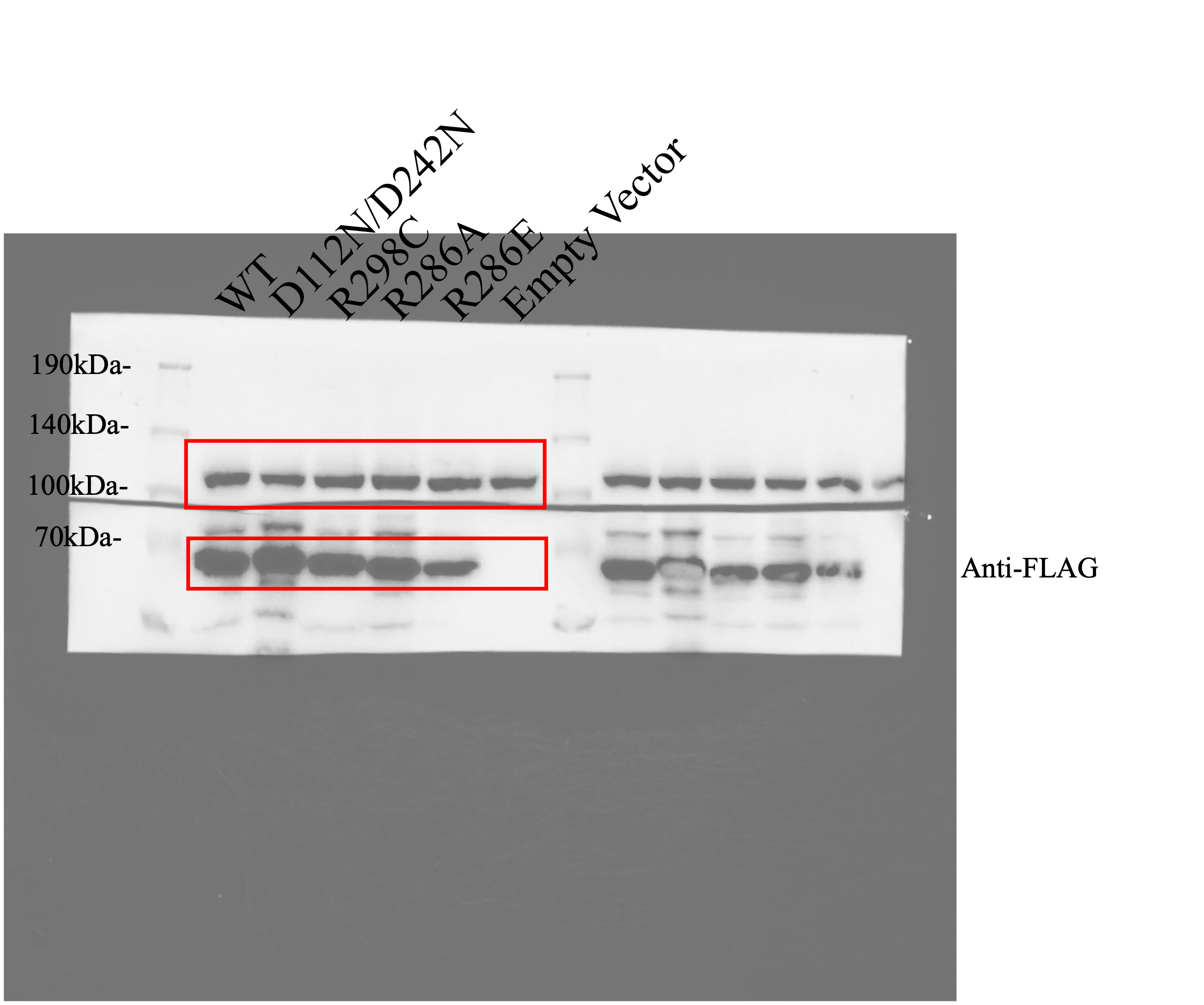

Supplement: Supplementary file 13 — Appendix and EV Figures Source Data [file 44319_2024_287_MOESM13_ESM.zip › FigureEV4B/13D/WB/ZNT3_wb.png]
