# Supplementary material for: Structural insights into human zinc transporter ZnT1 mediated Zn2+ efflux
Source: EMBO Rep. 2024 Oct 10;25(11):5006–25. doi: 10.1038/s44319-024-00287-3 (PMC11549101; doi:10.1038/s44319-024-00287-3)
Supplement: Supplementary file 14 — Expanded View Figures [file 44319_2024_287_MOESM14_ESM.pdf]

Expanded View Figures

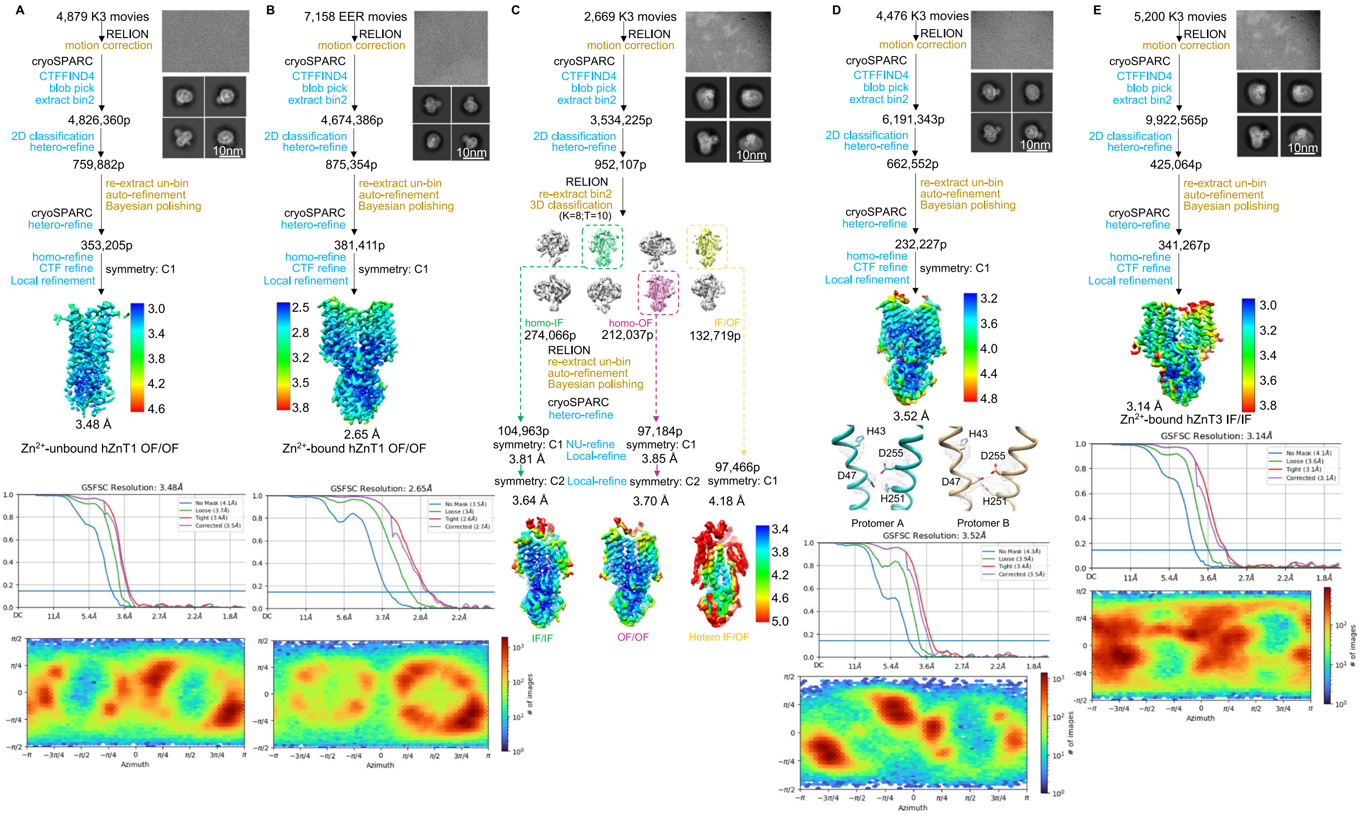

Figure EV1. Cryo-EM processing of hZnT1 and hZnT3 samples at different conditions.

(**A**) Data processing flowchart of hZnT1 in the absence of any ligand, at pH 7.5. Representative raw micrographs and 2D classifications are shown. (**B**) Data analysis flowchart of hZnT1 in the presence of 1 mM Zn^{2+}, at pH 7.5. (**C**) Processing flowchart of hZnT1 in the presence of 1 mM Zn^{2+}, at pH 6.0. (**D**) Data processing workflow of hZnT1 with 1 mM Ca^{2+} supplement, at pH 7.5. (**E**) Data processing workflow of hZnT3 with 1 mM Zn^{2+} supplement, at pH 6.0.

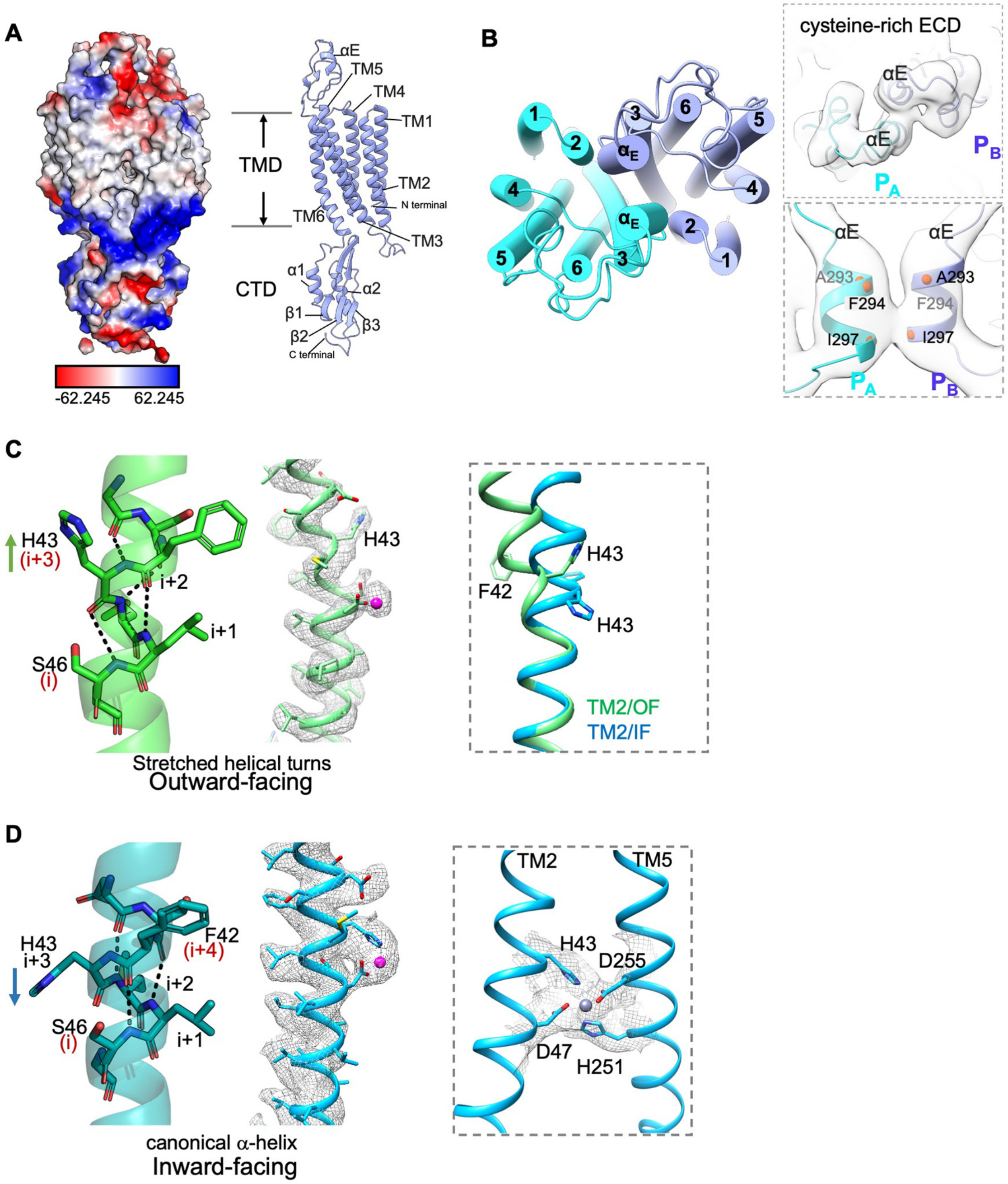

Figure EV2. Unique structural features of hZnT1.

(**A**) Surface of hZnT1 dimer is rendered in electrostatic potential. Cartoon view of one protomer, with segments labeled accordingly (right). (**B**) The extracellular cysteine-rich loops fit in unsharpened map (left), with two short helices forming the interface. (**C**) The TM2 segment around His43 residues in the Zn^{2+}-bound outward-facing dimer adopted a stretched conformation, with His43 sidechain pointing toward extracellular side. A comparison with canonical TM2 helix was shown on right. (**D**) The TM2 segment around His43 residue in the Zn^{2+}-bound inward-facing dimer adopted a canonical a-helix with His43 sidechain pointing towards the coordinated Zn^{2+}. The main-chain hydrogen bonds are shown as black dashed lines. The tetrahedral coordination network was highlighted on right with mesh density shown for the four key residues.

 © The Author(s)

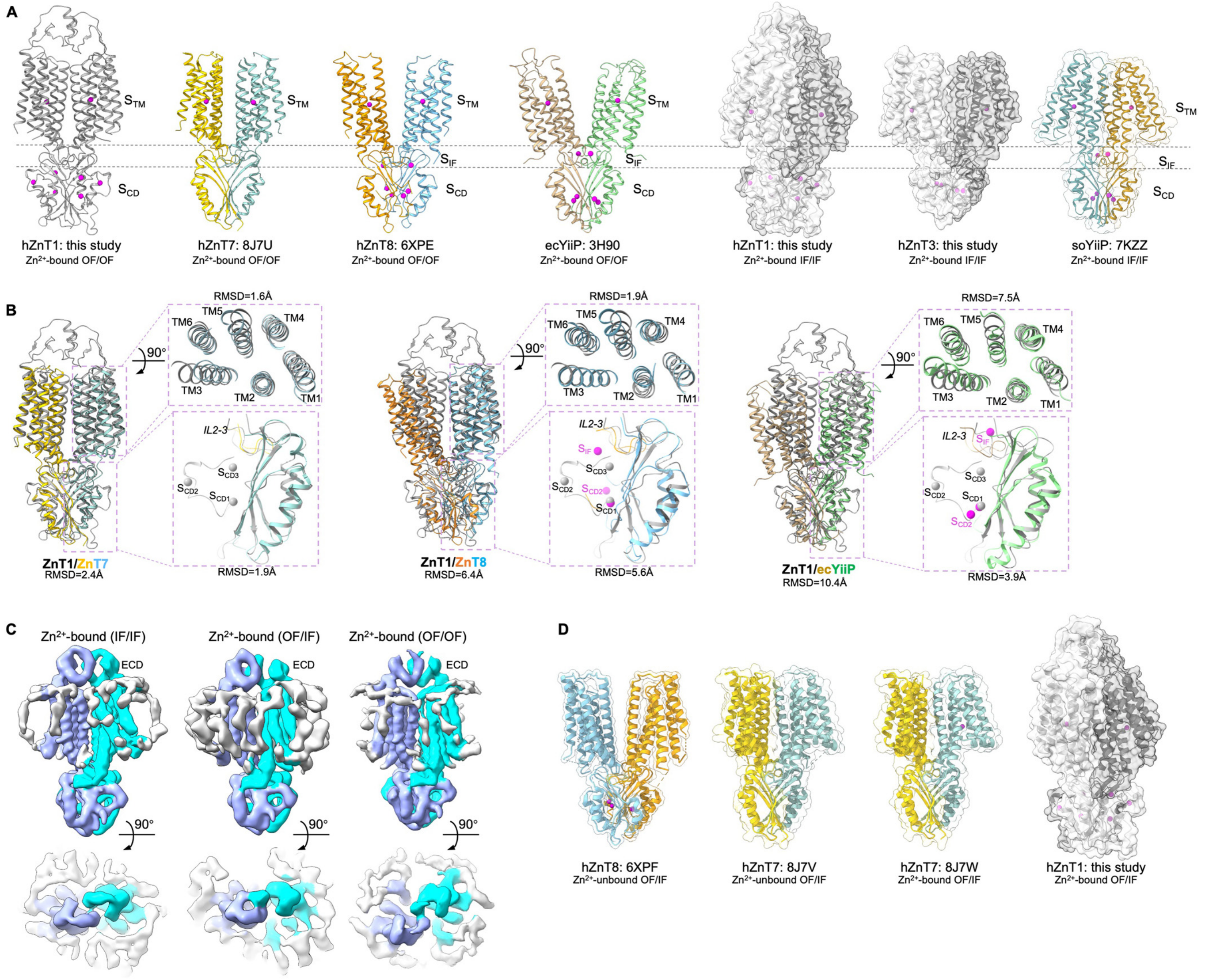

Figure EV3. Structural comparison of human ZnTs and bacterial YiiP proteins.

(**A**) Comparison of the Zn^{2+}-bound homologous outward-facing structures of hZnT1 (this study), hZnT7 (PDB: 8J7U), hZnT8 (PDB: 6XPE) and *E. coli* ecYiiP (PDB: 3H90), and inward-facing homodimers hZnT1 (this study), hZnT3 (this study), and soYiiP (PDB: 7KZZ). Zn^{2+} binding sites are labeled in accordance. hZnT1 and hZnT3 in this study were prepared similarly in LMNG/CHS detergent micelle and determined by single-particle cryo-EM. hZnT7 was prepared in GDN detergent micelle at pH 7.5 and determined by single-particle cryo-EM with a high-affinity Fab. hZnT8 was prepared in digitonin detergent micelle at pH 7.4 and determined by single-particle cryo-EM. EcYiiP was prepared in n-undecyl-β-D-maltoside at pH 7.0 and determined by X-ray crystallography. SoYiiP was prepared in n-decyl-β-D-maltoside at pH 7.5 and determined by crystallography cryo-EM. (**B**) Superimpose of hZnT1 with ZnT7, ZnT8 and ecYiiP. The TMD and CTD regions are highlighted on right, with RMSD values shown in accordance. (**C**) Comparison of the three Zn^{2+}-bound hZnT1 maps obtained at pH 6.0. EM densities are shown at the same contour level ($\sigma = 6.0$). The IF/IF homodimer presents slightly better quality of dimeric ECD region compared to OF/IF heterodimer and OF/OF homodimer. (**D**) Overall structures of the Zn^{2+}-bound OF/IF hZnT1 heterodimer (this study), Zn^{2+}-unbound OF/IF hZnT8 (PDB: 6XPF) and hZnT7 (PDB: 8J7V), as well as Zn^{2+}-bound OF/IF hZnT7 (PDB: 8J7W).

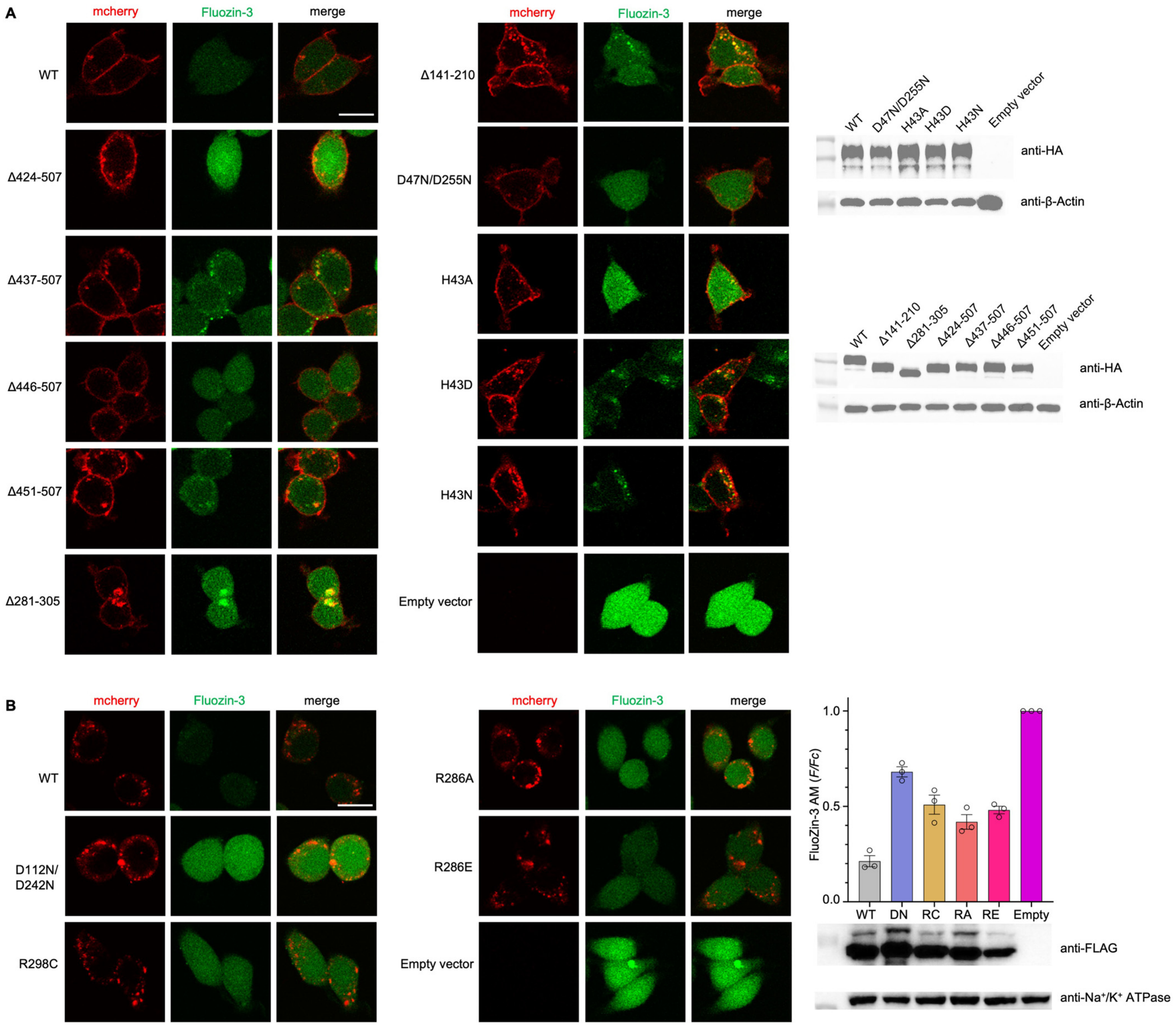

Figure EV4. Representative fluorescent images of cellular efflux activity of hZnT1 and hZnT3 WT and mutants.

(**A**) ZnT1 wild-type (WT) or mutants are fused with mCherry for visualization in stably transfected HEK293T cells. Cells with similar red fluorescence, which indicates protein expression levels, are selected for data analysis. Intracellular green fluorescence intensity was measured and analyzed in ImageJ. Cells transfected with an empty vector alone are used as control. Protein expression levels of WT ZnT1 and mutants were probed with anti-HA antibody, using internal β-actin as loading control. Scale bar size, 20 μm. (**B**) ZnT3 constructs are tagged with mCherry for visualization in stably transfected HEK293T cells. $n > 181$ total cells of intracellular FluoZin-3 AM fluorescence were analyzed for each group. Error bars indicate means ± SEM, $N = 3$ independent experiments. Total protein expression level of WT ZnT3 and mutants were measured using anti-Flag antibody, with anti-Sodium Potassium ATPase (Na^+/K^+ ATPase) as loading control. Scale bar size, 20 μm. Source data are available online for this figure.

© The Author(s)

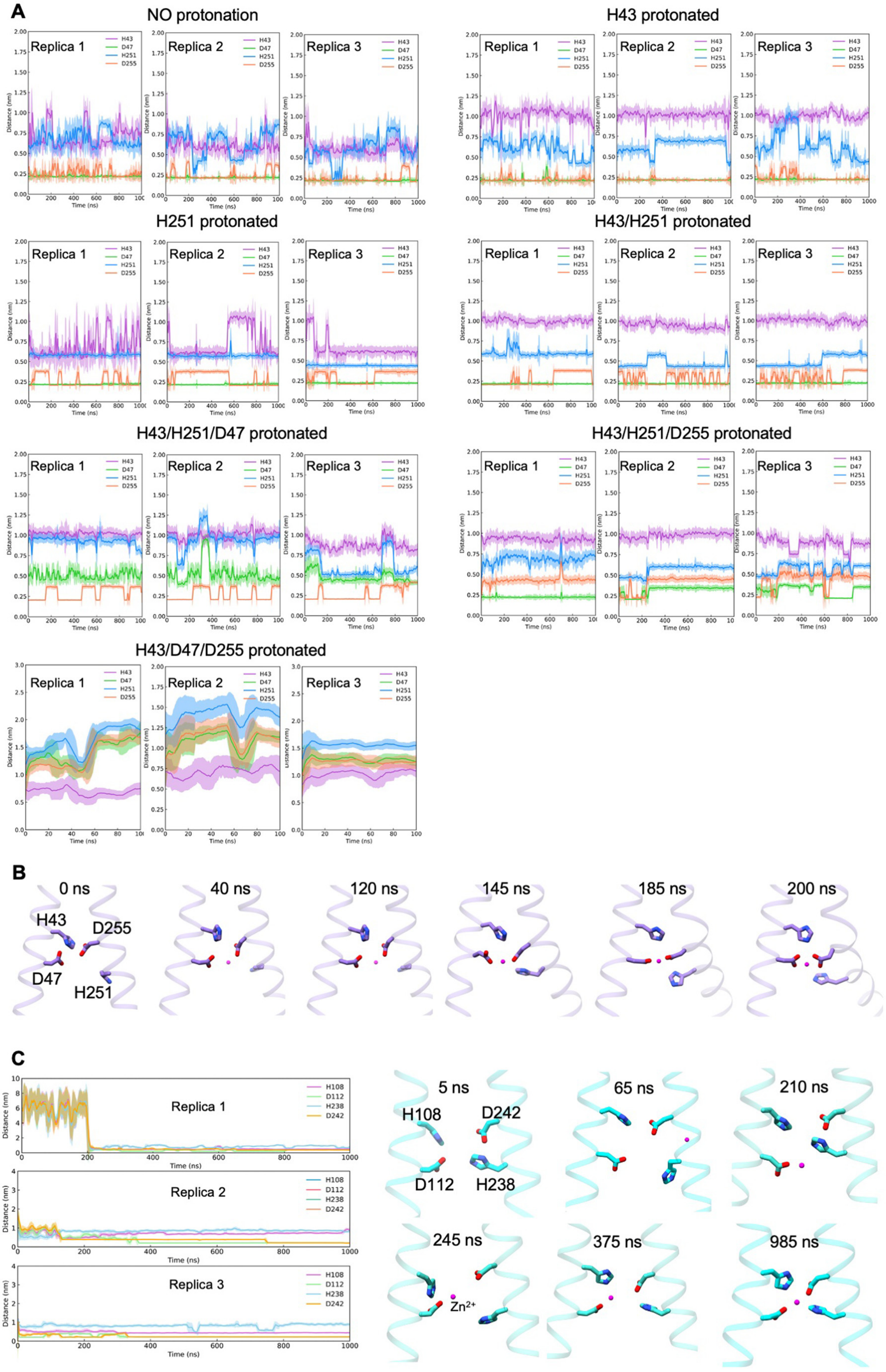

A
NO protonation
H43 protonated
H251 protonated
H43/H251 protonated
H43/H251/D47 protonated
H43/H251/D255 protonated
H43/D47/D255 protonated
Replica 1
Replica 2
Replica 3
H43
D47
H251
D255
Distance (nm)
Time (ns)
B
0 ns
40 ns
120 ns
145 ns
185 ns
200 ns
H43
D255
D47
H251
C
Replica 1
Replica 2
Replica 3
H108
D112
H238
D242
5 ns
65 ns
210 ns
245 ns
375 ns
985 ns
Zn2+

Figure EV5. MD simulation analysis of hZnT1 and hZnT3.

(**A**) The distances between center of mass of Zn^{2+} ions and sidechains of H43, D47, H251 and D255 on hZnT1, with protonation sates indicated, in three replicas. (**B**) Representative views of Zn^{2+} ion enters and binds the tetrahedral coordination network of inward-facing hZnT1 at time points indicated. (**C**) The distances between center of mass of Zn^{2+} ions and sidechains of H108, D112, H238 and D242 on inward-facing hZnT3. Representative views of Zn^{2+} ion enters and binds the tetrahedral coordination network are shown on the right.

 © The Author(s)
